# Supplementary material for: Preventing antimalarial drug resistance with triple artemisinin-based combination therapies
Source: Nat Commun. 2023 Jul 29;14:4568. doi: 10.1038/s41467-023-39914-3 (PMC10387089; doi:10.1038/s41467-023-39914-3)

**Supplementary Information** for "Preventing antimalarial drug resistance with triple artemisinin-based combination therapies"

**Supplementary Table 1.** Comparative model features

| | **PSU Model** | **MORU Model** |
|---|---|---|
| **Key representative publications** | Multiple first line therapies study in Lancet Global Health(*16*) and therapeutic efficacy by genotype study bioRxiv preprint (*30*) | Mass drug administration study in eLife (*20*) |
| **Accessibility (Either Github or description of code availability etc.)** | Code is open source at https://github.com/bonilab/malariaibm-MMC-WP2-partnerdrugresistance (version 3.2) | Code is open source at https://github.com/ATOME-MORU/malaria-model-v1.0-20.3.19. |
| **Seasonality** | Yes; not used in present analyses. | Yes; not used in present analyses. |
| **Heterogeneity in exposure** | Yes | Yes |
| **Blood-stage parasite densities modelled** | Yes | No |
| **Parameterization for clinical incidence** | Calibrated to data sets assembled for five different African studies measuring age-specific clinical incidence and EIR. No formal model fitting done for parameterization in current analysis. | We parameterized the age dependent risk of infection during the first 10 years of life using data from 8 endemic countries in sub-Saharan Africa(*40*). Given the age-dependent force of infection function, we fit the model-predicted age-dependent clinical disease incidence against two separate data sets from SE Asia (one with age, one without). Details in (*20*). |
| **Parameterization for severe disease and mortality incidence** | Mortality only. No tracking of severe malaria. Treatment failures and untreated cases are associated with 4% mortality (age groups 0-1), 2% mortality (age groups 2-5), 0.4% mortality (age groups 6-10), and 0.1% mortality (age groups 11 and older); see section 6 of supplement to(*16*). Mortality is zero for successfully treated malaria cases. | Of those clinical infections, we estimate the proportion that results in severe disease and hospitalization (by age) from data in(*41*). The dataset in (*41*) also informs the mortality rates per hospitalized case per age group. Maximum mortality rate in hospitalized children was 5% (<2 year olds). Older children had a greater treatment success rate with mortality rates of less than 0.5%. |
| **Drug interventions – PK- PD Drug action etc** | Single compartment PK model used, with daily parasite killing, as a function of drug concentrations and parasite genotypes, for PD model. | Parasite clearance modelled using PD data from parasite clearance studies, with daily parasite killing, as a function of initial drug concentration, drug IC50 and parasite genotype. |
| **Genotype tracking and sensitivity to drugs** | Yes, four key resistance loci and two copy number variants tracked. 64 total genotypes. | Yes, four key resistance loci and two copy number variants tracked. 64 total genotypes. |

| | | |
|---|---|---|
| **Vector control Interventions** | Indirect vector control only, via transmission parameter that determines the daily amount of biting. | Yes – LLIN, IRS, Larval Control |
| **Treatment interventions** | Yes, many types of drug policies such as multiple first-line therapies, cycling, adaptive cycling, triple therapy, mass drug administration, and private-market drug sales. | Yes, treatment of clinical disease, MDA, MSAT, adjunctive primaquine, TME, iPT, and private market-drug sales. |
| **Treatment seeking and drug coverage** | Explored in the sensitivity analysis, at 25%, 50%, and 75% coverage. | Explored in the sensitivity analysis, at 25%, 50%, and 75% coverage. |
| **Spatial dynamic model** | Present analyses run in a single location. | Present analyses run in a single location. |
| **Super-infections, co-infections, multiplicity of infection** | Yes, tracked explicitly as coinfections arising from additional bites on already infected individuals. | Yes, each human individual can carry up to 10 different parasite populations (one per inoculum). Each inoculum is considered to be a clonal population upon emergence from the liver, with one parasite acquired from each infectious bite. |
| **Heterogeneity in Exposure** | Exposure varies both by age and between individuals. | Exposure varies both by age and between individuals. |
| **Duration of infection** | Not drawn from a predetermined distribution. The duration of infection is determined by explicit modelling of parasitaemia and how drugs and the immune system act on parasites. Calibrated to malariatherapy data. Durations of infection in the model range from 60 to 281 days. | Asymptomatic infection duration based on malariatherapy data and clinical follow-up data from endemic areas, as well as drug efficacy data (PD). Also estimated from a set of 8 Endemic areas from Sub-Saharan Africa(*40*). |
| **Clinical disease and history of exposure** | A proportion of infected individuals go on to develop clinical disease. Immunity to clinical disease develops with exposure and age. Simulation also has a maternally acquired component. | Proportion developing clinical disease depending on cumulative immunity from prior exposures and immunity level (indicator of recent exposure). |
| **Decay of natural immunity** | Exponential decay of naturally acquired immunity. | Exponential decay of naturally acquired immunity as estimated in (*40*) |
| **Infectiousness and gametocyte models** | Human infectiousness to mosquitos is a function of asexual parasite density, with a time lag built in to model the fact that infectious gametocytemia lags asexual parasitaemia. | Infectiousness depends on lagged development of sexual stage parasites and seasonal transmission equation from fitting to incidence data. It is informed by parasite density in an indirect way: clinical individuals are assumed to have a higher mean infectiousness compared to asymptomatic individuals as they carry higher parasite density loads. |
| **Entomological models** | 11-day lag built in so that FOI today depends on the biting done by mosquitoes 11 days ago. No other entomological built-in features. | Full IBM component to mosquito dynamics. For this exercise we use a simplified version where only infectious mosquitoes are tracked individually. |
| **Recombination model** | Recombination can occur in a mosquito bite on a multi-clonal host. Parasites are taken up by the mosquito in proportion to their parasite density. A full recombination table is built for all possible forces of infection resulting from this host's contribution to the next generation of infectious | No recombination. No interrupted feeding. |

| | | |
|---|---|---|
| | sporozoites, according to the normal rules of Mendelian genetics. No interrupted feeding allowed in present analyses. | |
| **Mutation model** | No back mutation.  Mutation can occur during treatment only when the mutation confers a resistance benefit to the current treatment. | No back mutation. Mutation can occur during treatment (higher rate), and in the absence of treatment (lower rate). The higher rate was calibrated during the calibration exercise (*33*) |
| **Stochasticity** | All model components are stochastic and described by defined probability distributions defined in (*16*) and (*30*). The only non-stochastic elements are the delay from mosquito oocyst formation to rupturing, which is modelled as a fixed duration. | All model components are stochastic and described by defined probability distributions defined in (*20*). |
| **PfPR range models calibrated against.** | Immunity-symptom relationship calibrated to data sets where PfPR > 5%, across an EIR range of 10 to 200 (*16*). | Calibrated based on 8 data sets with a $PfPR_{2\text{-}10}$ minimum of 2% (*40*). |

## Supplementary Figures

**Supplementary Fig. 1.** Simulated therapeutic efficacy study using the Penn State model's single-compartment PK/PD model and the EC50 parameterizations from (*2*). A total of 20,000 patients with baseline parasitaemia of 2000 parasites/µl to 200,000 parasites/µl (distributed uniformly on the $\log_{10}$-scale of 3.3 to 5.3) were given a standard 3-day regimen of each drug (*n*=10,000 patients), without any loss to follow-up. The density plots show the asexual parasitaemia distribution among the 10,000 patients each day following treatment. Three solid orange lines mark the 99$^{th}$, 98$^{th}$, and 97$^{th}$ percentiles of those distributions, and the two dashed orange lines show the 95$^{th}$ and 90$^{th}$ percentiles. Orange percentile markers are not visible for all days as they may overlay each other at 0.00002 parasites/µl. The therapeutic efficacy for each drug is given as a percentage above the last day's density plot and illustrates the percentage of patients with a parasitaemia lower than 10 parasites/µl on day 28 following treatment.

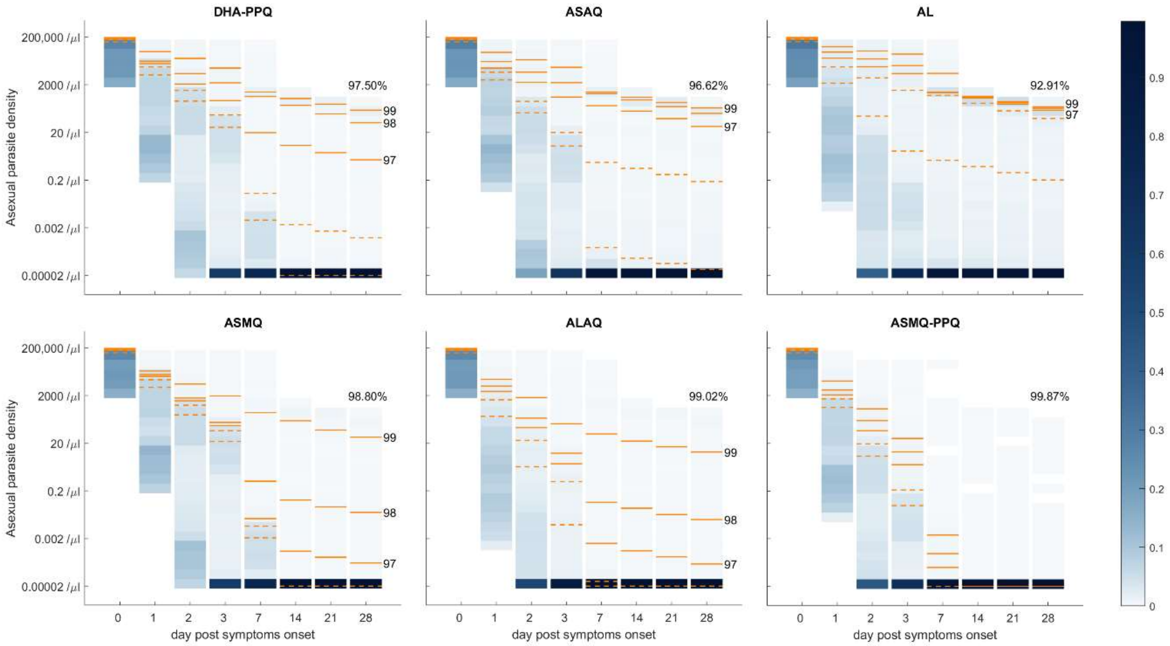

**Supplementary Fig. 2. 580Y allele frequency over time.** Panels show the evolution of 580Y allele frequency in a population of 1,000,000 individuals, in the years following a switch of first line therapy to TACTs. Each panel corresponds to a combination of transmission intensity: 0.1% PfPR (top row), 1% PfPR (middle row), 10% PfPR (bottom row) and baseline ACTs used before TACTs are deployed at year zero: DHA-PPQ (left column), ASAQ (middle column), AL (right column). Median trajectories are shown from 100 simulations, and the shaded area shows the range from the 5th to the 95th percentile. Grey lines show the evolution of the 580Y allele frequency under continued baseline ACT use. Red lines and blue lines show the evolution of the 580Y allele frequency after the deployment of ASMQ-PPQ and ALAQ, respectively. Figure title shows model used, outcome tracked, and treatment coverage (TC) level.

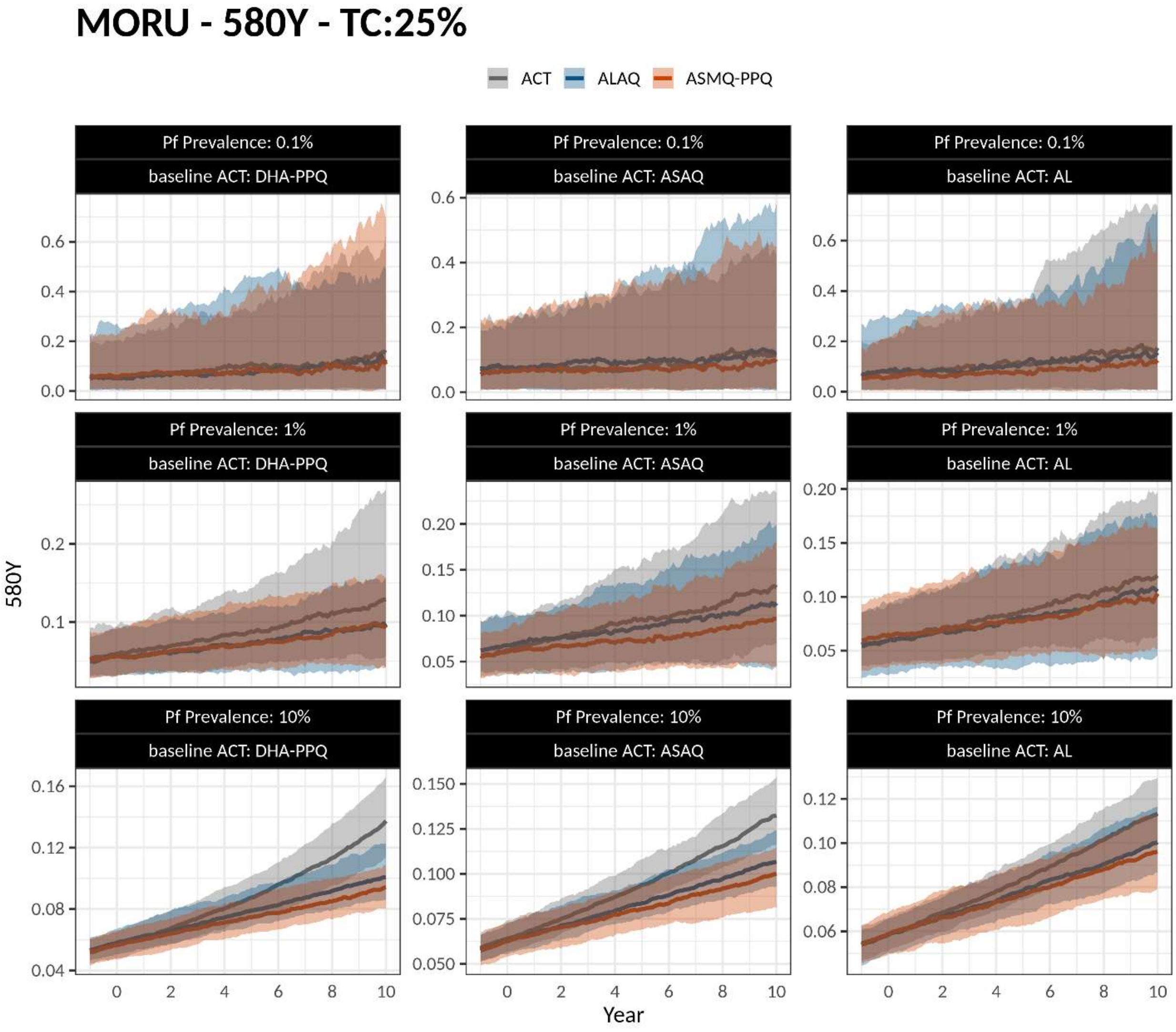

**Supplementary Fig. 3.** Simulations settings as in Supplementary Fig. 2. Figure title shows model used, outcome tracked, and treatment coverage (TC) level. Median trajectories are shown from 100 simulations, and the shaded area shows the range from the 5th to the 95th percentile. Grey lines show the evolution of the 580Y allele frequency under continued baseline ACT use. Red lines and blue lines show the evolution of the 580Y allele frequency after the deployment of ASMQ-PPQ and ALAQ, respectively.

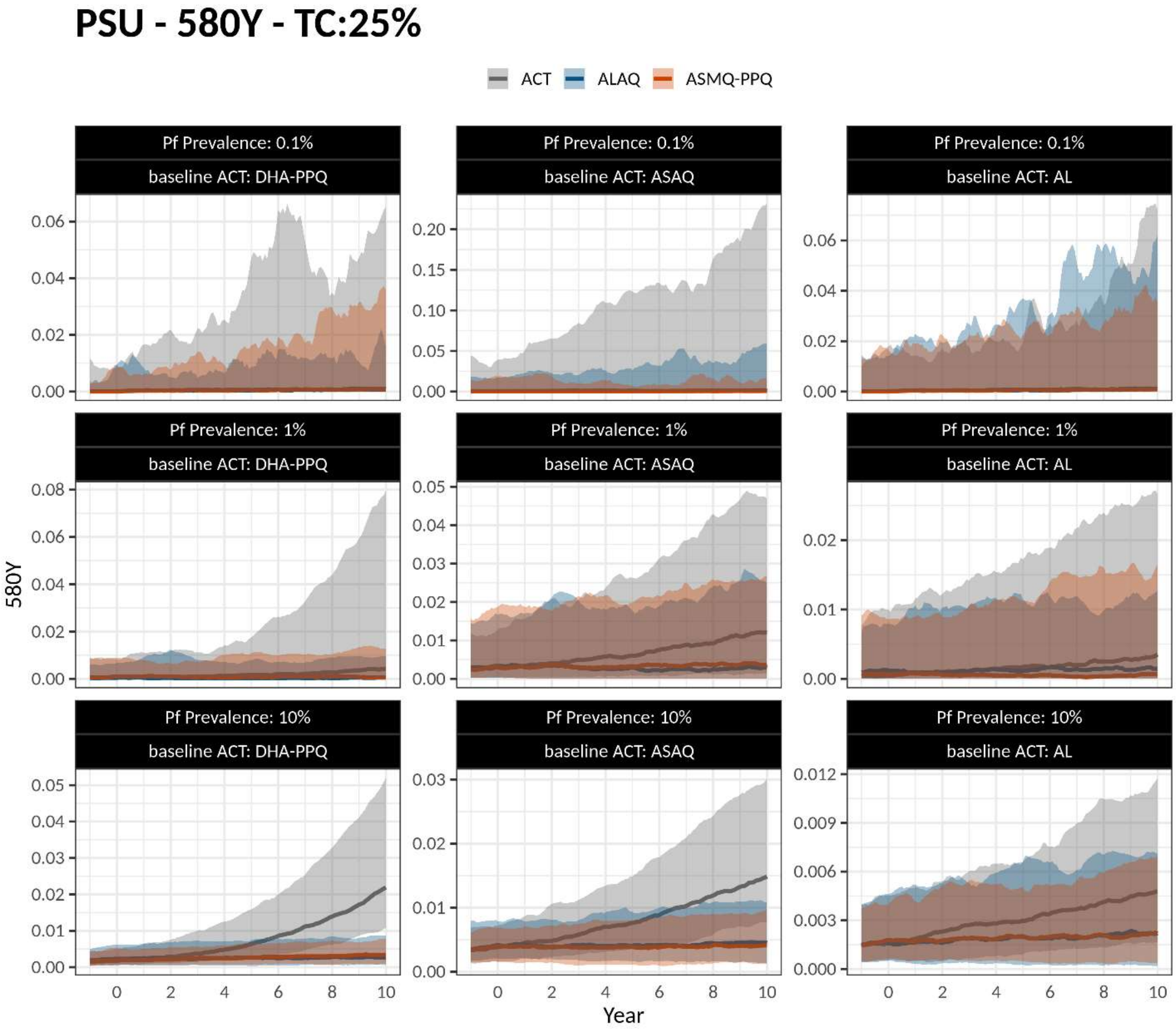

**Supplementary Fig. 4.** Simulations settings as in Supplementary Fig. 2. Figure title shows model used, outcome tracked, and treatment coverage (TC) level. Median trajectories are shown from 100 simulations, and the shaded area shows the range from the 5th to the 95th percentile. Grey lines show the evolution of the 580Y allele frequency under continued baseline ACT use. Red lines and blue lines show the evolution of the 580Y allele frequency after the deployment of ASMQ-PPQ and ALAQ, respectively.

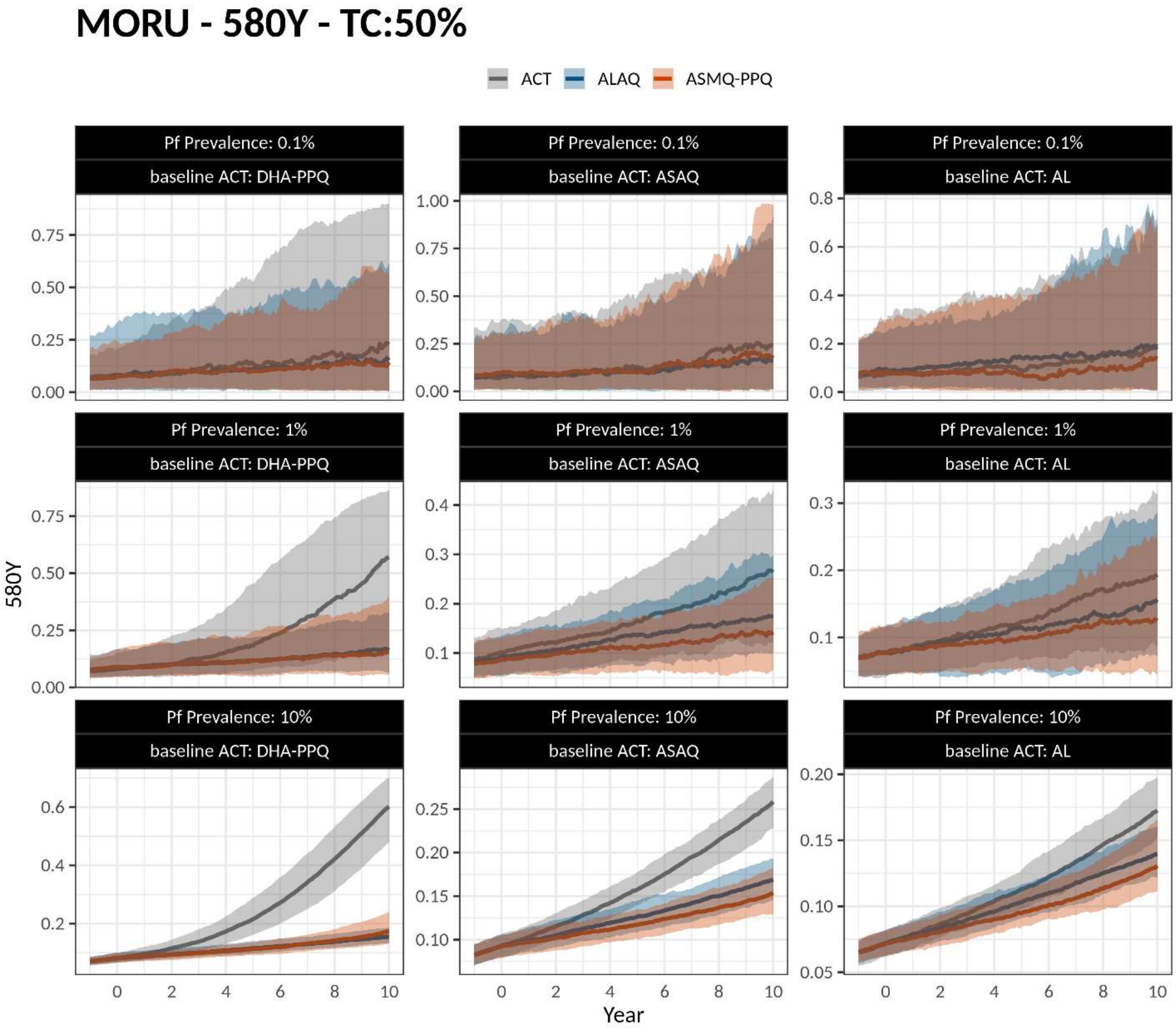

**Supplementary Fig. 5.** Simulations settings as in Supplementary Fig. 2. Figure title shows model used, outcome tracked, and treatment coverage (TC) level. Median trajectories are shown from 100 simulations, and the shaded area shows the range from the 5th to the 95th percentile. Grey lines show the evolution of the 580Y allele frequency under continued baseline ACT use. Red lines and blue lines show the evolution of the 580Y allele frequency after the deployment of ASMQ-PPQ and ALAQ, respectively.

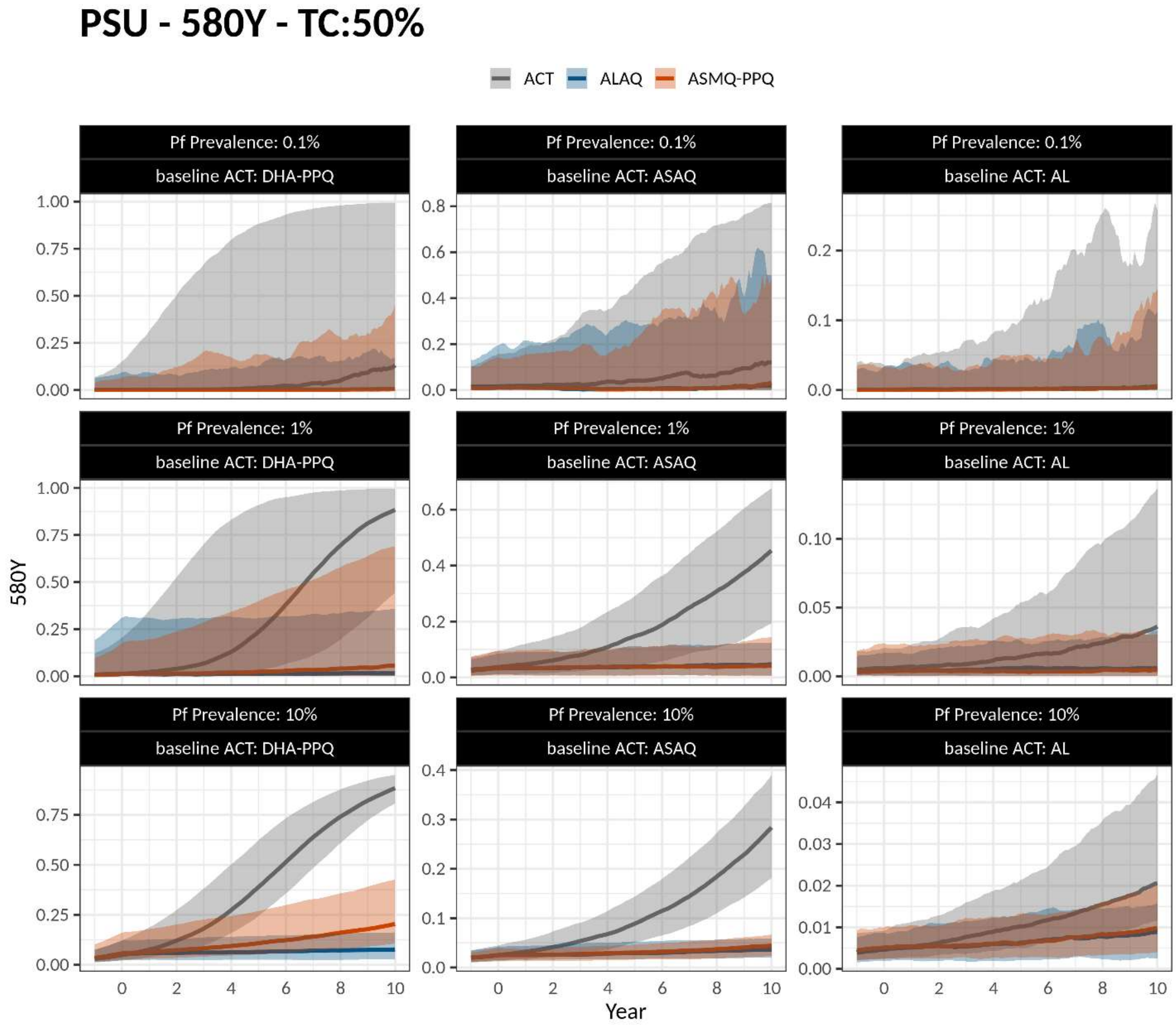

**Supplementary Fig. 6.** Simulations settings as in Supplementary Fig. 2. Figure title shows model used, outcome tracked, and treatment coverage (TC) level. Median trajectories are shown from 100 simulations, and the shaded area shows the range from the 5th to the 95th percentile. Grey lines show the evolution of the 580Y allele frequency under continued baseline ACT use. Red lines and blue lines show the evolution of the 580Y allele frequency after the deployment of ASMQ-PPQ and ALAQ, respectively.

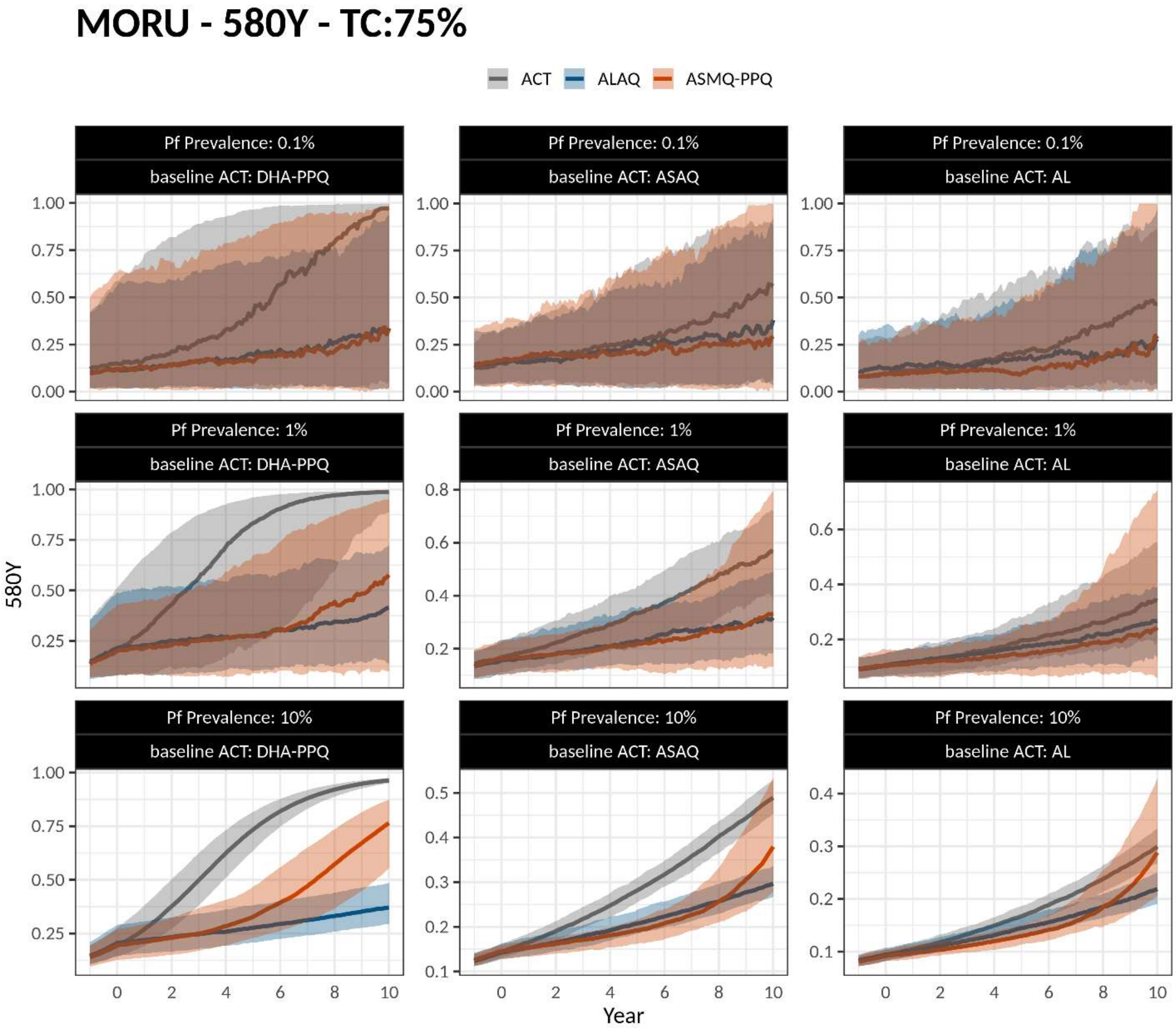

**Supplementary Fig. 7.** Simulations settings as in Supplementary Fig. 2. Figure title shows model used, outcome tracked, and treatment coverage (TC) level. Median trajectories are shown from 100 simulations, and the shaded area shows the range from the 5th to the 95th percentile. Grey lines show the evolution of the 580Y allele frequency under continued baseline ACT use. Red lines and blue lines show the evolution of the 580Y allele frequency after the deployment of ASMQ-PPQ and ALAQ, respectively.

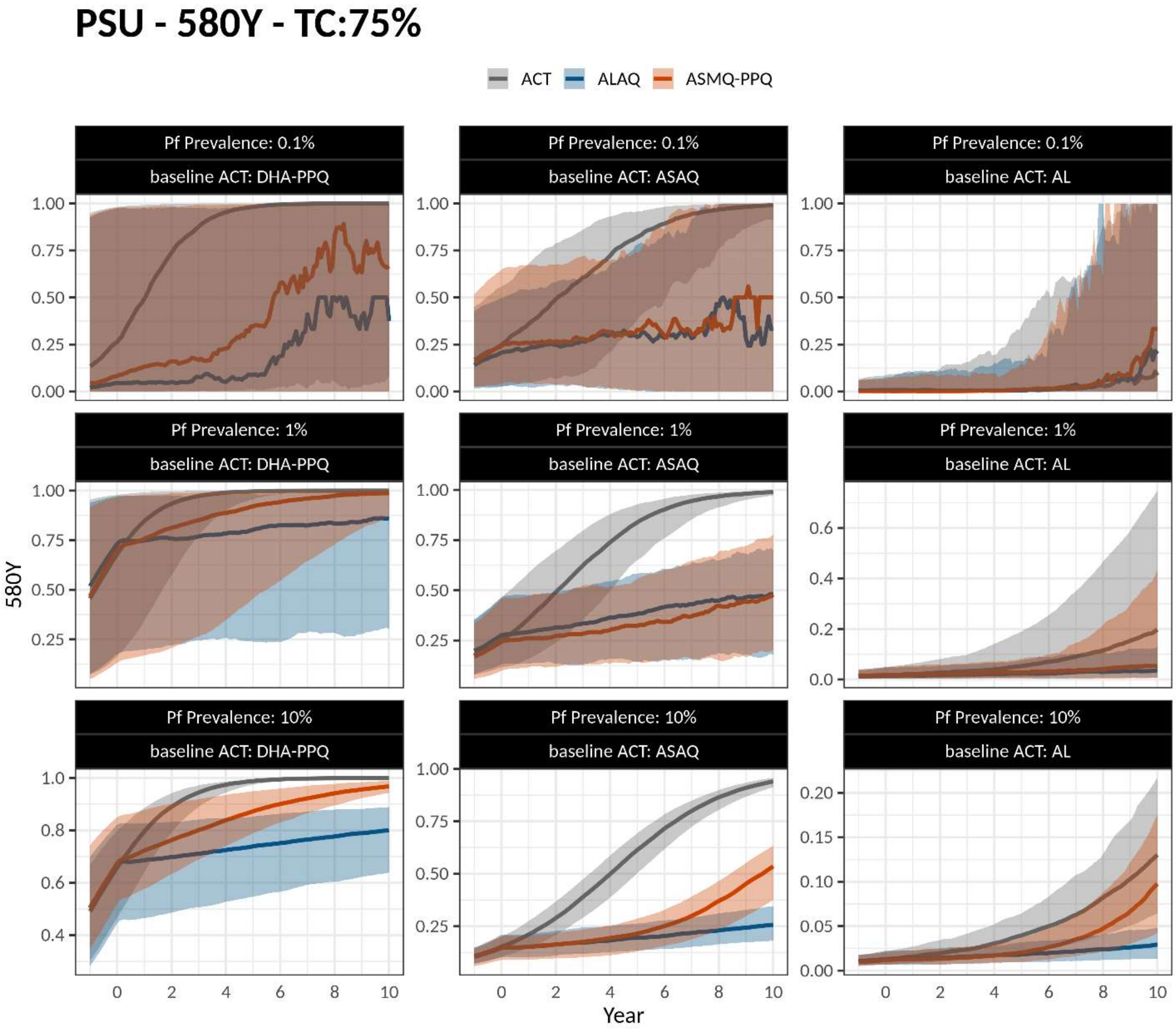

**Supplementary Fig. 8.** Simulations settings as in Supplementary Fig. 2. Figure title shows model used, outcome tracked, and treatment coverage (TC) level. Panels show monthly treatment failure rate (TFR) over 10 years. Median trajectories are shown from 100 simulations, and the shaded area shows the range from the 5th to the 95th percentile. Grey lines show the evolution of the 580Y allele frequency under continued baseline ACT use. Red lines and blue lines show the evolution of the 580Y allele frequency after the deployment of ASMQ-PPQ and ALAQ, respectively.

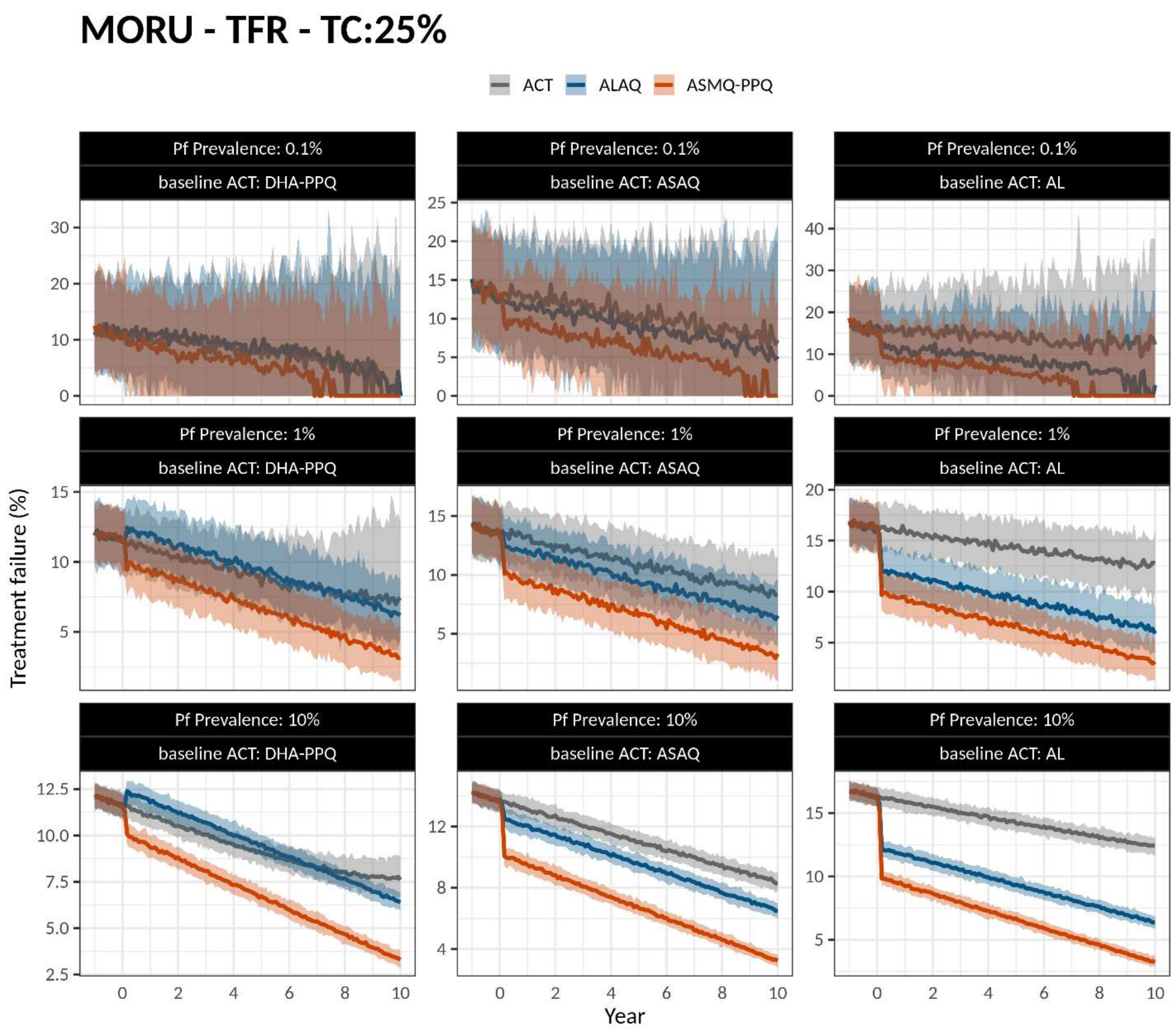

**Supplementary Fig. 9.** Simulations settings as in Supplementary Fig. 2. Figure title shows model used, outcome tracked, and treatment coverage (TC) level. Panels show monthly treatment failure rate (TFR) over 10 years. Median trajectories are shown from 100 simulations, and the shaded area shows the range from the 5th to the 95th percentile. Grey lines show the evolution of the 580Y allele frequency under continued baseline ACT use. Red lines and blue lines show the evolution of the 580Y allele frequency after the deployment of ASMQ-PPQ and ALAQ, respectively.

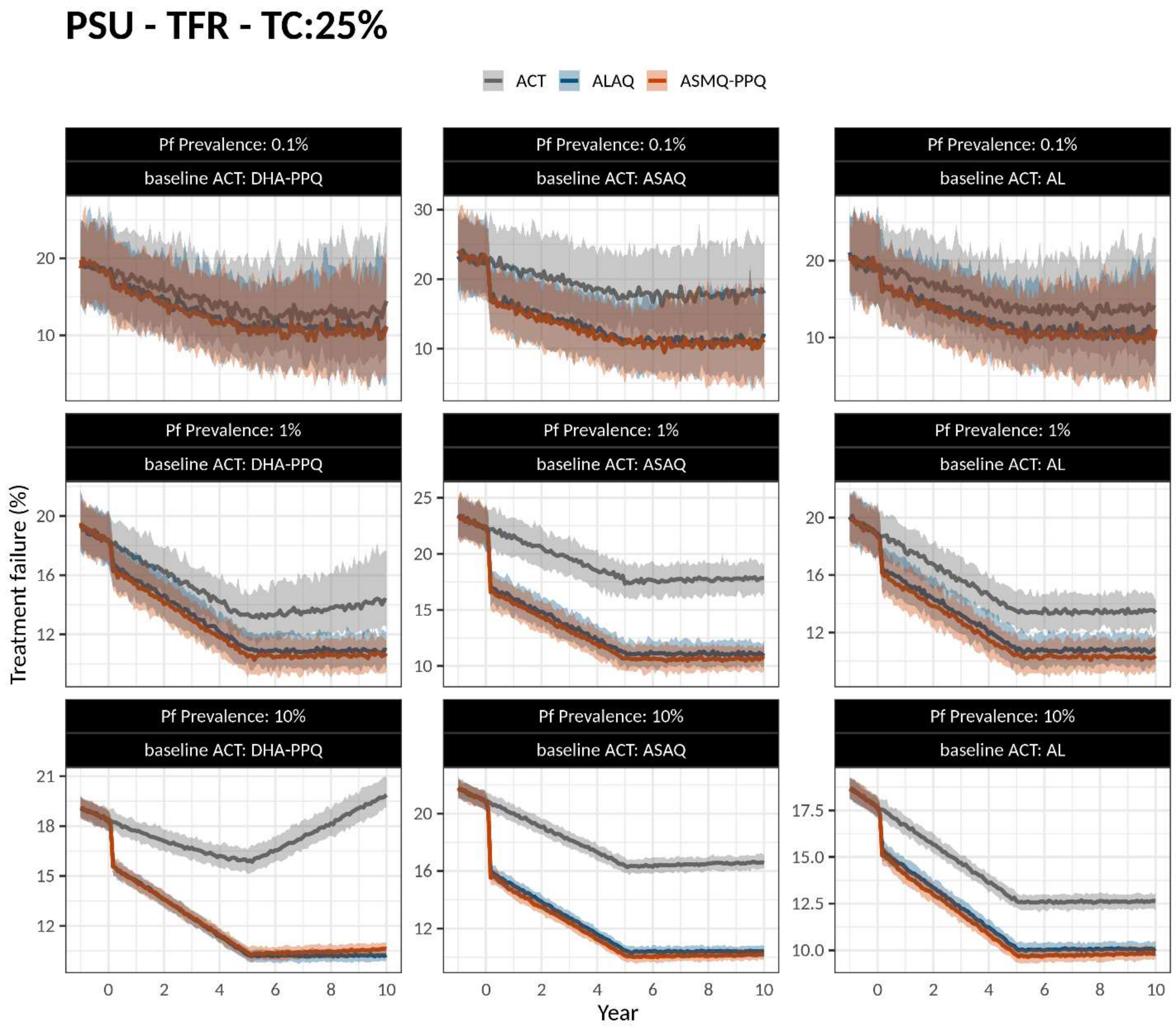

**Supplementary Fig. 10.** Simulations settings as in Supplementary Fig. 2. Figure title shows model used, outcome tracked, and treatment coverage (TC) level. Panels show monthly treatment failure rate (TFR) over 10 years. Median trajectories are shown from 100 simulations, and the shaded area shows the range from the 5th to the 95th percentile. Grey lines show the evolution of the 580Y allele frequency under continued baseline ACT use. Red lines and blue lines show the evolution of the 580Y allele frequency after the deployment of ASMQ-PPQ and ALAQ, respectively.

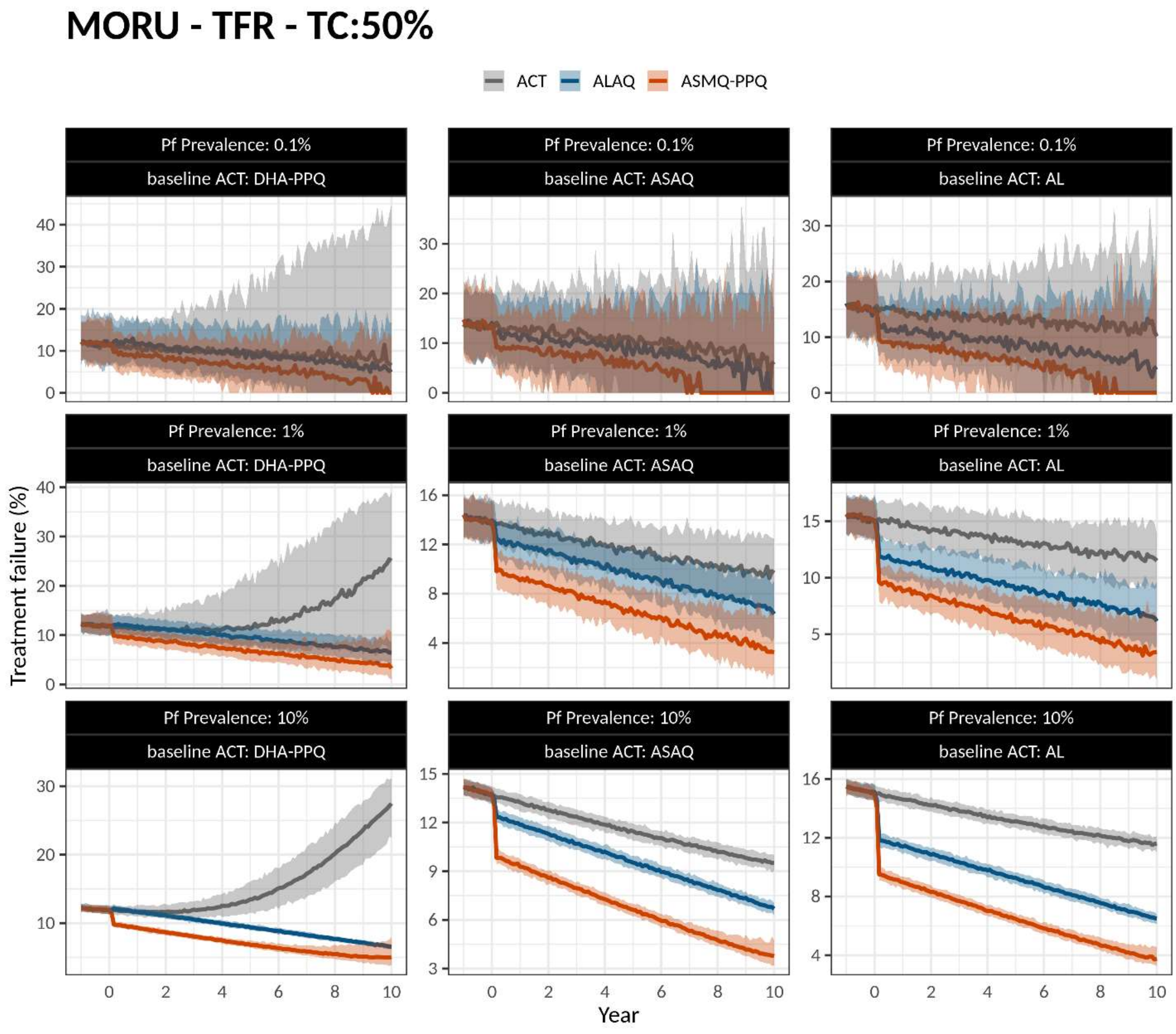

**Supplementary Fig. 11.** Simulations settings as in Supplementary Fig. 2. Figure title shows model used, outcome tracked, and treatment coverage (TC) level. Panels show monthly treatment failure rate (TFR) over 10 years. Median trajectories are shown from 100 simulations, and the shaded area shows the range from the 5$^{th}$ to the 95$^{th}$ percentile. Grey lines show the evolution of the 580Y allele frequency under continued baseline ACT use. Red lines and blue lines show the evolution of the 580Y allele frequency after the deployment of ASMQ-PPQ and ALAQ, respectively.

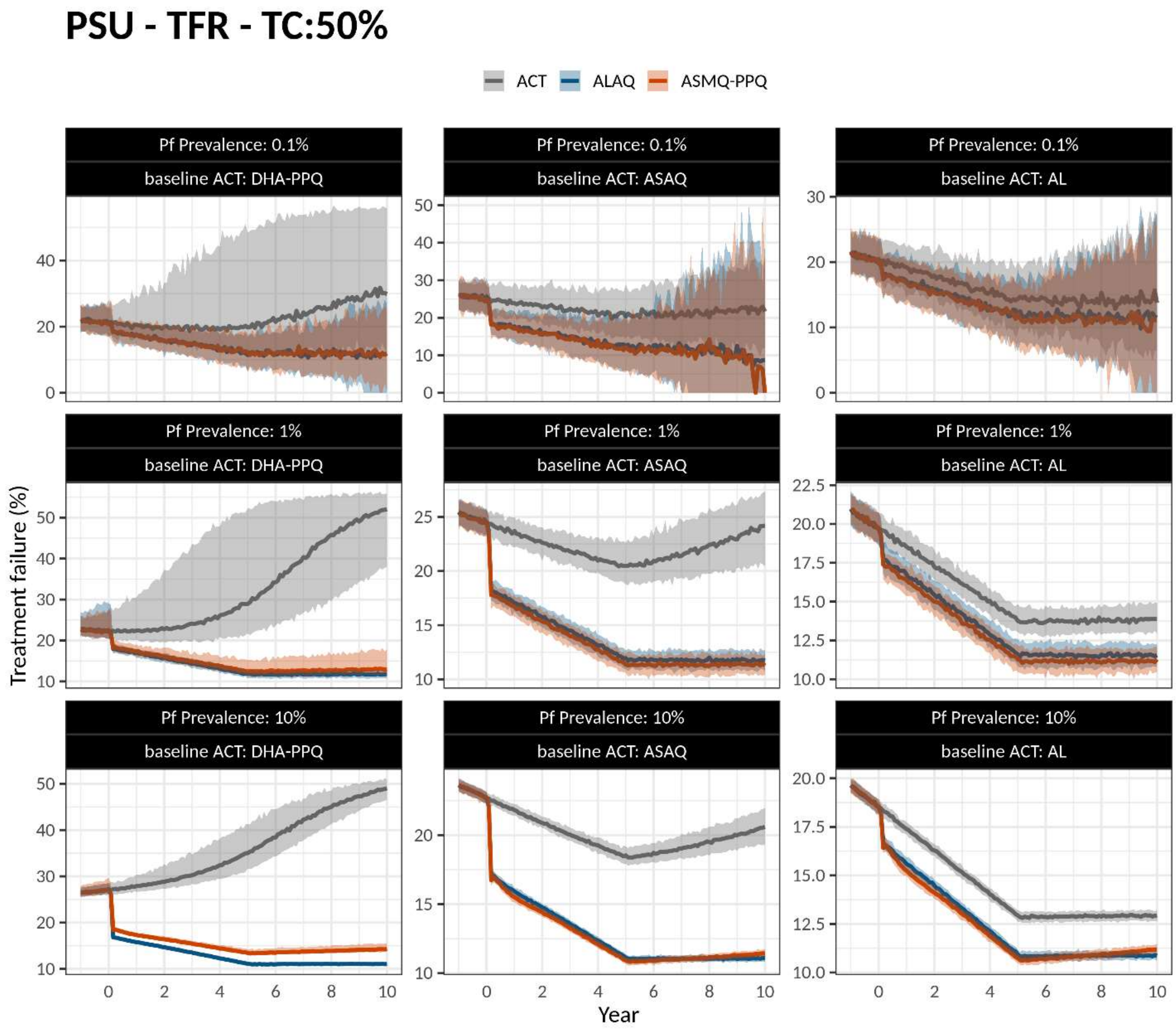

**Supplementary Fig. 12.** Simulations settings as in Supplementary Fig. 2. Figure title shows model used, outcome tracked, and treatment coverage (TC) level. Panels show monthly treatment failure rate (TFR) over 10 years. Median trajectories are shown from 100 simulations, and the shaded area shows the range from the 5th to the 95th percentile. Grey lines show the evolution of the 580Y allele frequency under continued baseline ACT use. Red lines and blue lines show the evolution of the 580Y allele frequency after the deployment of ASMQ-PPQ and ALAQ, respectively.

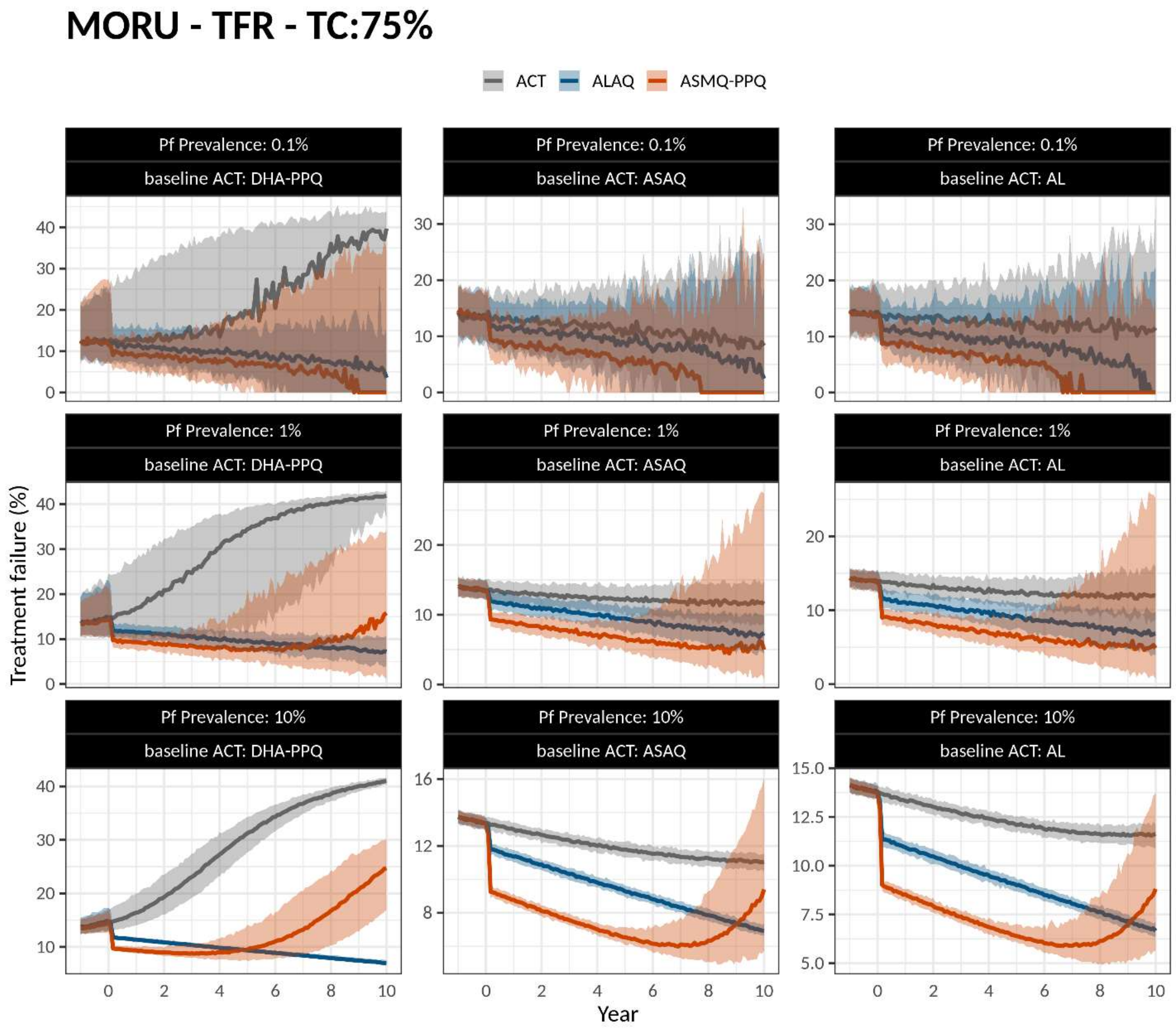

**Supplementary Fig. 13.** Simulations settings as in Supplementary Fig. 2. Figure title shows model used, outcome tracked, and treatment coverage (TC) level. Panels show monthly treatment failure rate (TFR) over 10 years. Median trajectories are shown from 100 simulations, and the shaded area shows the range from the 5th to the 95th percentile. Grey lines show the evolution of the 580Y allele frequency under continued baseline ACT use. Red lines and blue lines show the evolution of the 580Y allele frequency after the deployment of ASMQ-PPQ and ALAQ, respectively.

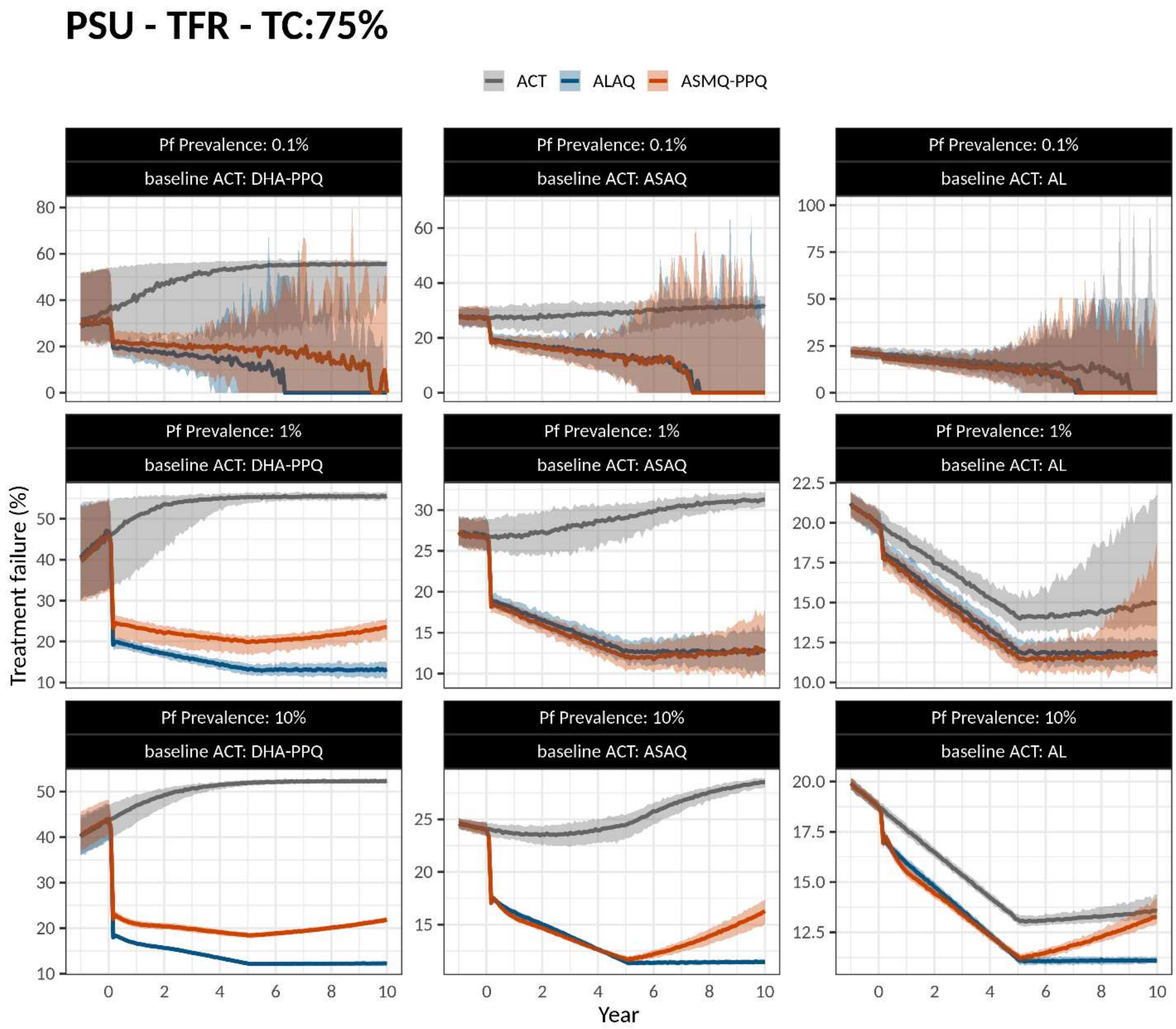

**Supplementary Fig. 14.** Simulations settings as in Supplementary Fig. 2. Figure title shows model used, outcome tracked, and treatment coverage (TC) level. Panels show all-age prevalence as measured by microscopy (*PfPR*) over 10 years. Median trajectories are shown from 100 simulations, and the shaded area shows the range from the 5th to the 95th percentile. Grey lines show the evolution of the 580Y allele frequency under continued baseline ACT use. Red lines and blue lines show the evolution of the 580Y allele frequency after the deployment of ASMQ-PPQ and ALAQ, respectively.

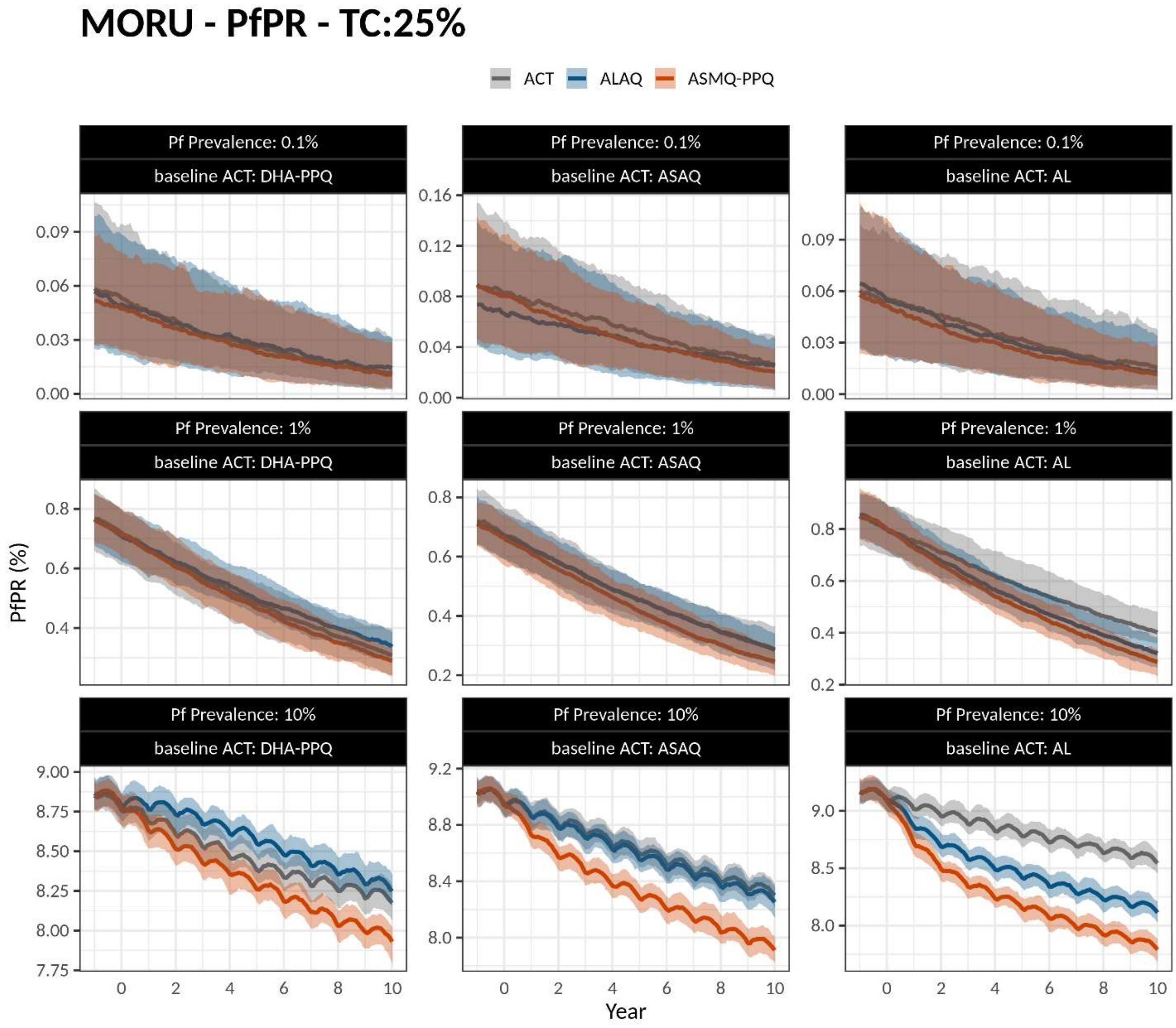

**Supplementary Fig. 15.** Simulations settings as in Supplementary Fig. 2. Figure title shows model used, outcome tracked, and treatment coverage (TC) level. Panels show all-ages prevalence as measured by microscopy (*PfPR*) over 10 years. Median trajectories are shown from 100 simulations, and the shaded area shows the range from the 5th to the 95th percentile. Grey lines show the evolution of the 580Y allele frequency under continued baseline ACT use. Red lines and blue lines show the evolution of the 580Y allele frequency after the deployment of ASMQ-PPQ and ALAQ, respectively.

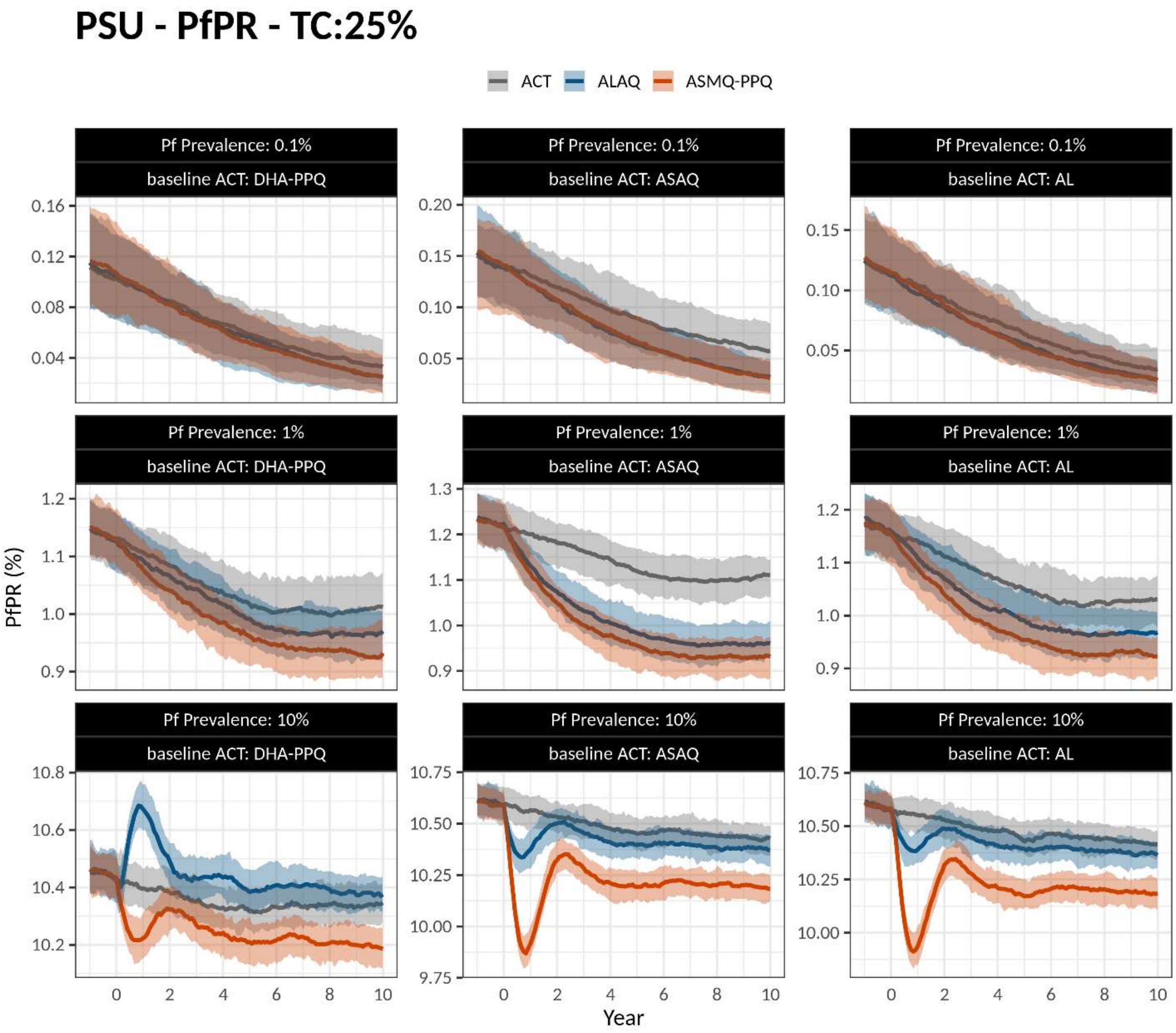

**Supplementary Fig. 16.** Simulations settings as in Supplementary Fig. 2. Figure title shows model used, outcome tracked, and treatment coverage (TC) level. Panels show all-ages *PfPR* over 10 years. Median trajectories are shown from 100 simulations, and the shaded area shows the range from the 5th to the 95th percentile. Grey lines show the evolution of the 580Y allele frequency under continued baseline ACT use. Red lines and blue lines show the evolution of the 580Y allele frequency after the deployment of ASMQ-PPQ and ALAQ, respectively.

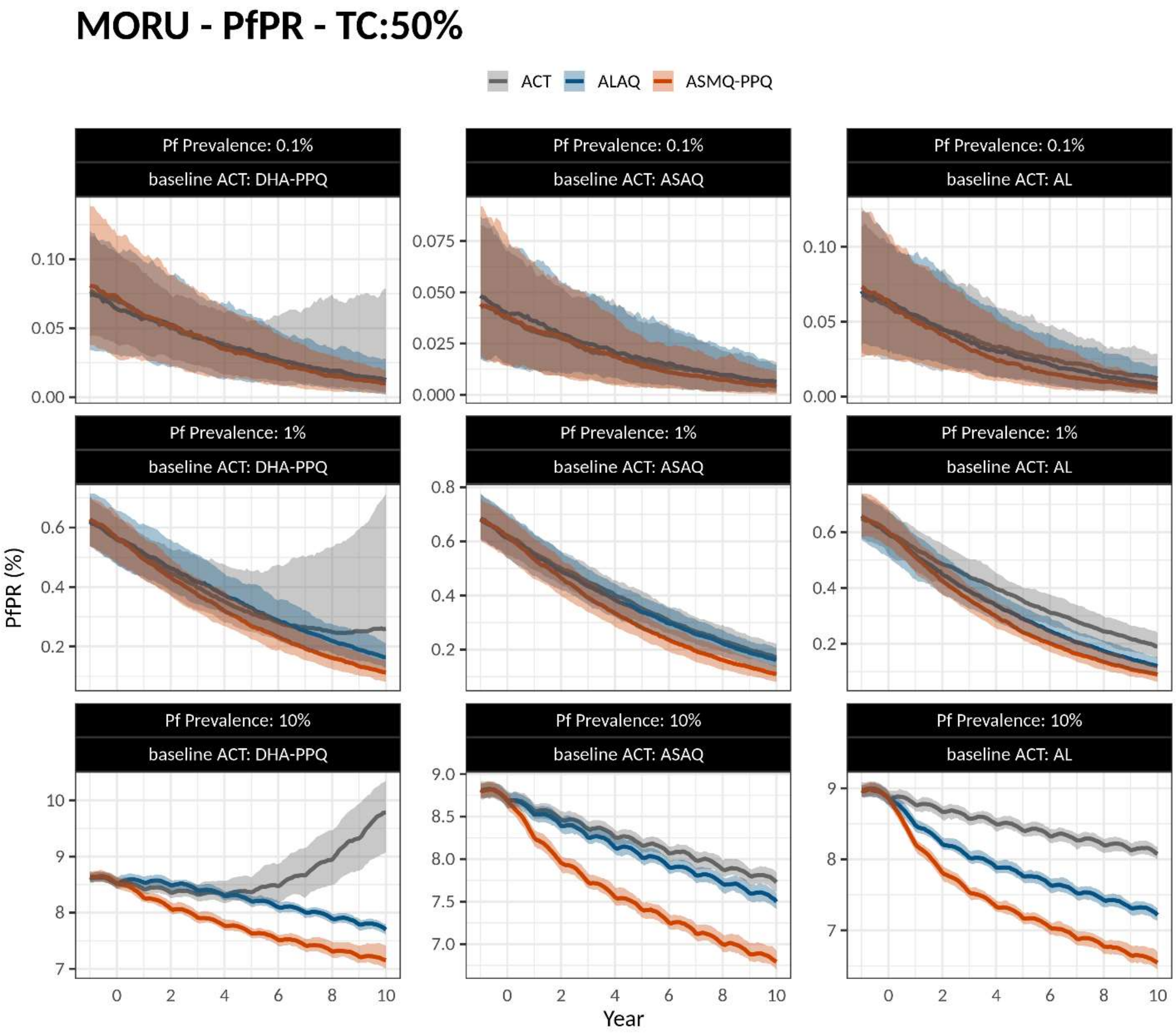

**Supplementary Fig. 17.** Simulations settings as in Supplementary Fig. 2. Figure title shows model used, outcome tracked, and treatment coverage (TC) level. Panels show all-ages *PfPR* over 10 years. Median trajectories are shown from 100 simulations, and the shaded area shows the range from the 5[th] to the 95[th] percentile. Grey lines show the evolution of the 580Y allele frequency under continued baseline ACT use. Red lines and blue lines show the evolution of the 580Y allele frequency after the deployment of ASMQ-PPQ and ALAQ, respectively.

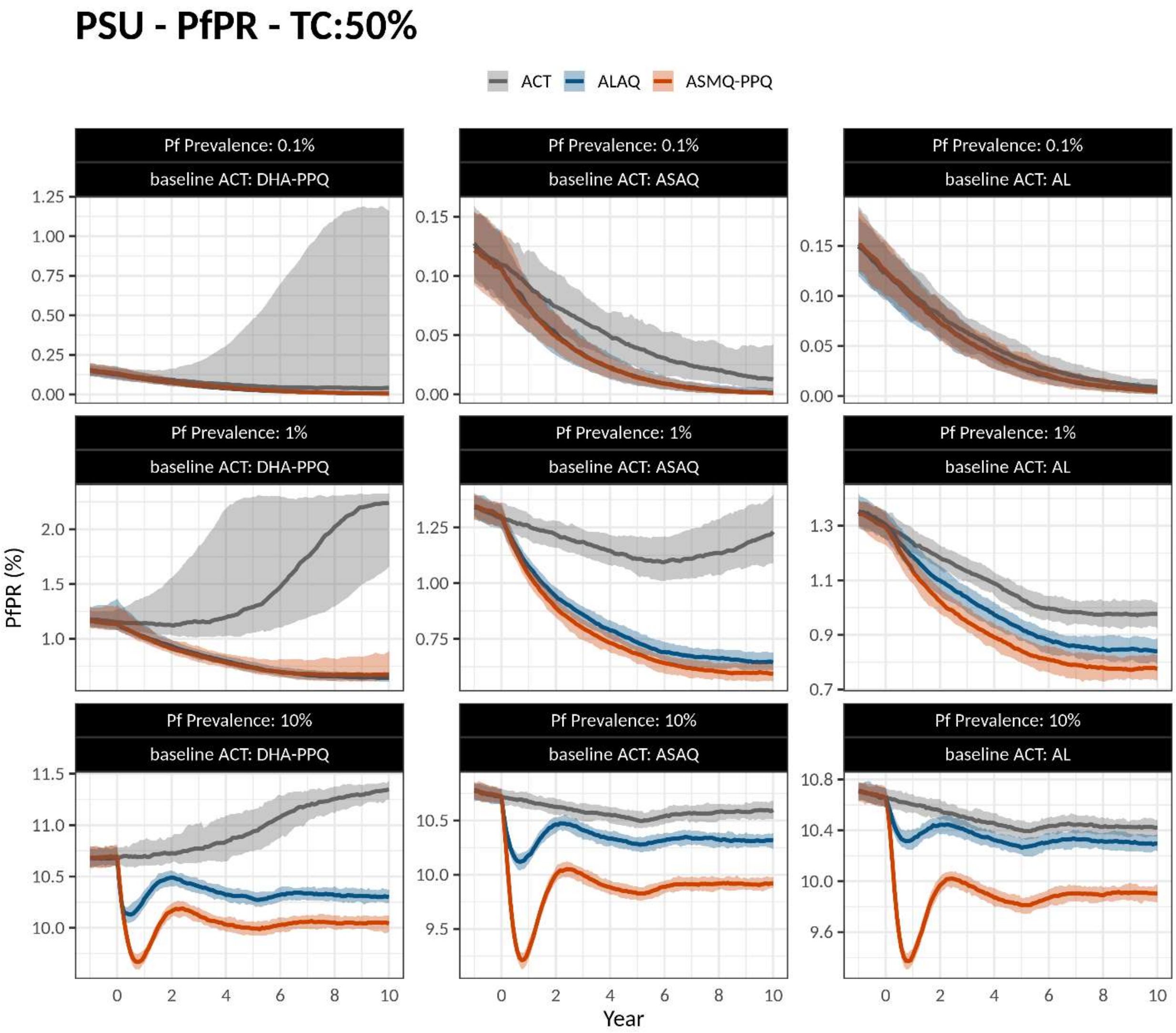

**Supplementary Fig. 18.** Simulations settings as in Supplementary Fig. 2. Figure title shows model used, outcome tracked, and treatment coverage (TC) level. Panels show all-ages *PfPR* over 10 years. Median trajectories are shown from 100 simulations, and the shaded area shows the range from the 5th to the 95th percentile. Grey lines show the evolution of the 580Y allele frequency under continued baseline ACT use. Red lines and blue lines show the evolution of the 580Y allele frequency after the deployment of ASMQ-PPQ and ALAQ, respectively.

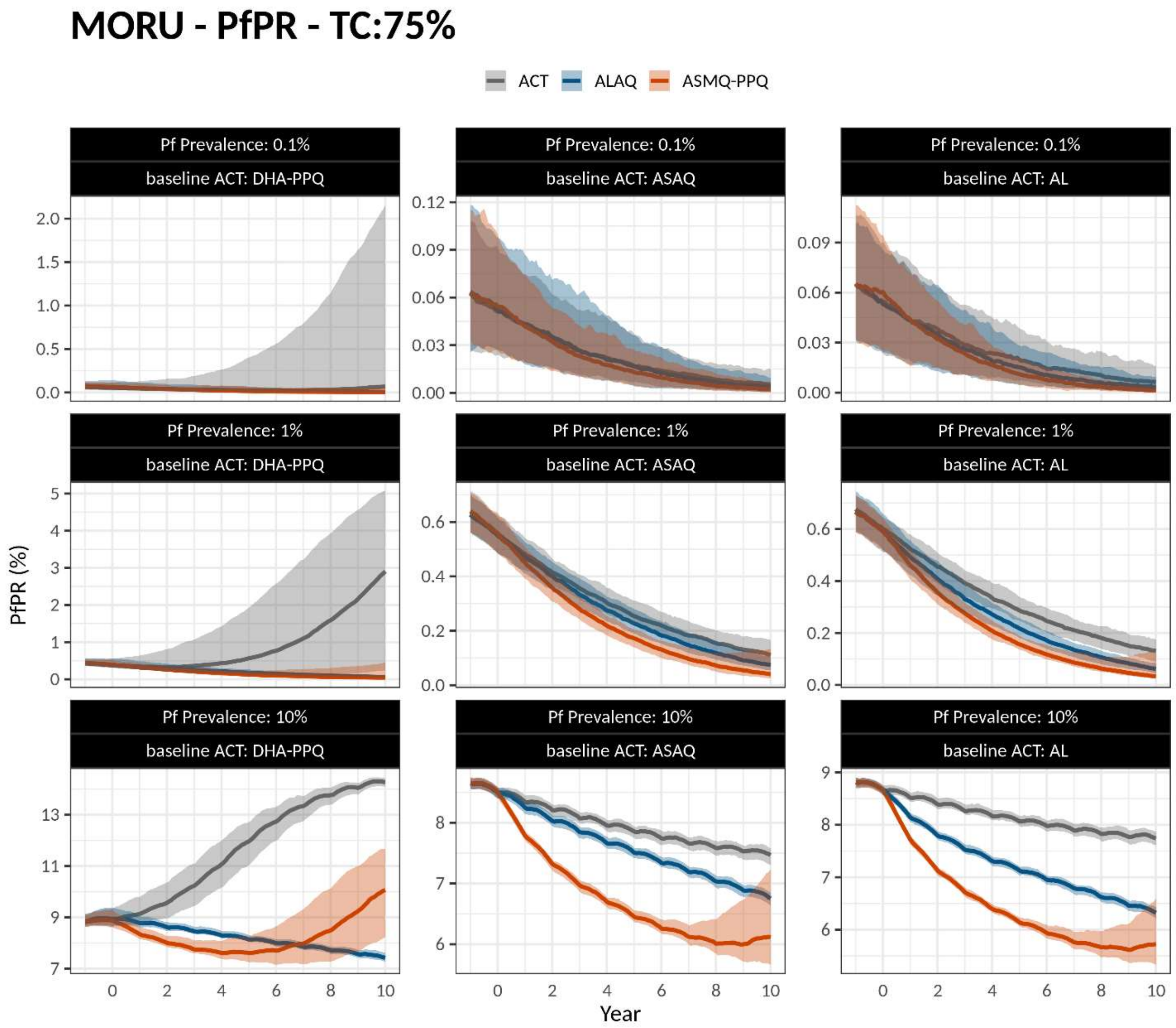

**Supplementary Fig. 19.** Simulations settings as in Supplementary Fig. 2. Figure title shows model used, outcome tracked, and treatment coverage (TC) level. Panels show all-ages *PfPR* over 10 years. Median trajectories are shown from 100 simulations, and the shaded area shows the range from the 5th to the 95th percentile. Grey lines show the evolution of the 580Y allele frequency under continued baseline ACT use. Red lines and blue lines show the evolution of the 580Y allele frequency after the deployment of ASMQ-PPQ and ALAQ, respectively.

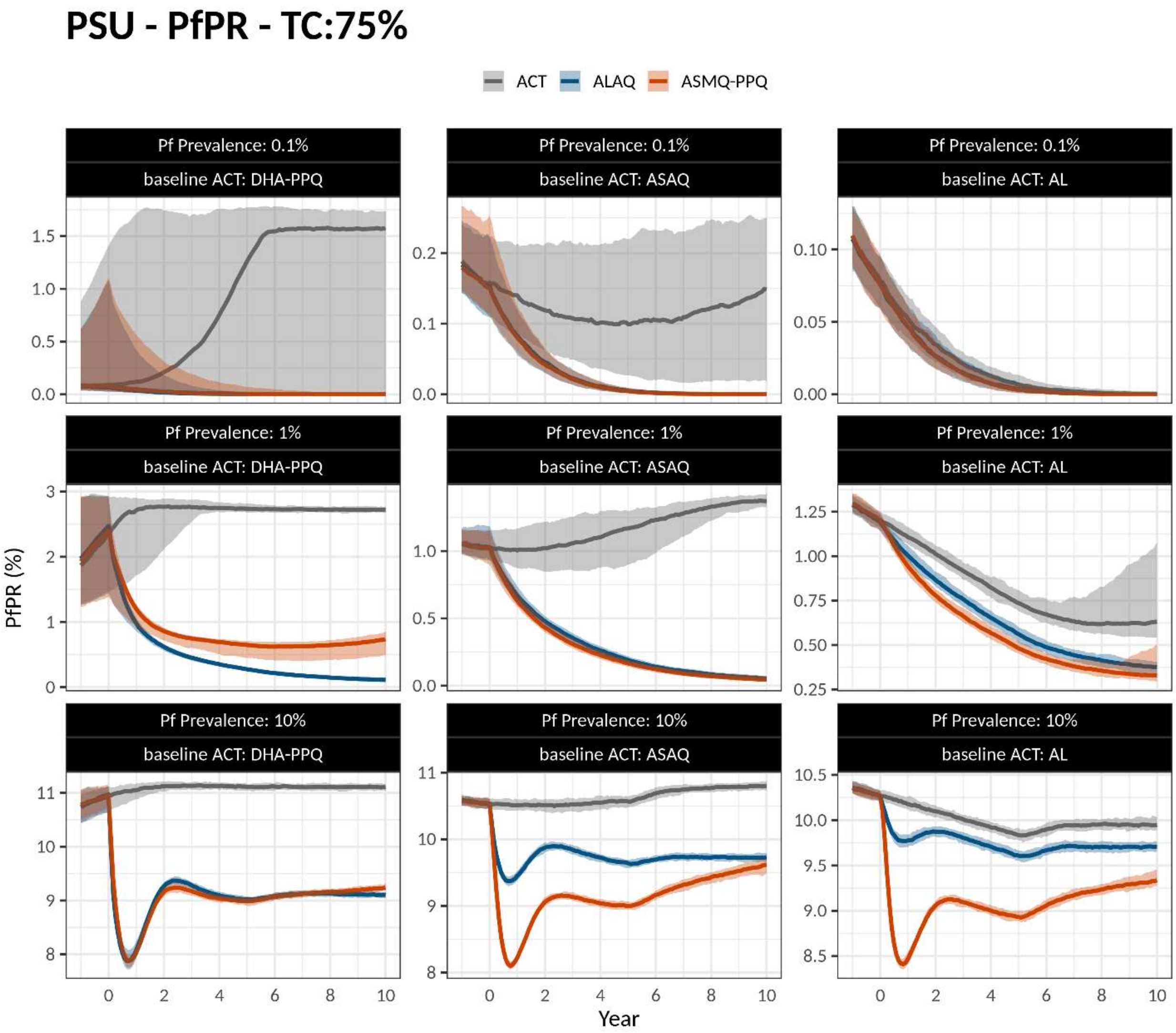

**Supplementary Fig. 20.** A total of 108 comparisons are done between a TACT outcome (here 580Y frequency at year 10) and continued ACT deployment: 3 different baseline ACTs of DHA-PPQ, ASAQ, AL; 3 different treatment coverages; 3 different transmission settings; 2 TACTs; and 2 models used. The Mann-Whitney U test was used to compare the 580Y frequency at year 10 between continued baseline ACTs use and ASMQ-PPQ use (left graph) or ALAQ use (right graph). Panels show the Mann-Whitney p-value for each comparison, and the colors show the results from the two models. All p-values lower than $10^{-6}$ are aggregated at the bottom-most tick on the graph.

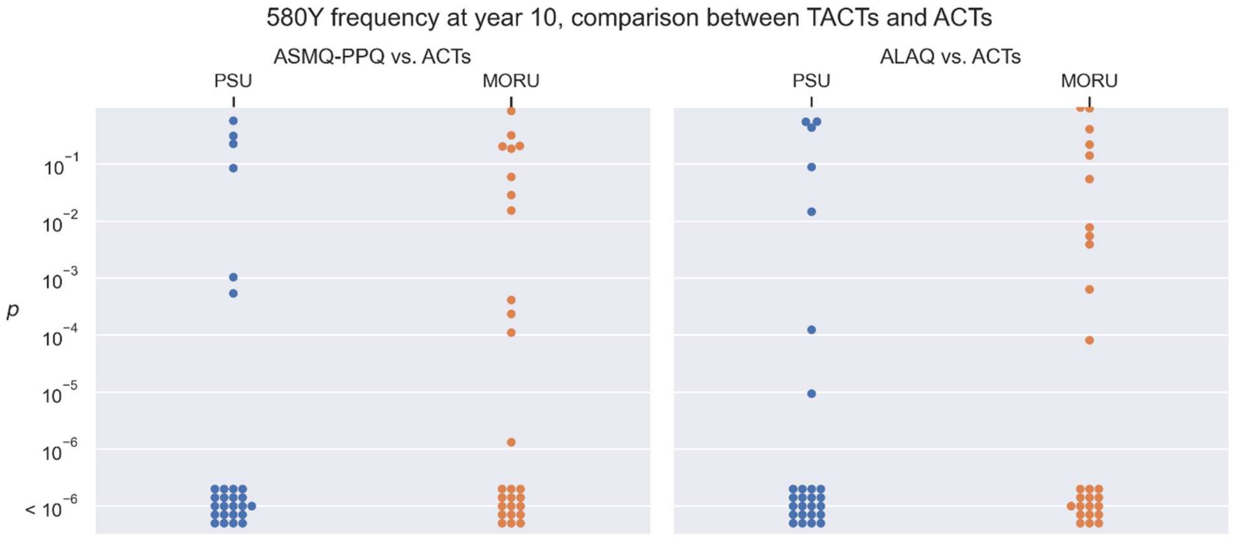

**Supplementary Fig. 21.** A total of 108 comparisons are done between a TACT outcome (here, the treatment failure rate at year 10) and continued ACT deployment: 3 different baseline ACTs of DHA-PPQ, ASAQ, AL; 3 different treatment coverages; 3 different transmission settings; 2 TACTs; and 2 models used. The Mann-Whitney U test was used to compare the 580Y frequency at year 10 between continued baseline ACTs use and ASMQ-PPQ use (left graph) or ALAQ use (right graph). Panels show the Mann-Whitney p-value for this comparison, and the colors show the results from the two models. All p-values lower than $10^{-6}$ are aggregated at the bottom-most tick on the graph.

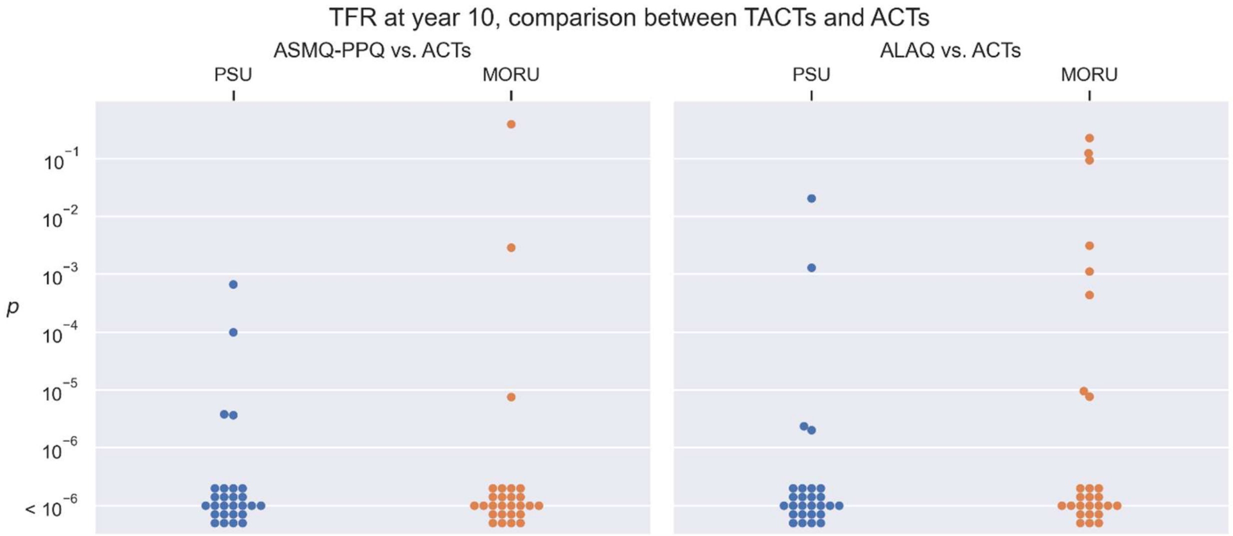

**Supplementary Fig. 22.** 580Y frequency 10 years after TACT deployment or continued ACT deployment, in three baseline scenarios of ACT use (columns) and three prevalence settings (rows). Treatment coverage is 25%; The leftmost pair of boxplots in each panel (in gray) show the 580Y frequency ten years later under a status quo ACT policy. The blue (ALAQ) and red (ASMQ-PPQ) boxplots show 580Y frequency outcomes after 10 years of a TACT policy. Boxplot pairs have MORU model results on the left and PSU model results on the right. All boxplots summarize 100 simulations.

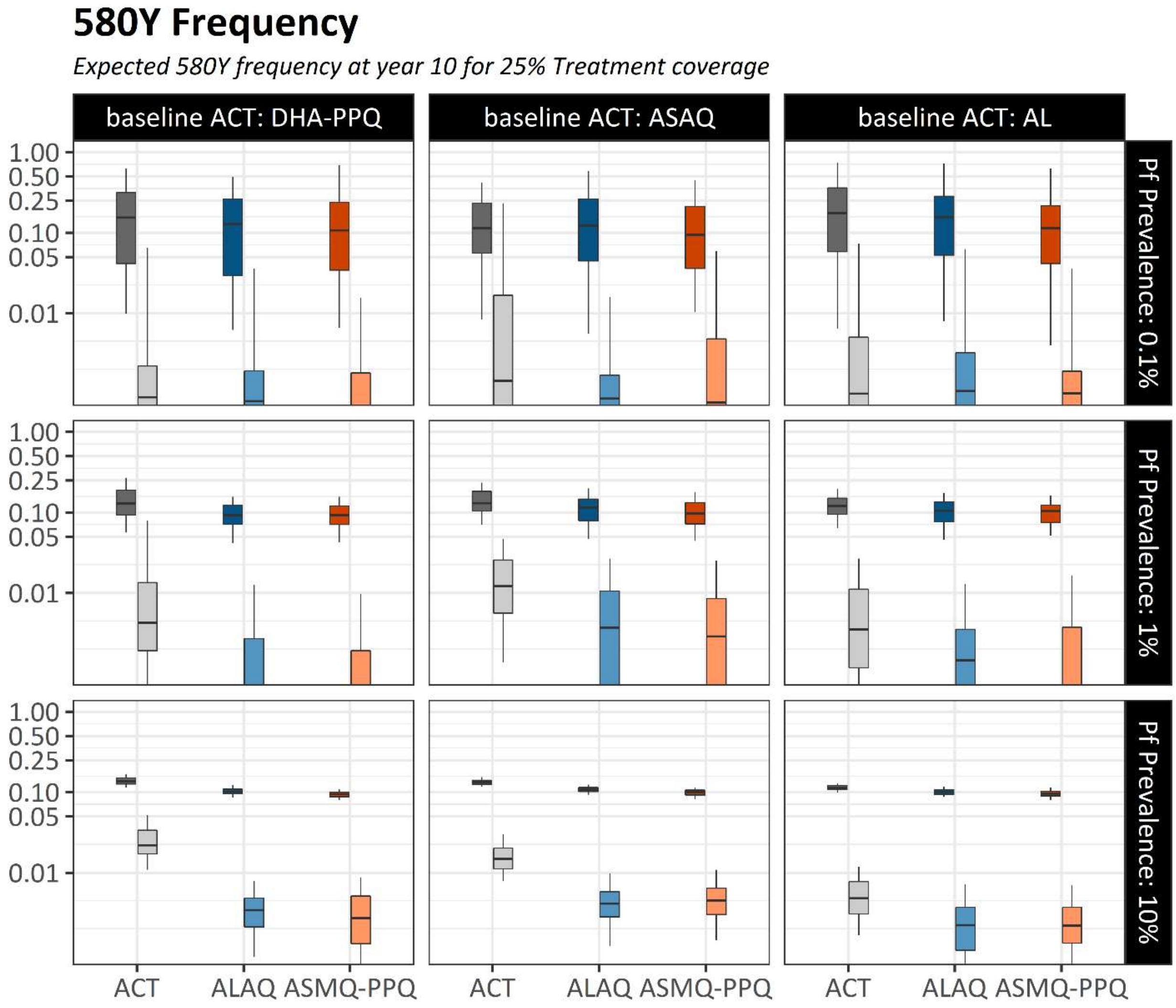

**Supplementary Fig. 23.** 580Y frequency 10 years after TACT deployment or continued ACT deployment, in three baseline scenarios of ACT use (columns) and three prevalence settings (rows). Treatment coverage is 75%; The leftmost pair of boxplots in each panel (in gray) show the 580Y frequency ten years later under a status quo ACT policy. The blue (ALAQ) and red (ASMQ-PPQ) boxplots show 580Y frequency outcomes after 10 years of a TACT policy. Boxplot pairs have MORU model results on the left and PSU model results on the right. All boxplots summarize 100 simulations.

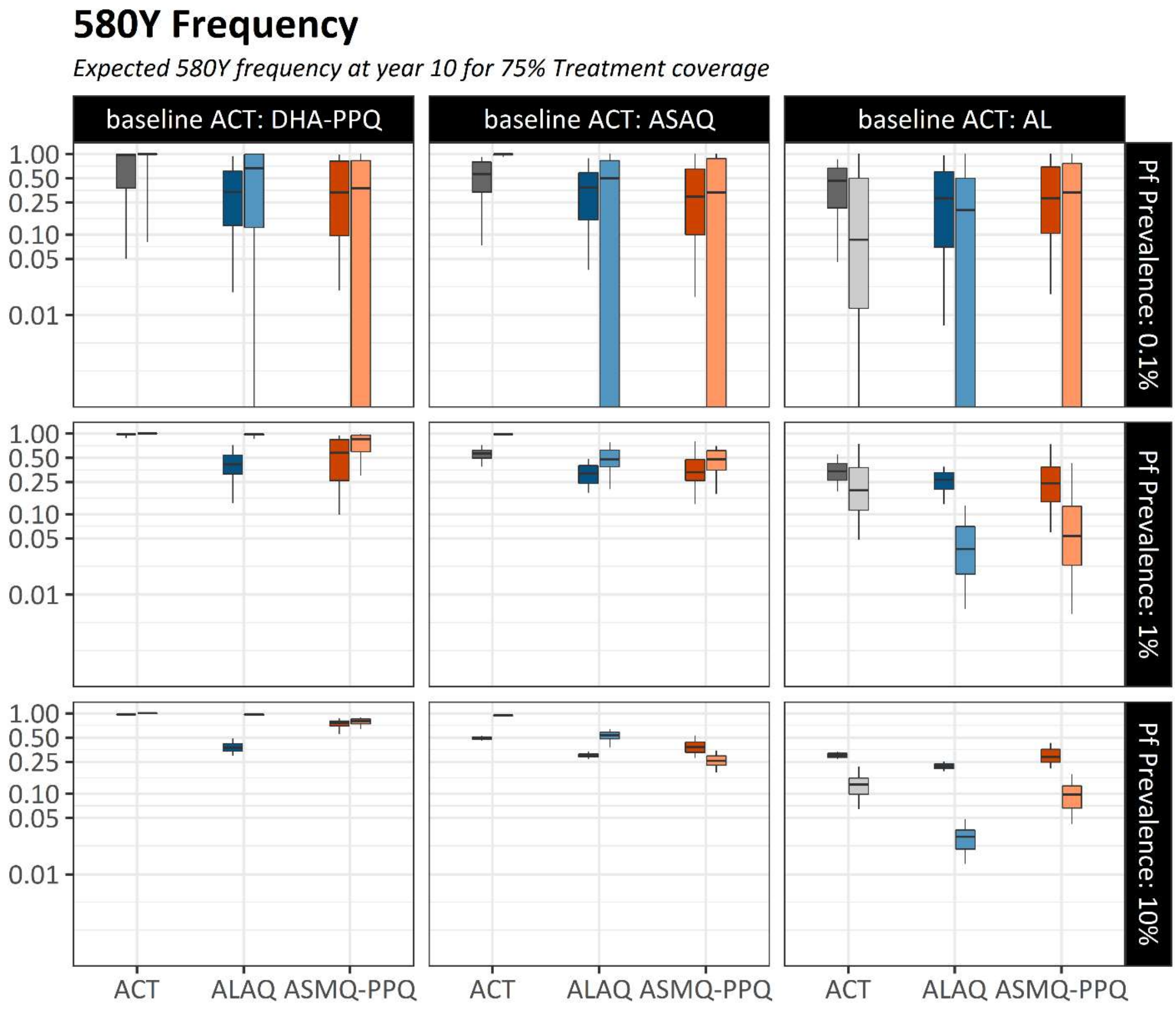

**Supplementary Fig. 24.** Treatment failure rates after 10 years of TACT deployment or ACT deployment, in three baseline scenarios of ACT use (columns) and three prevalence settings (rows). Treatment coverage is 25%; The leftmost pair of boxplots in each panel (in gray) show the TF rates ten years later under a status quo ACT policy. The blue (ALAQ) and red (ASMQ-PPQ) boxplots show treatment failure rate outcomes after 10 years of a TACT policy. Boxplot pairs have MORU model results on the left and PSU model results on the right. All boxplots summarize 100 simulations.

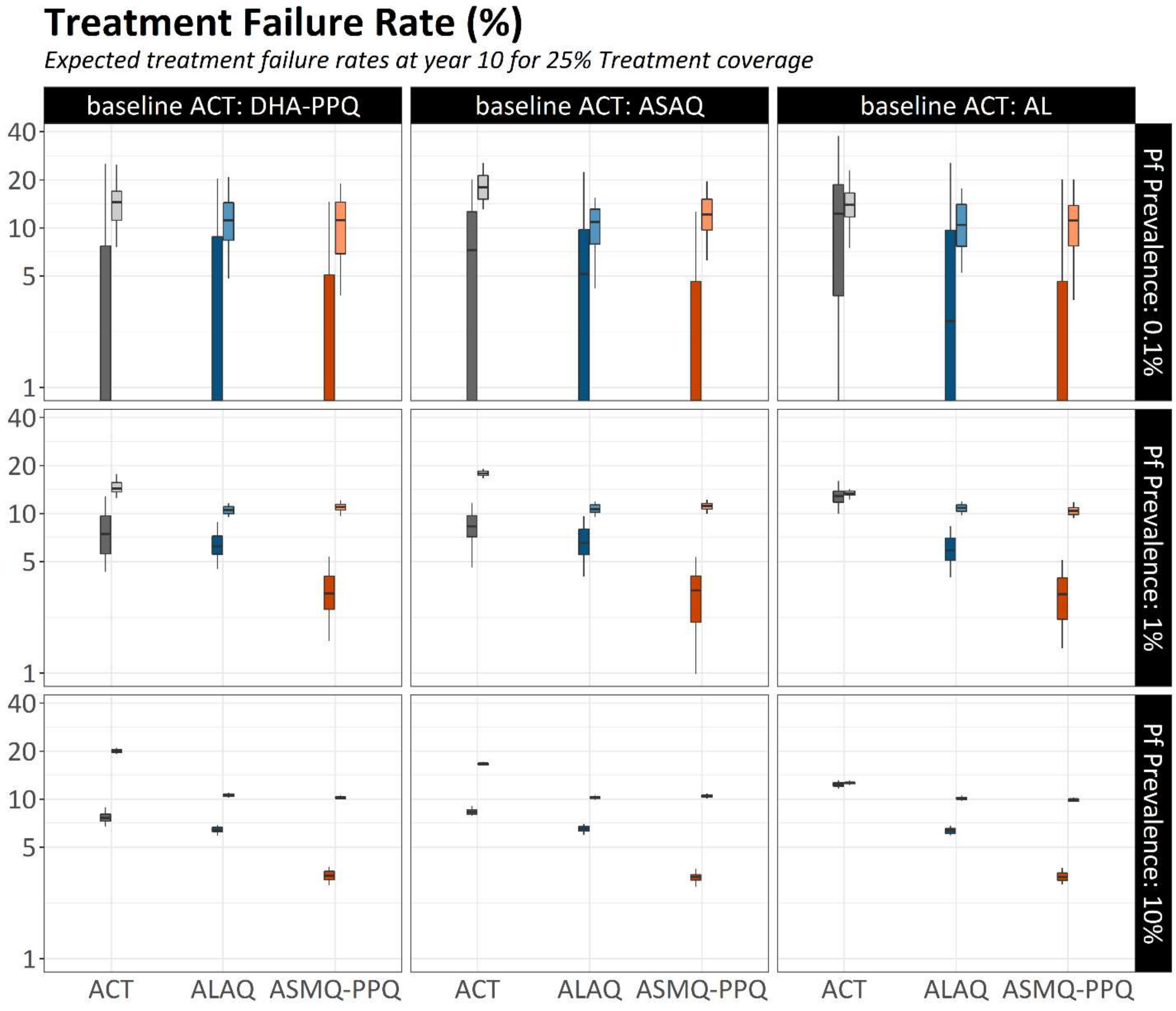

**Supplementary Fig. 25.** Treatment failure rates after 10 years of TACT deployment or ACT deployment, in three baseline scenarios of ACT use (columns) and three prevalence settings (rows). Treatment coverage is 75%; The leftmost pair of boxplots in each panel (in gray) show the TF rates ten years later under a status quo ACT policy. The blue (ALAQ) and red (ASMQ-PPQ) boxplots show treatment failure rate outcomes after 10 years of a TACT policy. Boxplot pairs have MORU model results on the left and PSU model results on the right. All boxplots summarize 100 simulations.

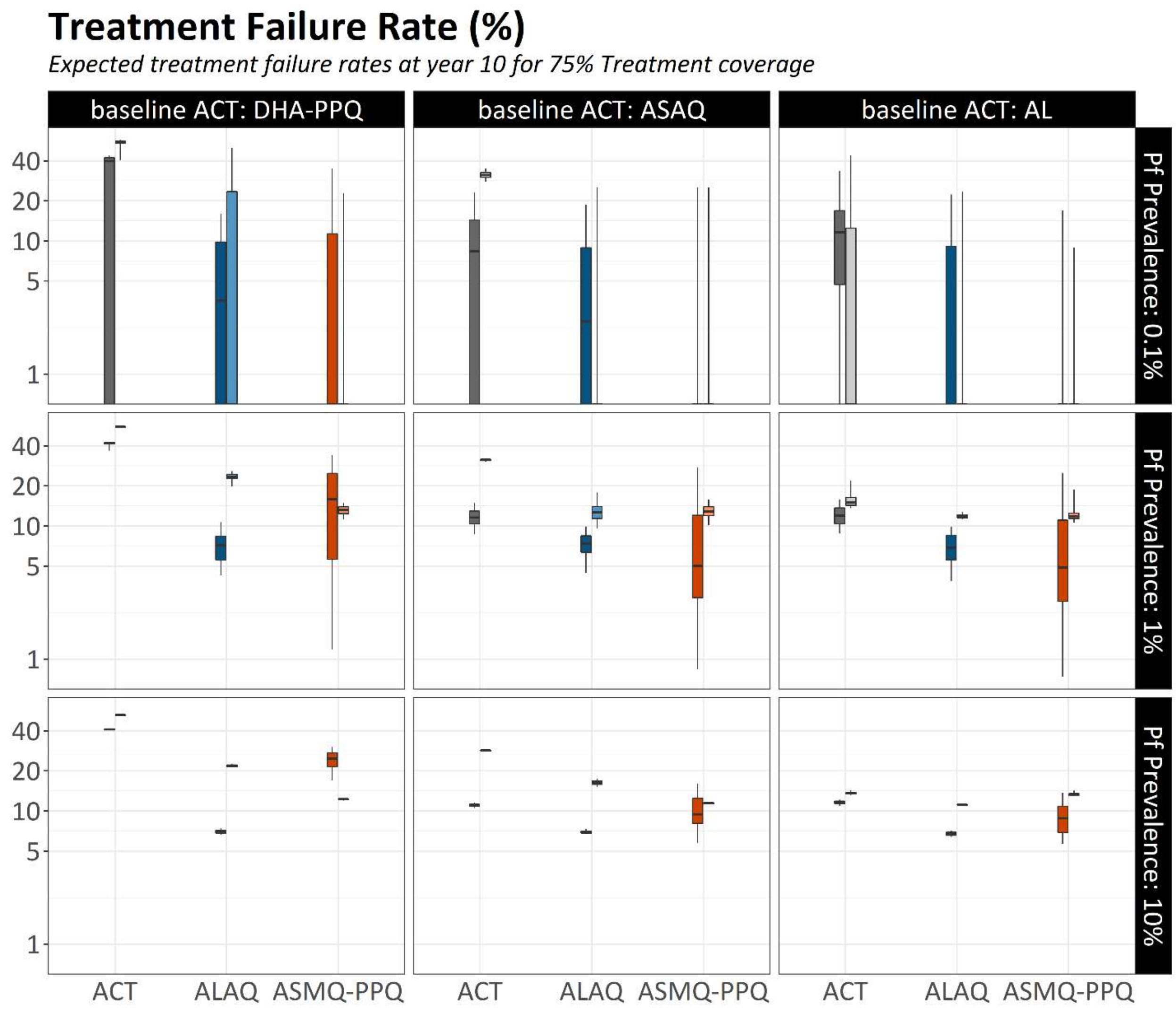

**Supplementary Fig. 26.** Pf prevalence after 10 years of TACT deployment or ACT deployment, in three baseline scenarios of ACT use (columns) and three prevalence settings (rows). Treatment coverage is 50%; The leftmost pair of boxplots in each panel (in gray) show Pf prevalence ten years later under a status quo ACT policy. The blue (ALAQ) and red (ASMQ-PPQ) boxplots show prevalence outcomes after 10 years of a TACT policy. Boxplot pairs have MORU model results on the left and PSU model results on the right. All boxplots summarize 100 simulations. The percent reduction in median Pf prevalence from ACT to TACT is shown next to the median of each TACT boxplot.

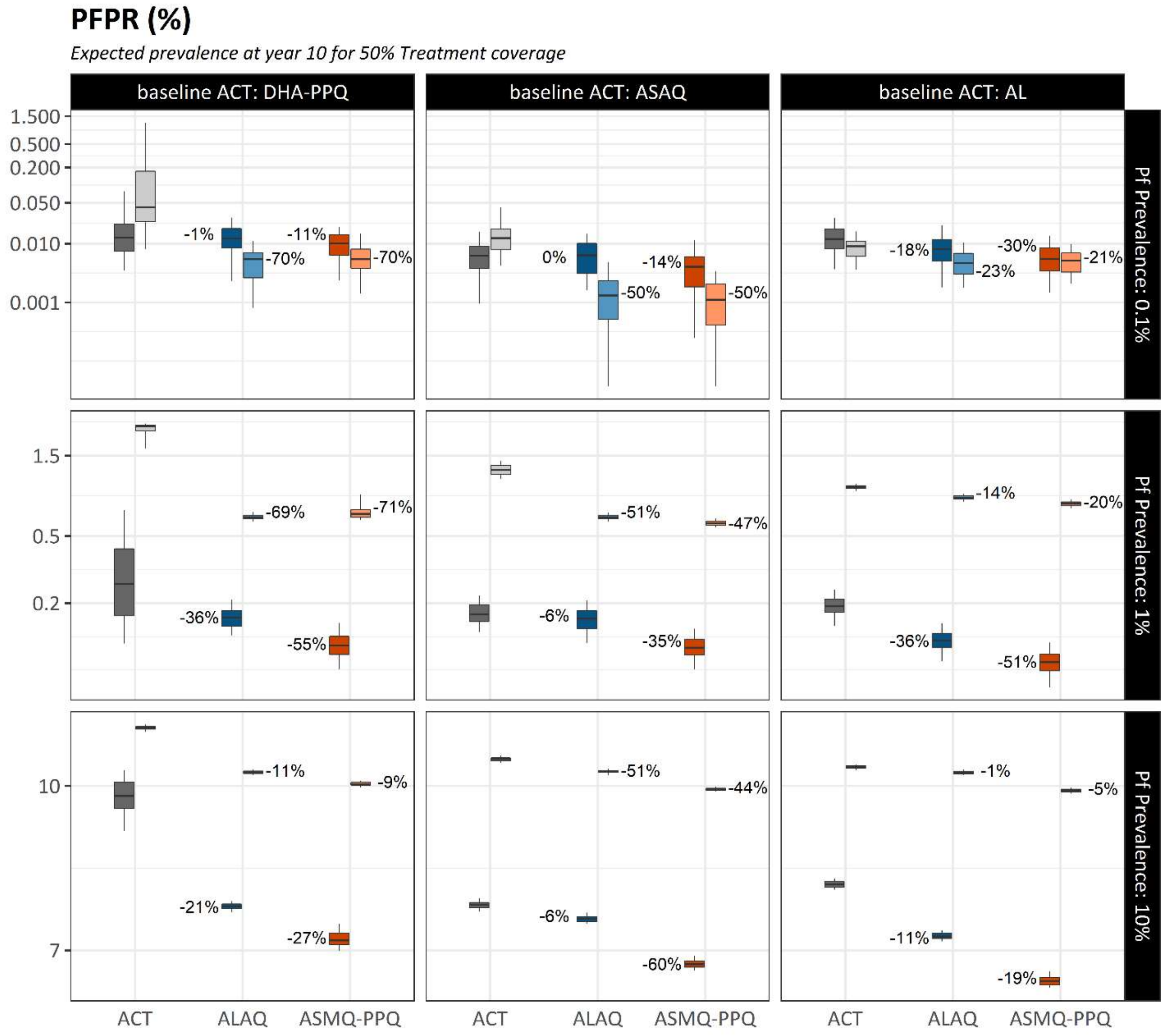

**Supplementary Fig. 27.** Pf prevalence after 10 years of TACT deployment or ACT deployment, in three baseline scenarios of ACT use (columns) and three prevalence settings (rows). Treatment coverage is 25%; The leftmost pair of boxplots in each panel (in gray) show Pf prevalence ten years later under a status quo ACT policy. The blue (ALAQ) and red (ASMQ-PPQ) boxplots show prevalence outcomes after 10 years of a TACT policy. Boxplot pairs have MORU model results on the left and PSU model results on the right. All boxplots summarize 100 simulations.

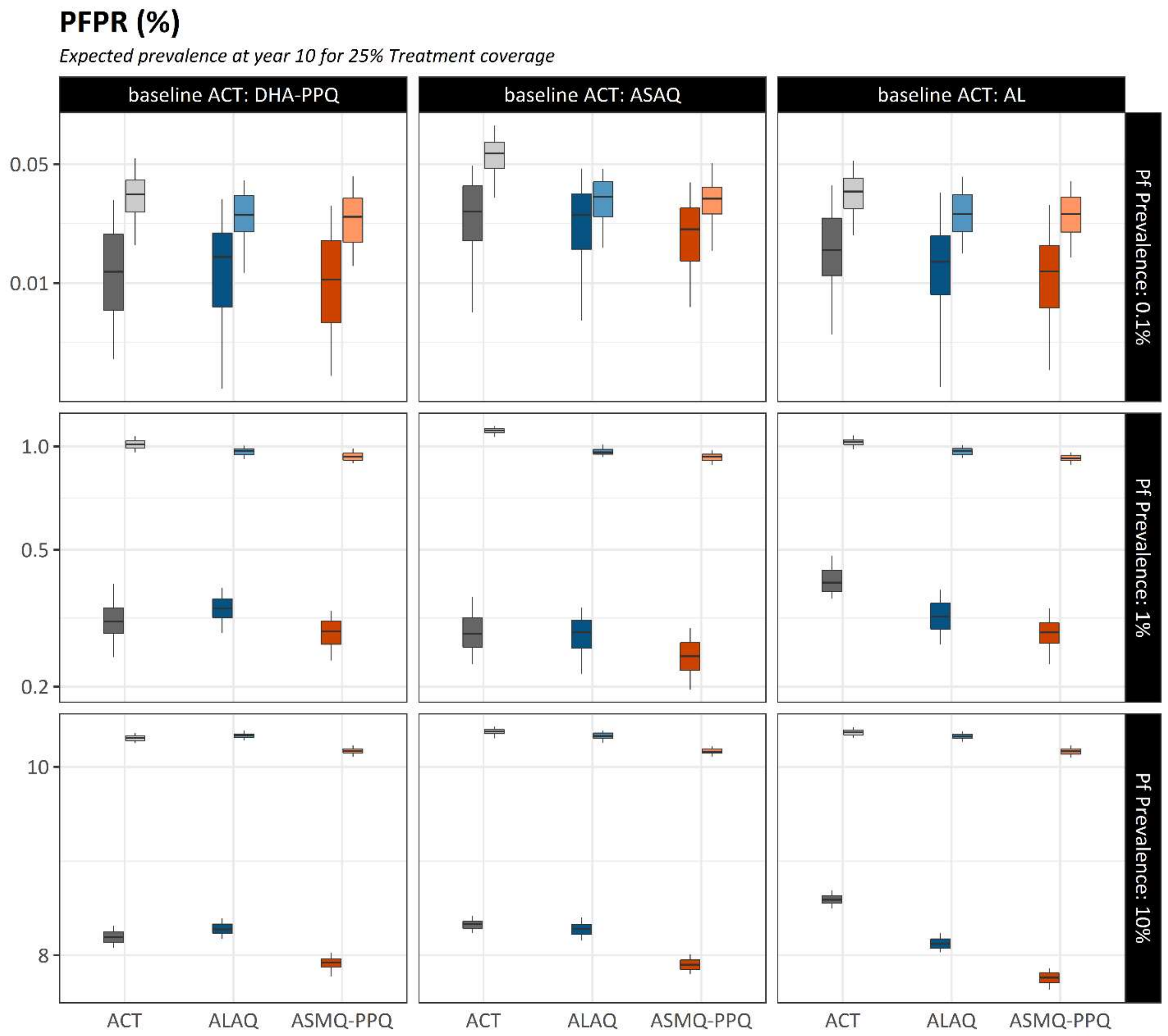

**Supplementary Fig. 28.** Pf prevalence after 10 years of TACT deployment or ACT deployment, in three baseline scenarios of ACT use (columns) and three prevalence settings (rows). Treatment coverage is 75%; The leftmost pair of boxplots in each panel (in gray) show Pf prevalence ten years later under a status quo ACT policy. The blue (ALAQ) and red (ASMQ-PPQ) boxplots show prevalence outcomes after 10 years of a TACT policy. Boxplot pairs have MORU model results on the left and PSU model results on the right. All boxplots summarize 100 simulations.

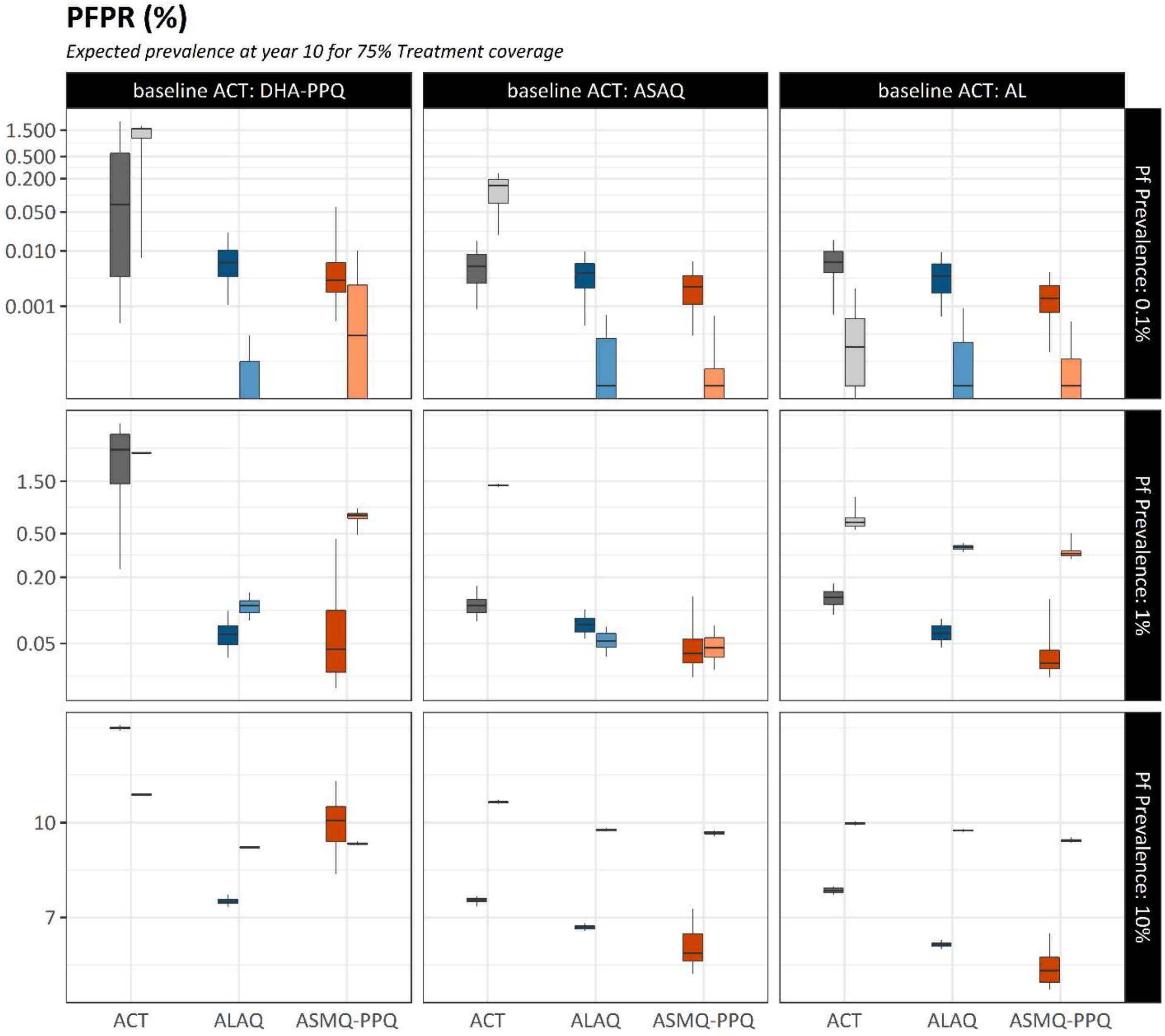

**Supplementary Fig. 29. Expected 580Y frequency dynamics for continued ACT use.** Each line represents the median 580Y frequency over a 10-year period with continued use of the ACT regimen identified by each column in a setting with Pf prevalence identified in each row. Shaded areas are bounded by the 25th and 75th percentiles obtained from 100 simulations for each scenario. Treatment coverage is set to 50%.

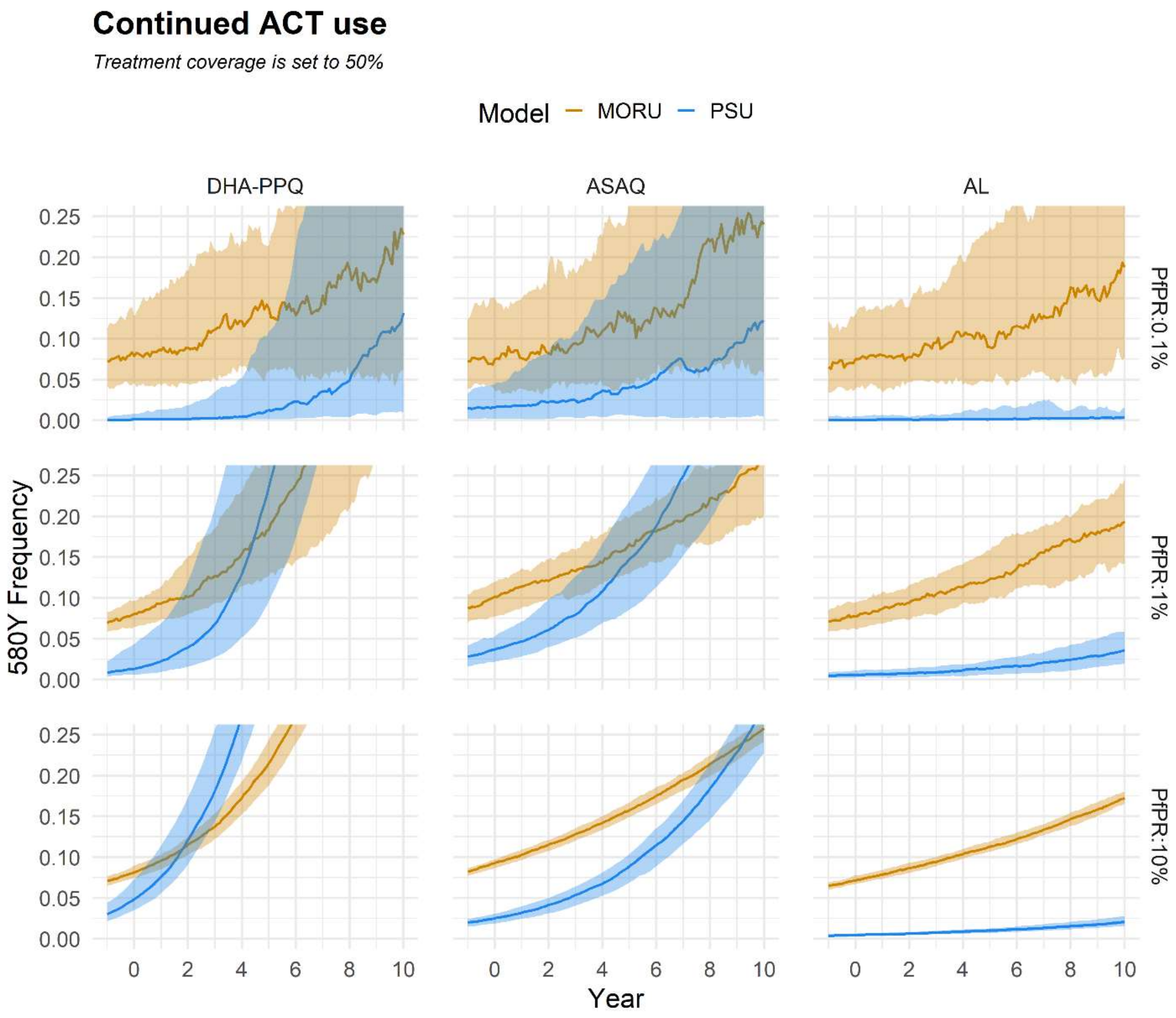

**Supplementary Fig. 30. Expected 580Y frequency dynamics for continued ACT use.** Each line represents the median 580Y frequency over a 10-year period with continued use of the ACT regimen identified by each column in a setting with Pf prevalence identified in each row. Shaded areas are bounded by the 25$^{th}$ and 75$^{th}$ percentiles obtained from 100 simulations for each scenario. Treatment coverage is set to 25%.

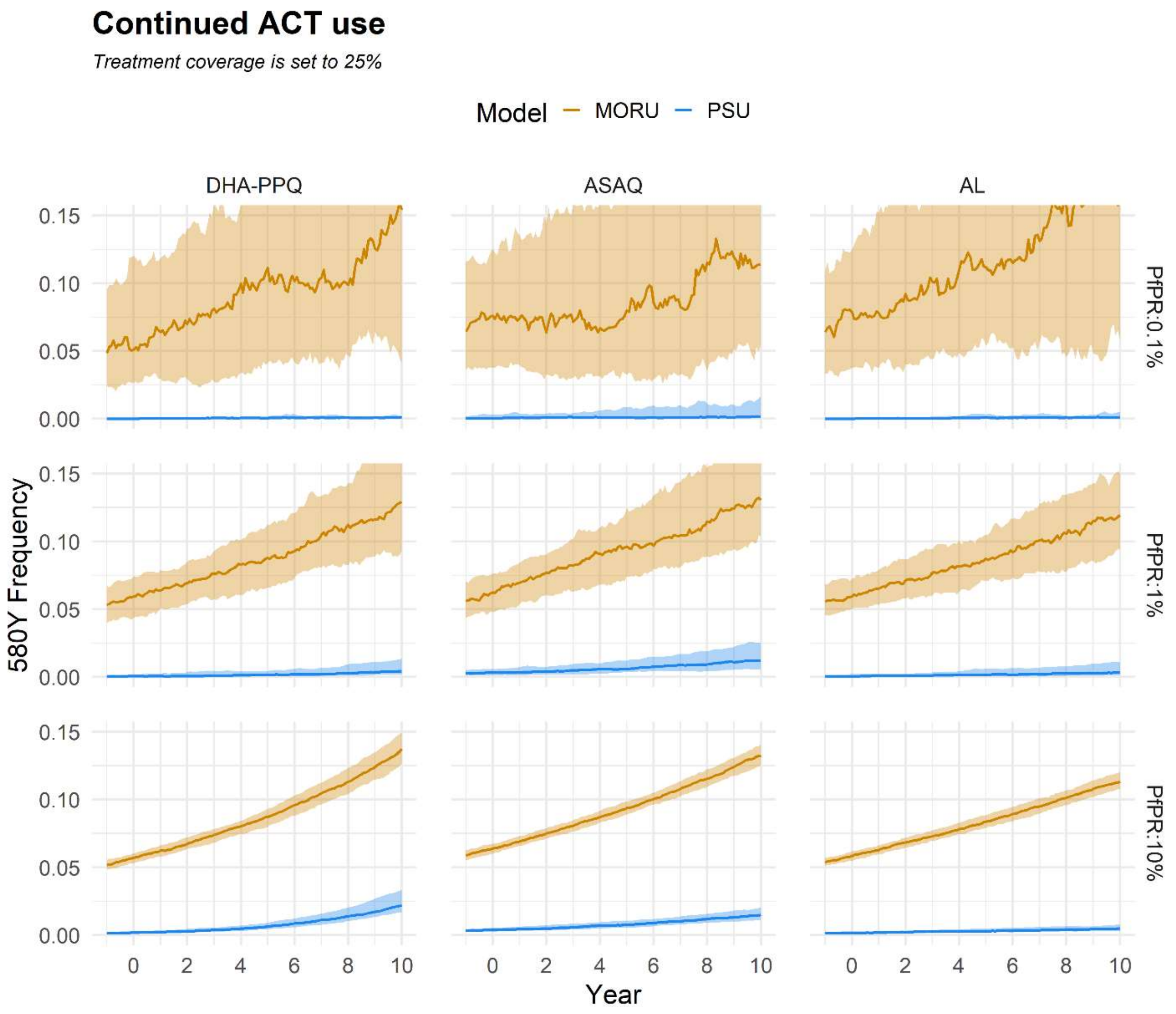

**Supplementary Fig. 31. Expected 580Y frequency dynamics for continued ACT use.** Each line represents the median 580Y frequency over a 10-year period with continued use of the ACT regimen identified by each column in a setting with Pf prevalence identified in each row. Shaded areas are bounded by the 25th and 75th percentiles obtained from 100 simulations for each scenario. Treatment coverage is set to 75%.

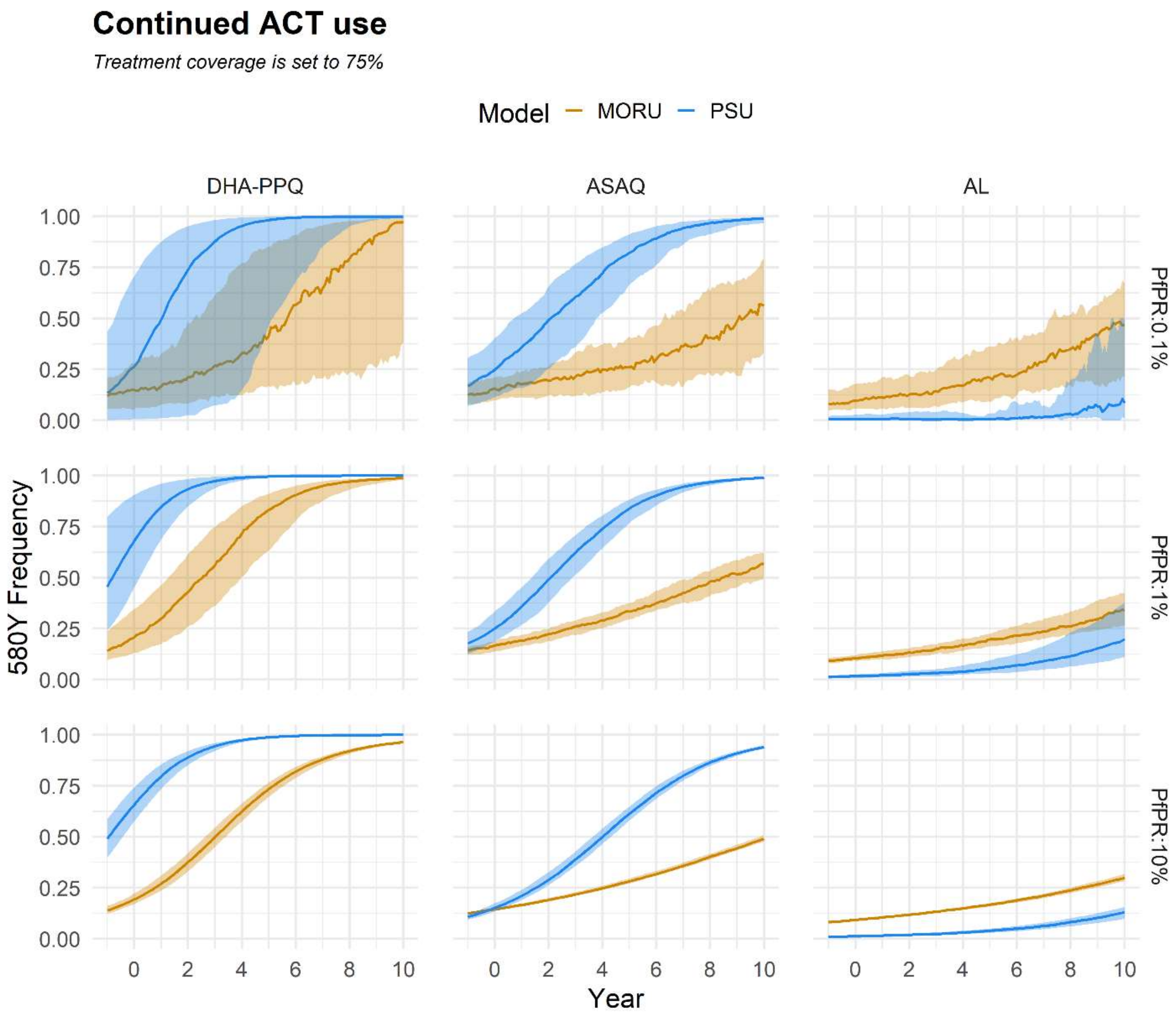

**Supplementary Fig. 32. Expected treatment failure dynamics for continued ACT use.** Each line represents the median treatment failure rate over a 10-year period with continued use of the ACT regimen identified by each column in a setting with Pf prevalence identified in each row. Shaded areas are bounded by the 25th and 75th percentiles obtained from 100 simulations for each scenario. Treatment coverage is set to 25%.

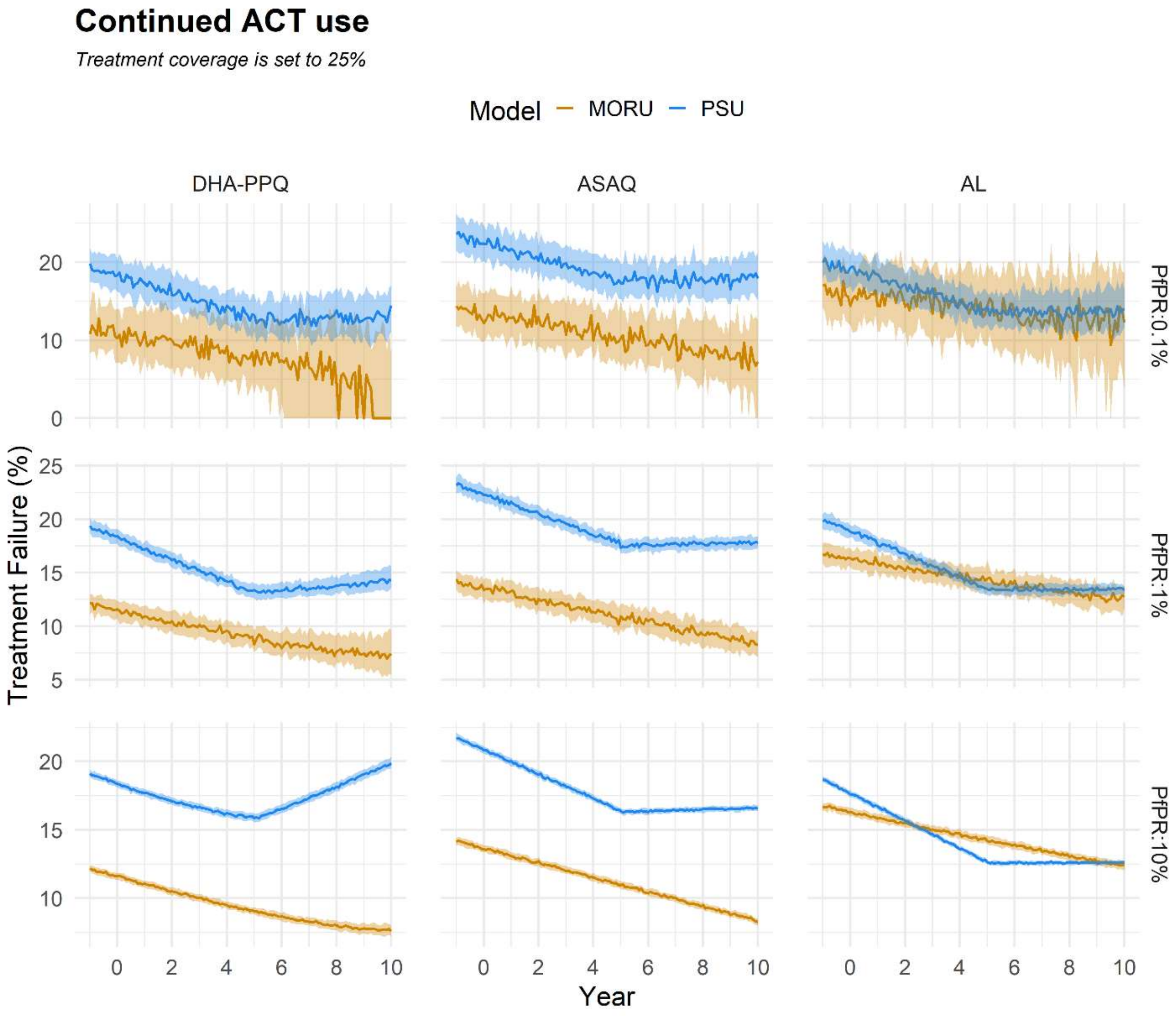

**Supplementary Fig. 33. Expected treatment failure dynamics for continued ACT use.** Each line represents the median treatment failure rate over a 10-year period with continued use of the ACT regimen identified by each column in a setting with Pf prevalence identified in each row. Shaded areas are bounded by the 25th and 75th percentiles obtained from 100 simulations for each scenario. Treatment coverage is set to 75%.

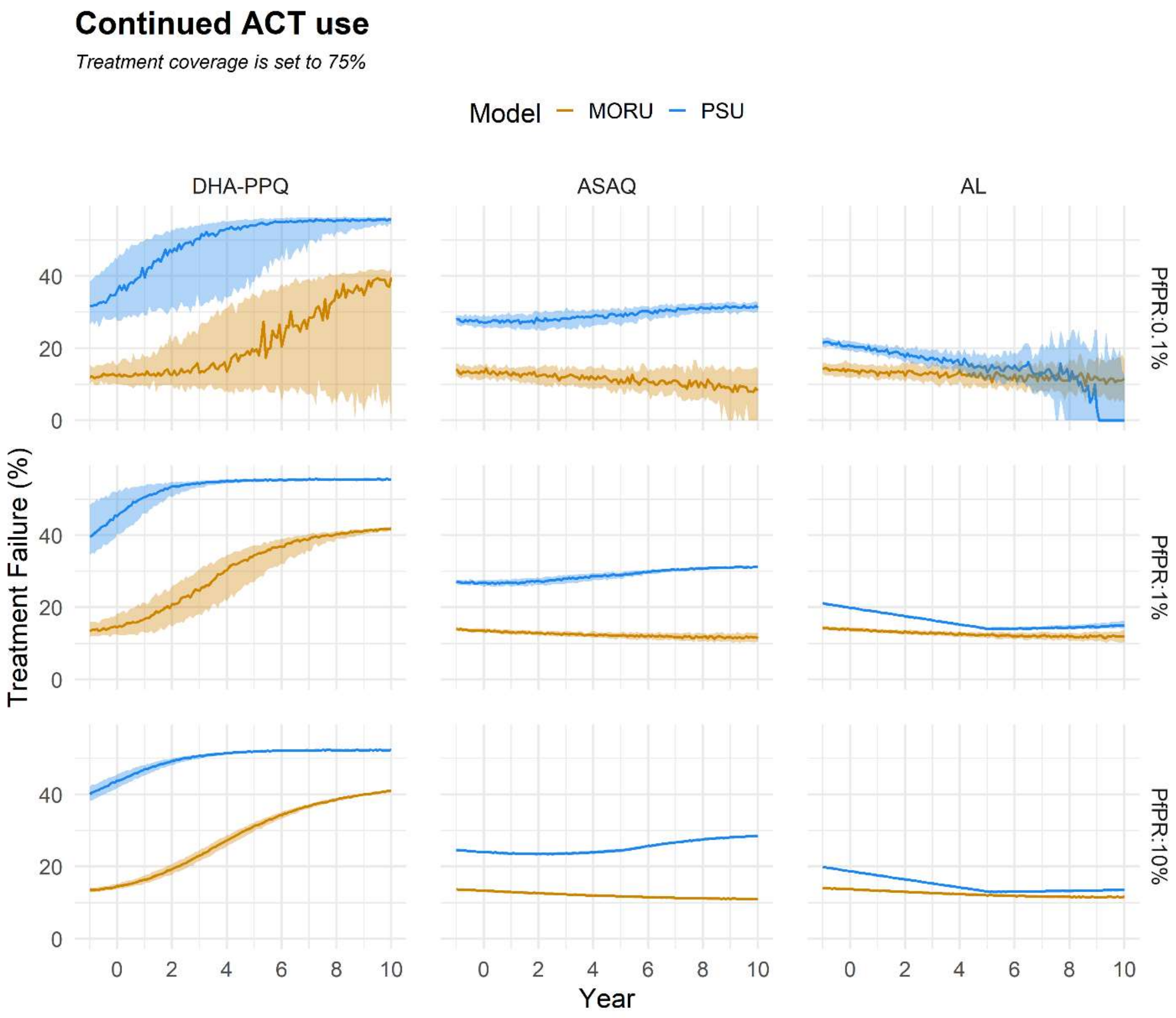

**Supplementary Fig. 34. Expected evolutionary dynamics of each monitored allele with ACT or TACT use.** Each column determines the drug regimen that is put in place for a 10-year period. Both TACTs explored here (ALAQ and ASMQ-PPQ) are compared to DHA-PPQ. Moderate selection pressure is defined by a combination of 0.1% Pf prevalence and 50% treatment coverage, whereas high selection pressure refers to settings with 10% Pf prevalence alongside 75% treatment coverage. Each dotted line illustrates changes in median allelic frequency over time from a set of 100 simulations.

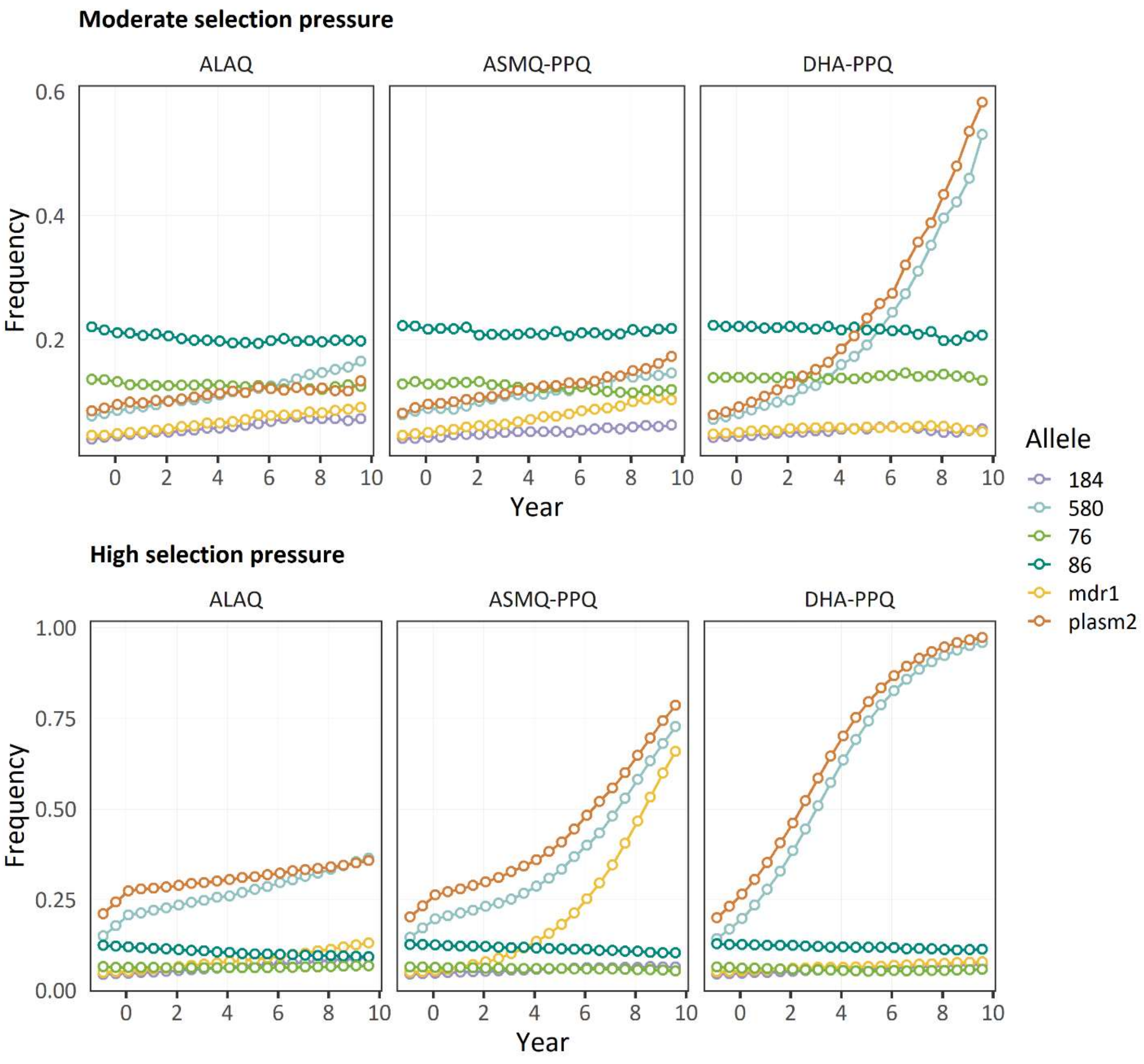

**Supplementary Fig. 35. Expected evolutionary dynamics of each monitored allele with ACT or TACT use.** Each column determines the drug regimen that is put in place for a 10-year period. Both TACTs explored here (ALAQ and ASMQ-PPQ) are compared to AL. Moderate selection pressure is defined by a combination of 0.1% Pf prevalence and 50% treatment coverage, whereas high selection pressure refers to settings with 10% Pf prevalence alongside 75% treatment coverage. Each dotted line illustrates changes in median allelic frequency over time from a set of 100 simulations.

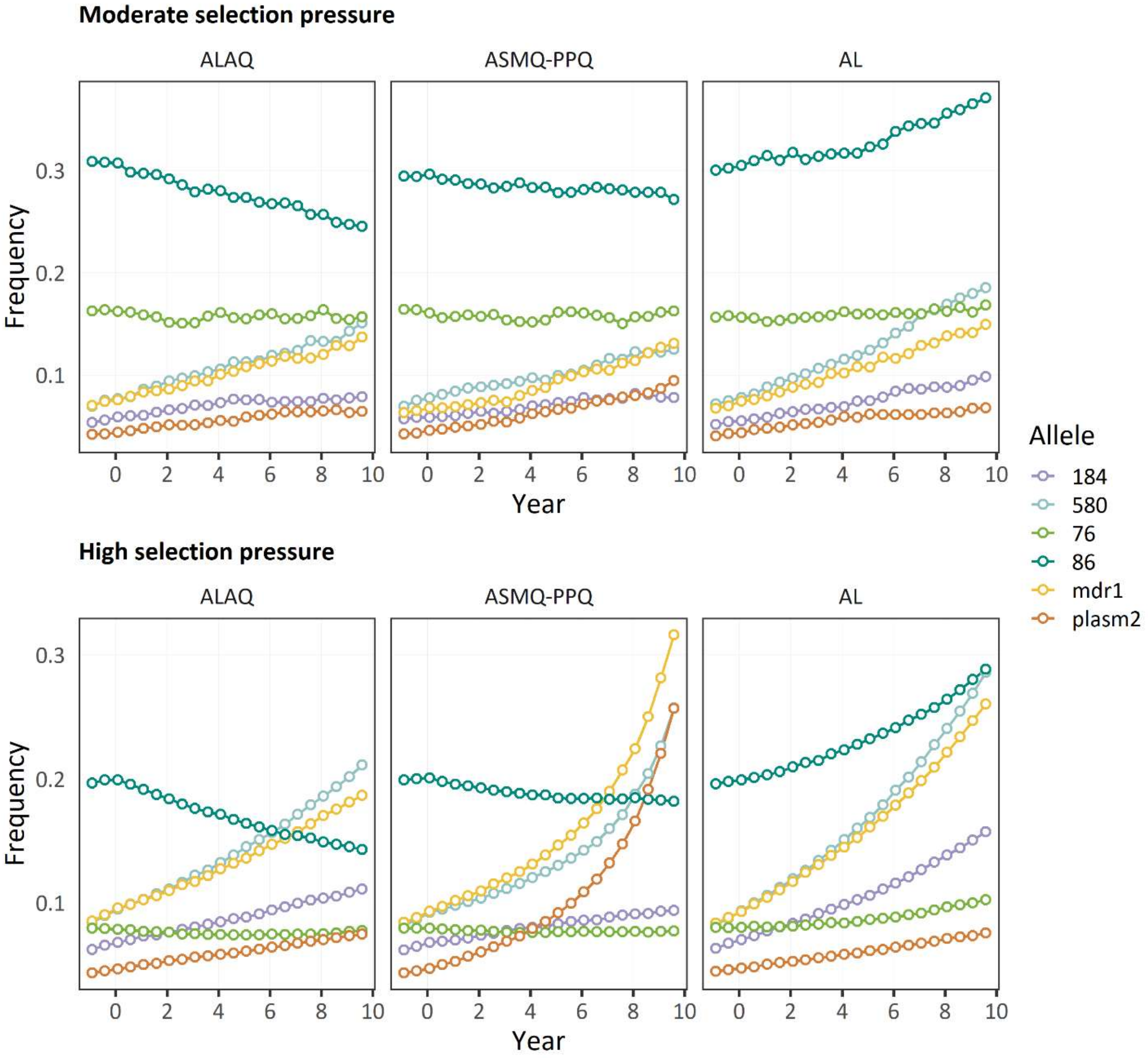

**Supplementary Fig. 36. Expected evolutionary dynamics of each monitored allele with ACT or TACT use.** Each column determines the drug regimen that is put in place for a 10-year period. Both TACTs explored here (ALAQ and ASMQ-PPQ) are compared to ASAQ. Moderate selection pressure is defined by a combination of 0.1% Pf prevalence and 50% treatment coverage, whereas high selection pressure refers to settings with 10% Pf prevalence alongside 75% treatment coverage. Each dotted line illustrates changes in median allelic frequency over time from a set of 100 simulations.

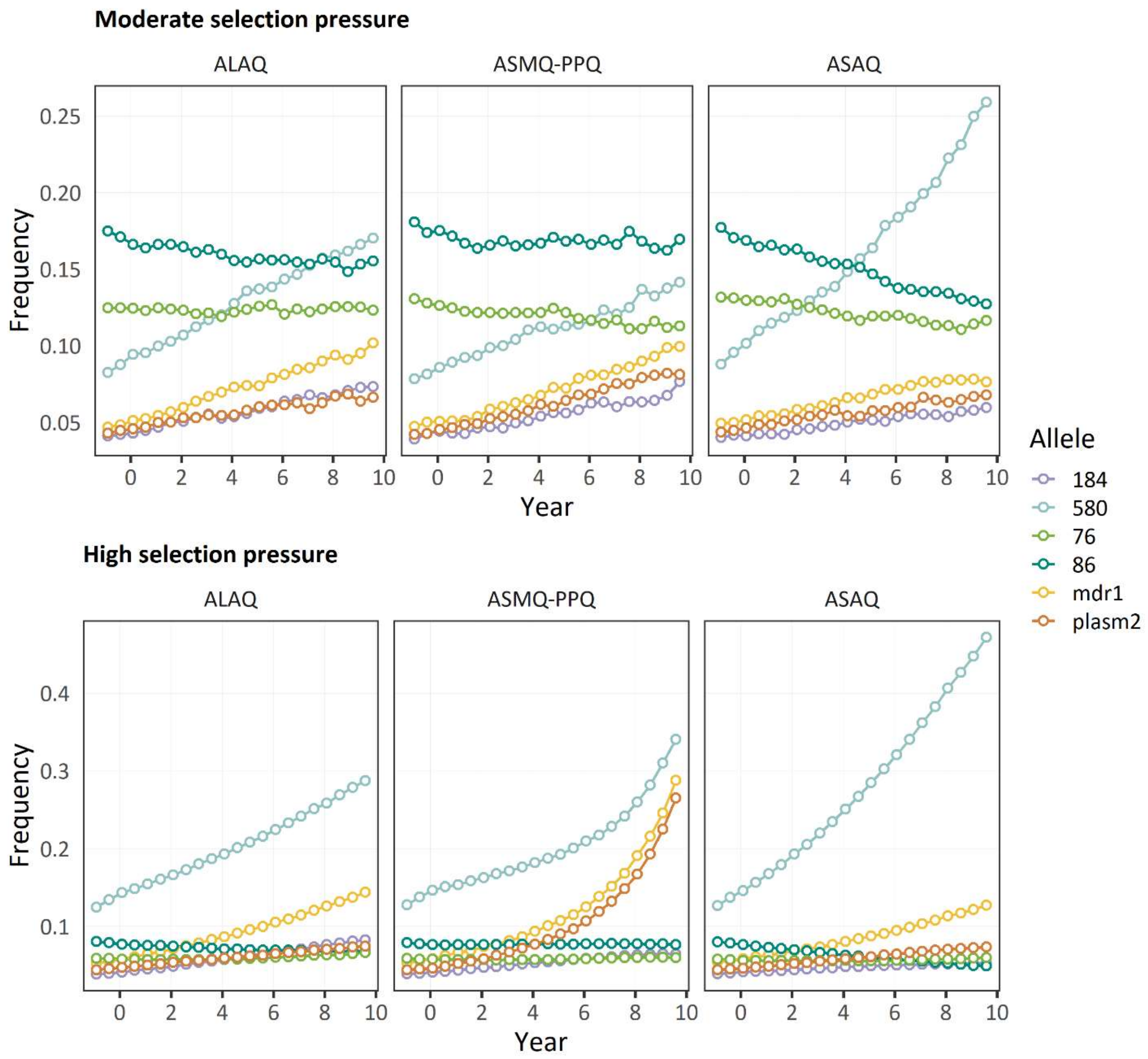

**Supplementary Fig. 37. Relationship between treatment failure rate and 580Y frequency**. Lines connect the median X-Y values for both treatment failure rate and 580Y over a 10-year period from a set of 100 simulations for settings with a given combination of ACT (column) and Pf prevalence (rows). Treatment coverage is set at 50%.

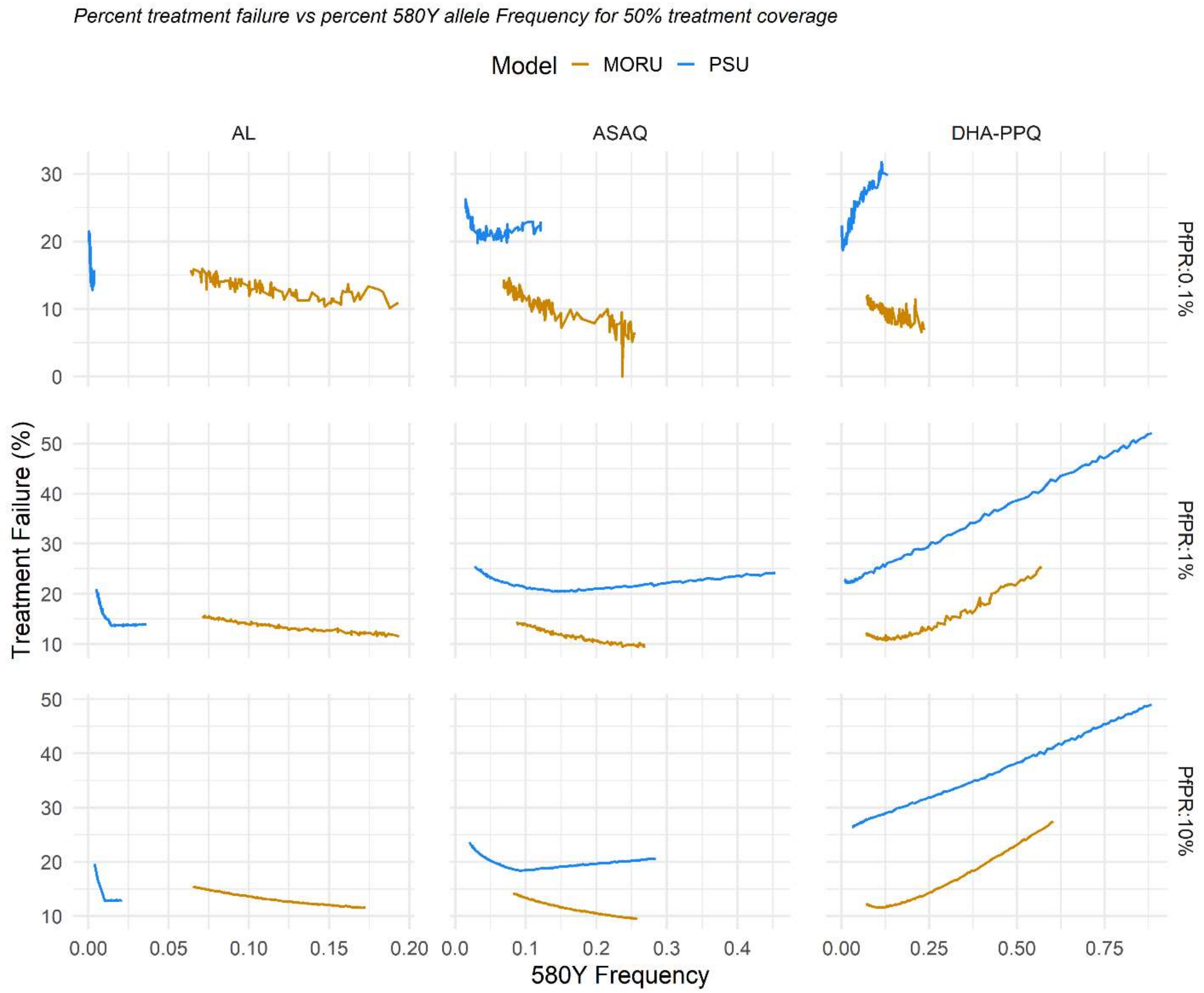

**Supplementary Fig. 38. Relationship between treatment failure rate and 580Y frequency**. Lines connect the median X-Y values for both treatment failure rate and 580Y over a 10-year period from a set of 100 simulations for settings with a given combination of ACT (column) and Pf prevalence (rows). Treatment coverage is set at 25%.

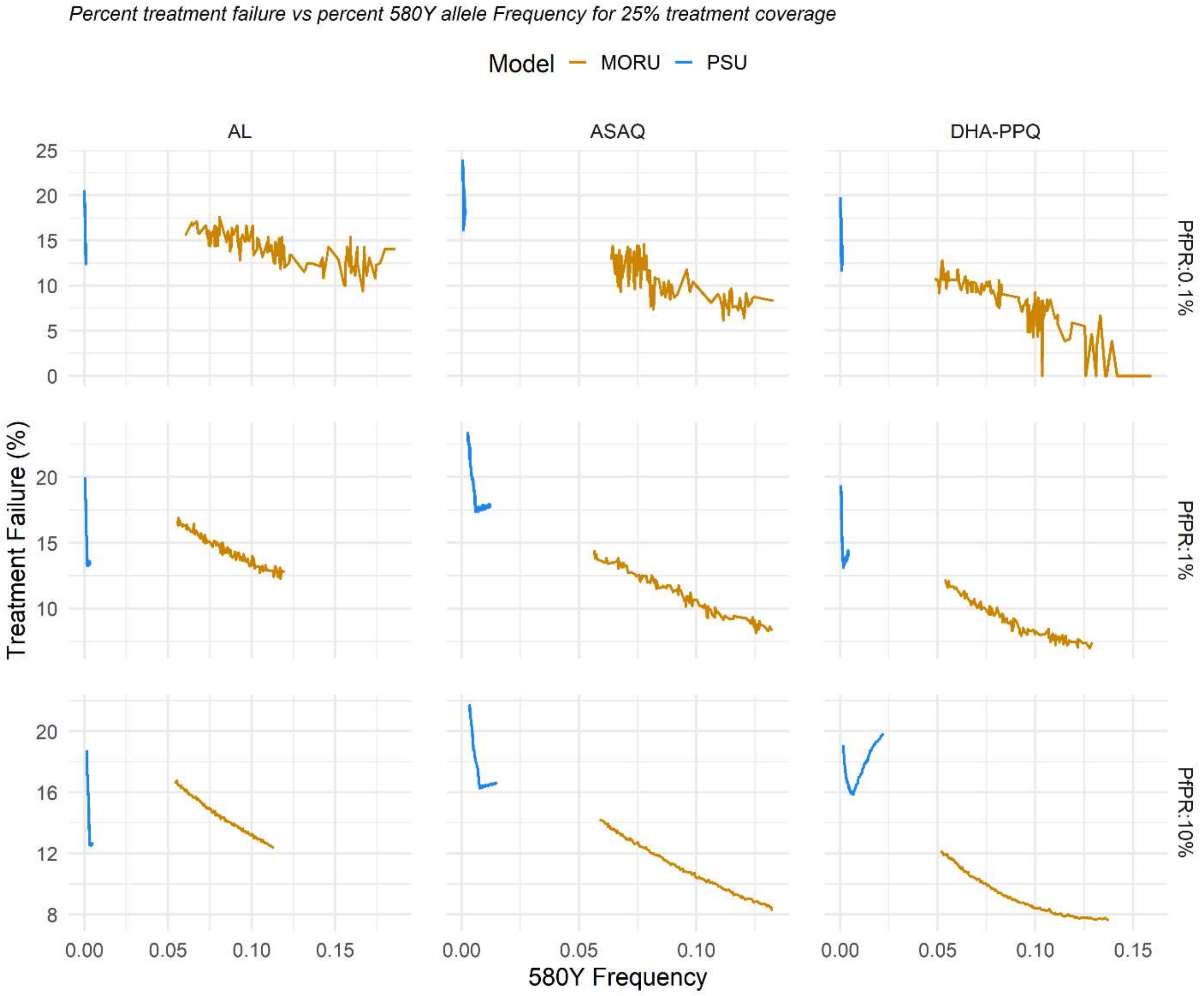

**Supplementary Fig. 39. Relationship between treatment failure rate and 580Y frequency**. Lines connect the median X-Y values for both treatment failure rate and 580Y over a 10-year period from a set of 100 simulations for settings with a given combination of ACT (column) and Pf prevalence (rows). Treatment coverage is set at 75%.

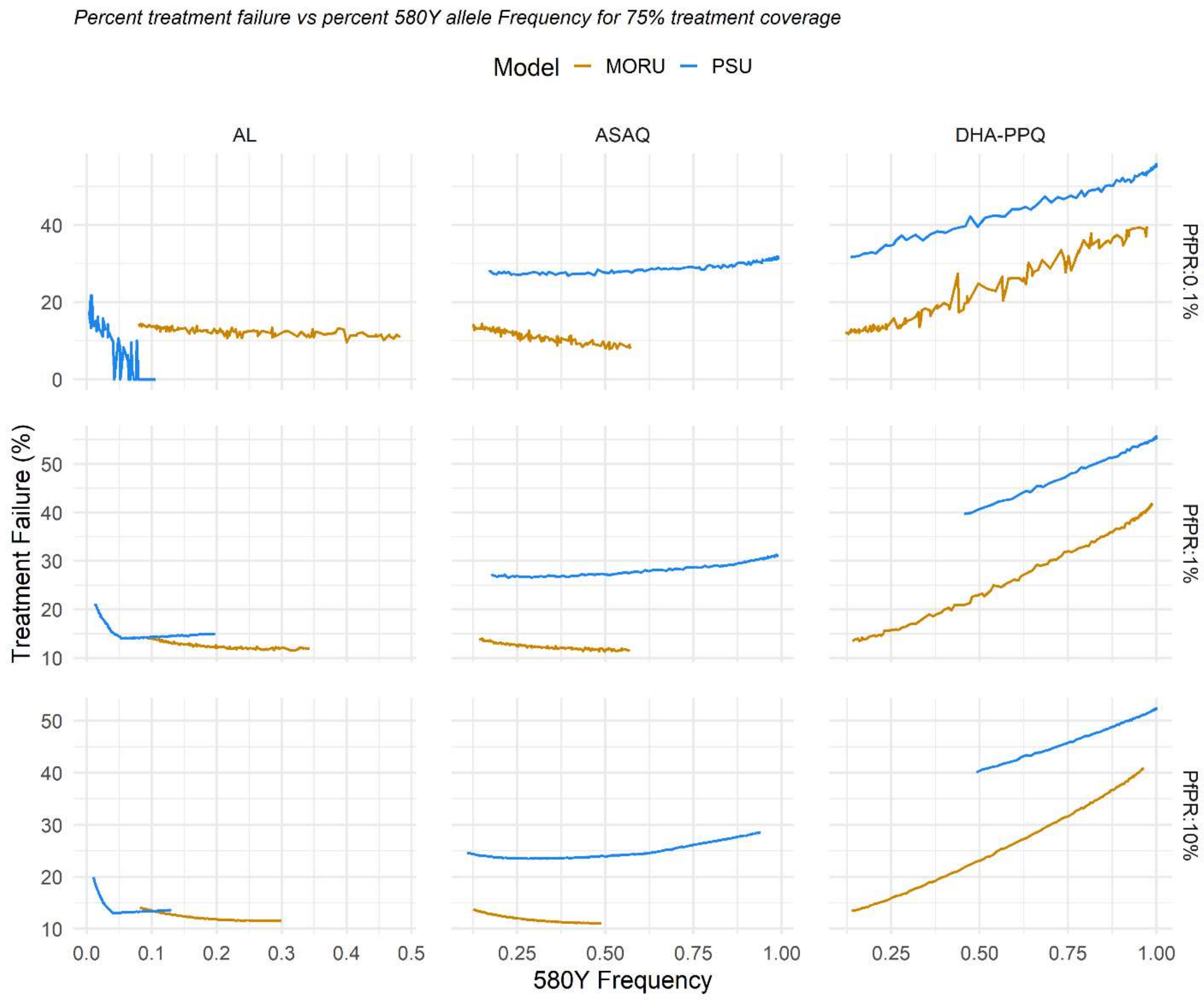

**Supplementary Fig. 40. Effects of delaying TACT introduction predicted by the MORU model**. In a population of 1,000,000 individuals, panels show the evolution of 580Y allele frequency over a period of 10 years under different transmission intensities: 0.1% PfPR (top row), 1% PfPR (middle row), 10% PfPR (bottom row). In these scenarios, treatment coverage is 50%, and DHA-PPQ was used as first-line ACT before switching to TACTs. The colors show the differences in delayed adoption of the TACT ranging from zero years delay (light yellow) to a five-year delay (black) delay. Left panels show results when switching from DHA-PPQ to ALAQ, while right panels show results when switching from DHA-PPQ to ASMQ-PPQ scenarios.  The dots on the graph show the switch points for each trajectory; six lines are shown for delays of 0, 1, 2, 3, 4, and 5 years.

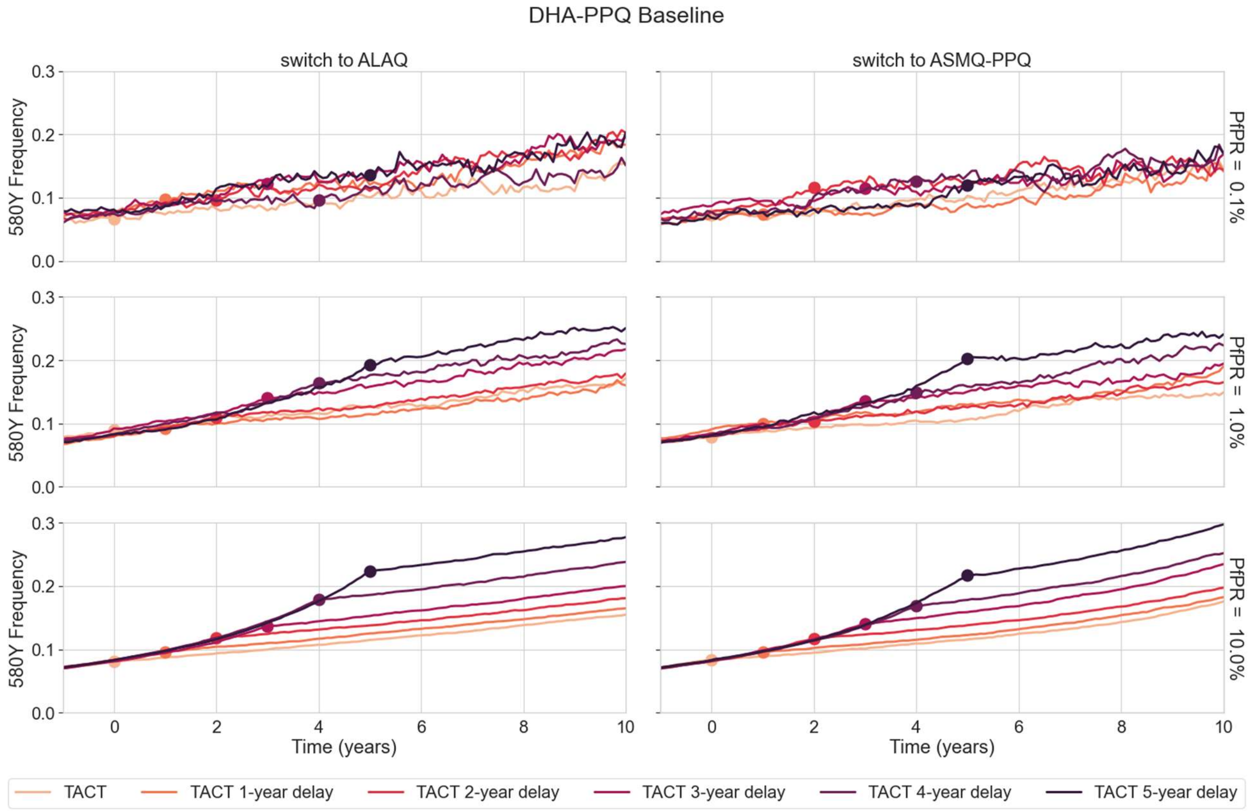

**Supplementary Fig. 41.** Same as Supplementary Fig. 40, with the results from PSU model.

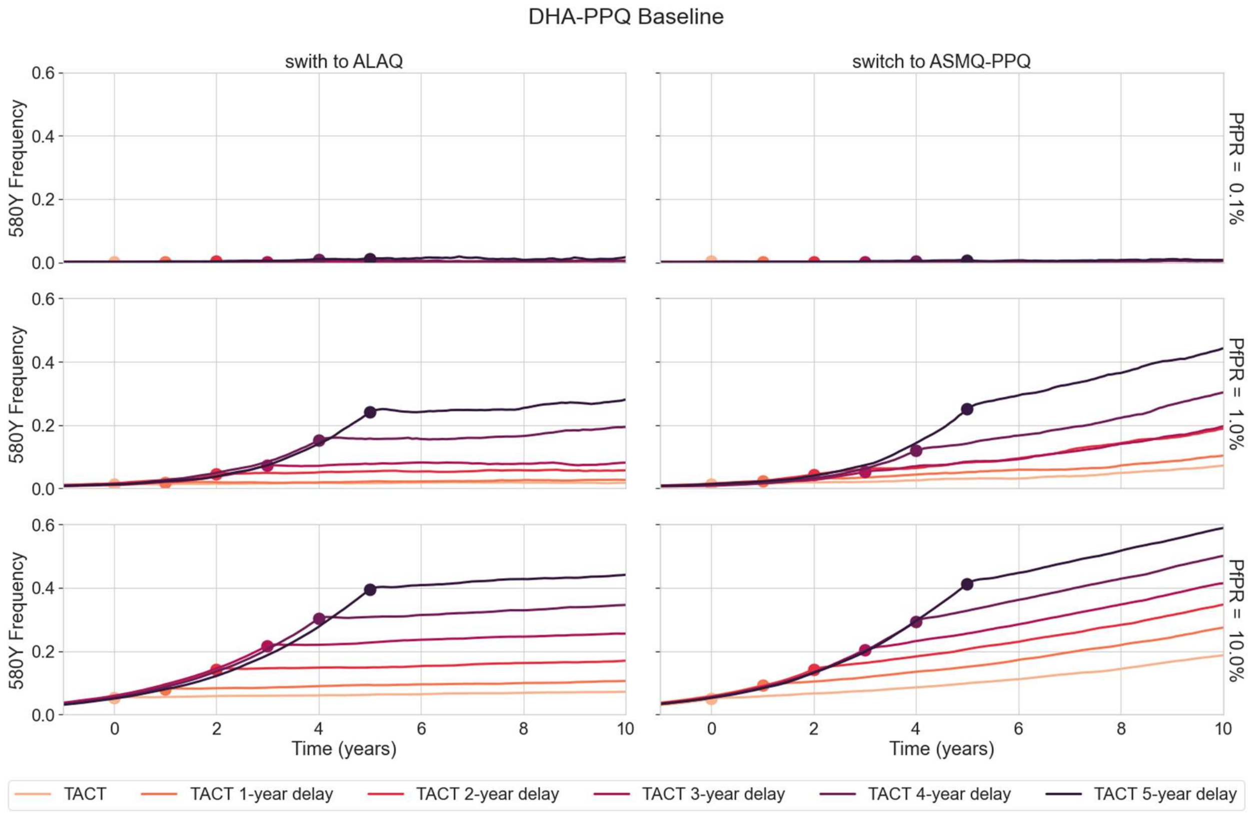

**Supplementary Fig. 42.** Same as Supplementary Fig. 40, with the results from MORU model. In these scenarios, ASAQ was used as first-line ACT before switching to TACTs.

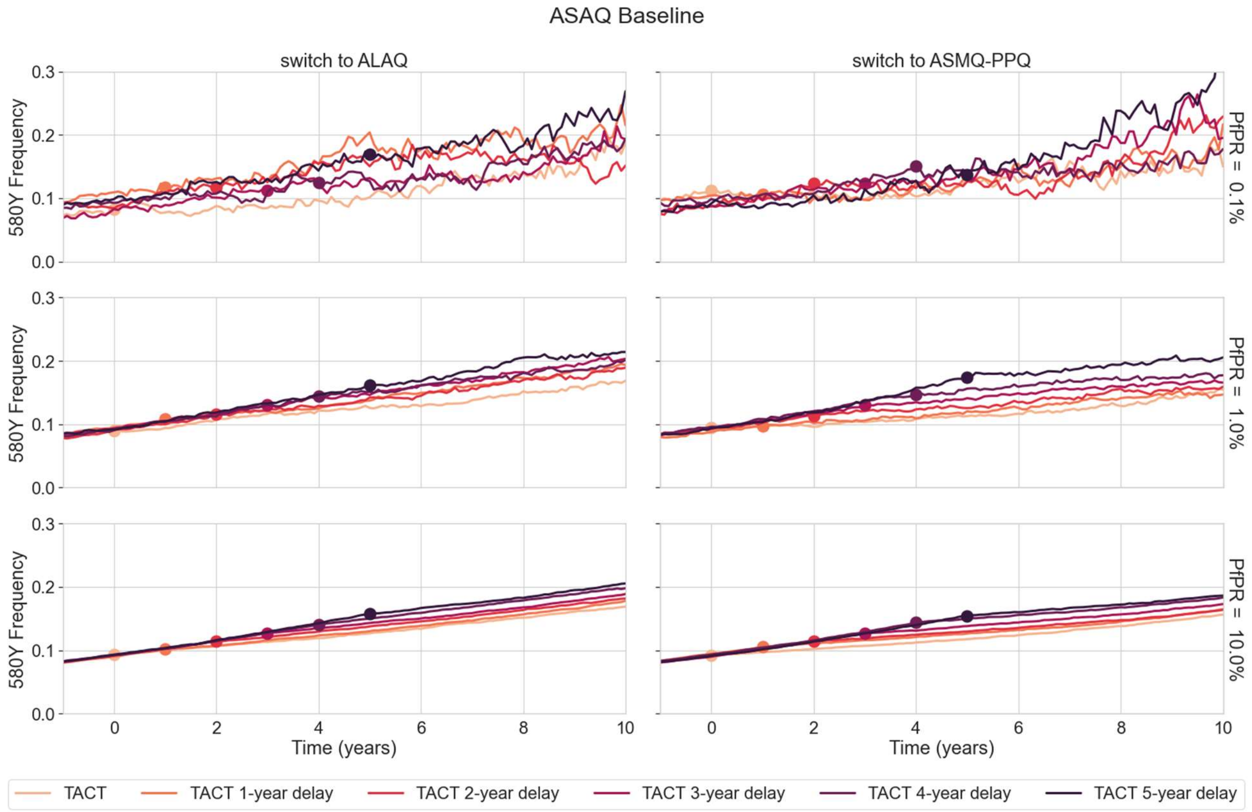

**Supplementary Fig. 43.** Same as Supplementary Fig. 42, with the results from PSU model.

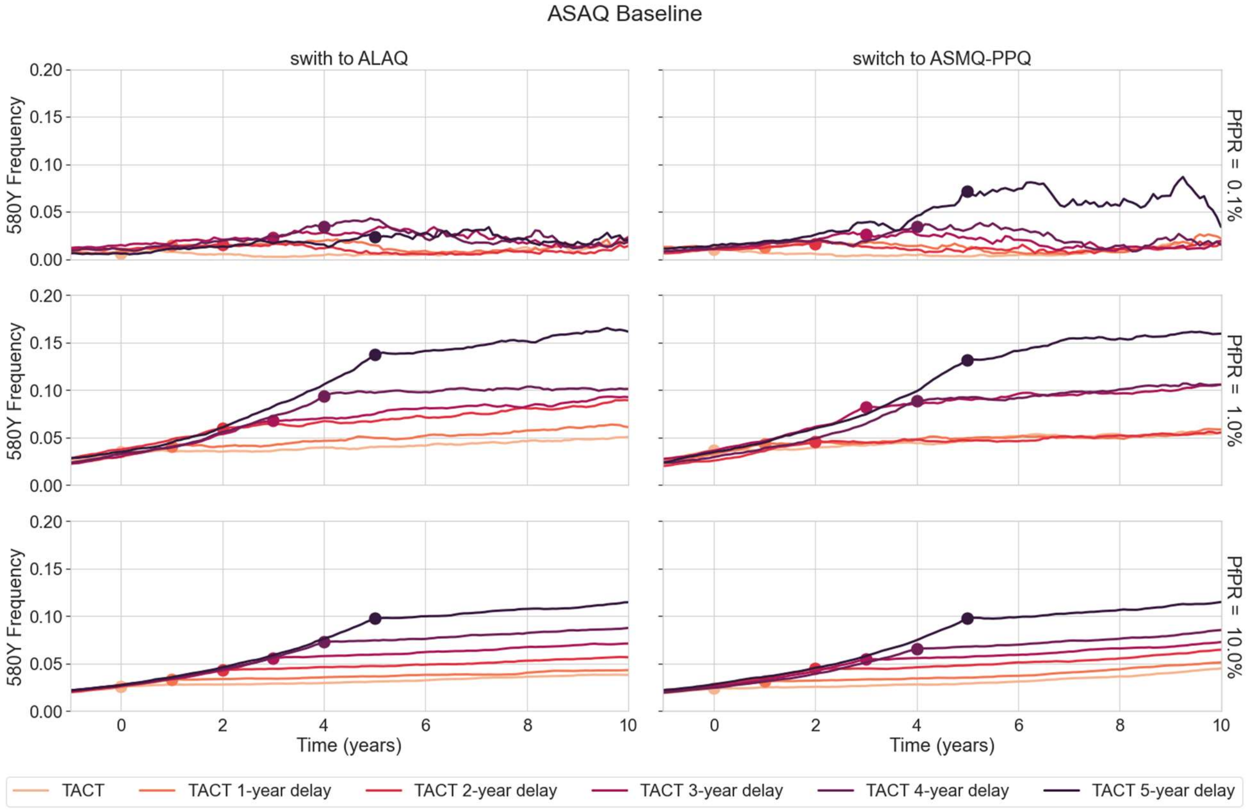

**Supplementary Fig. 44.** Same as Supplementary Fig. 40, with the results from MORU model. In these scenarios, AL was used as first-line ACT before switching to TACTs.

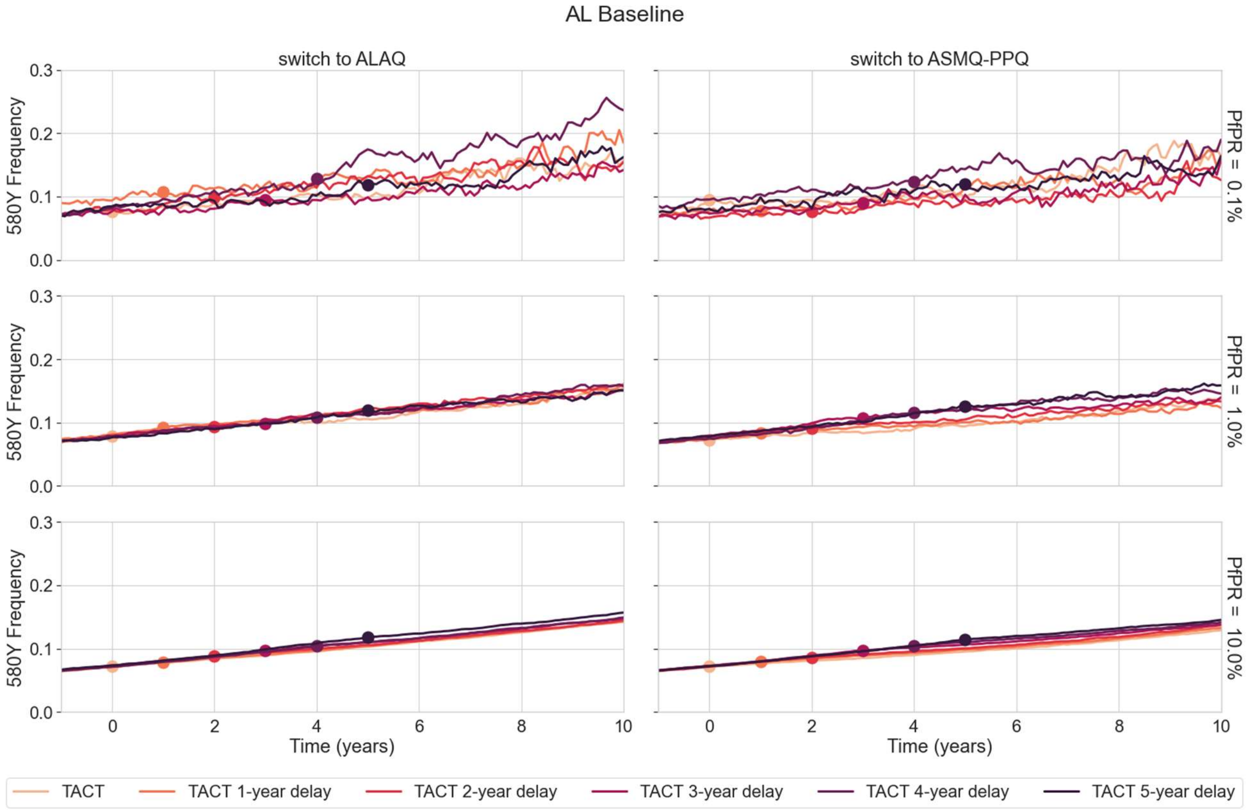

**Supplementary Fig. 45.** Same as Supplementary Fig. 44, with the results from PSU model.

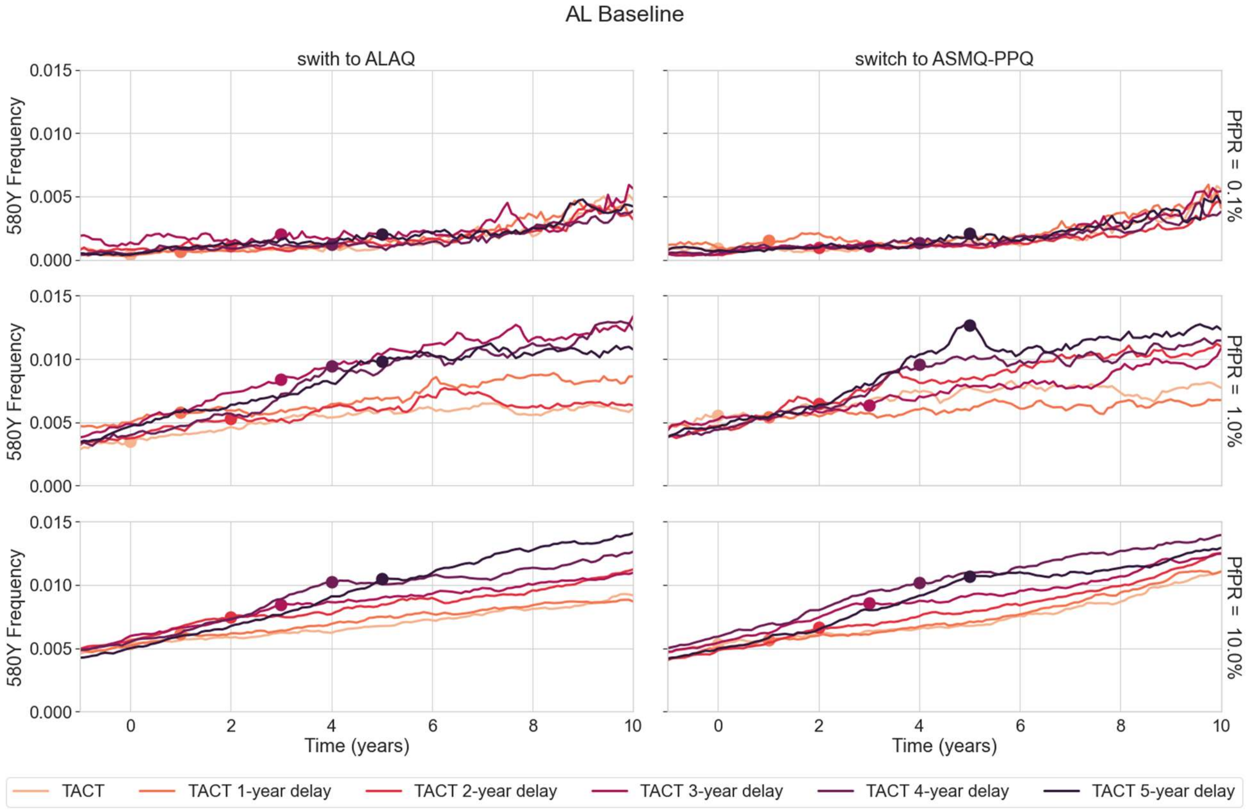

**Supplementary Fig. 46.** Same with Supplementary Fig. 40, with results from MORU model. The panels show the treatment failures rate over a period of 10 years. In those scenarios, DHA-PPQ was used as first-line ACT before switching to TACTs.

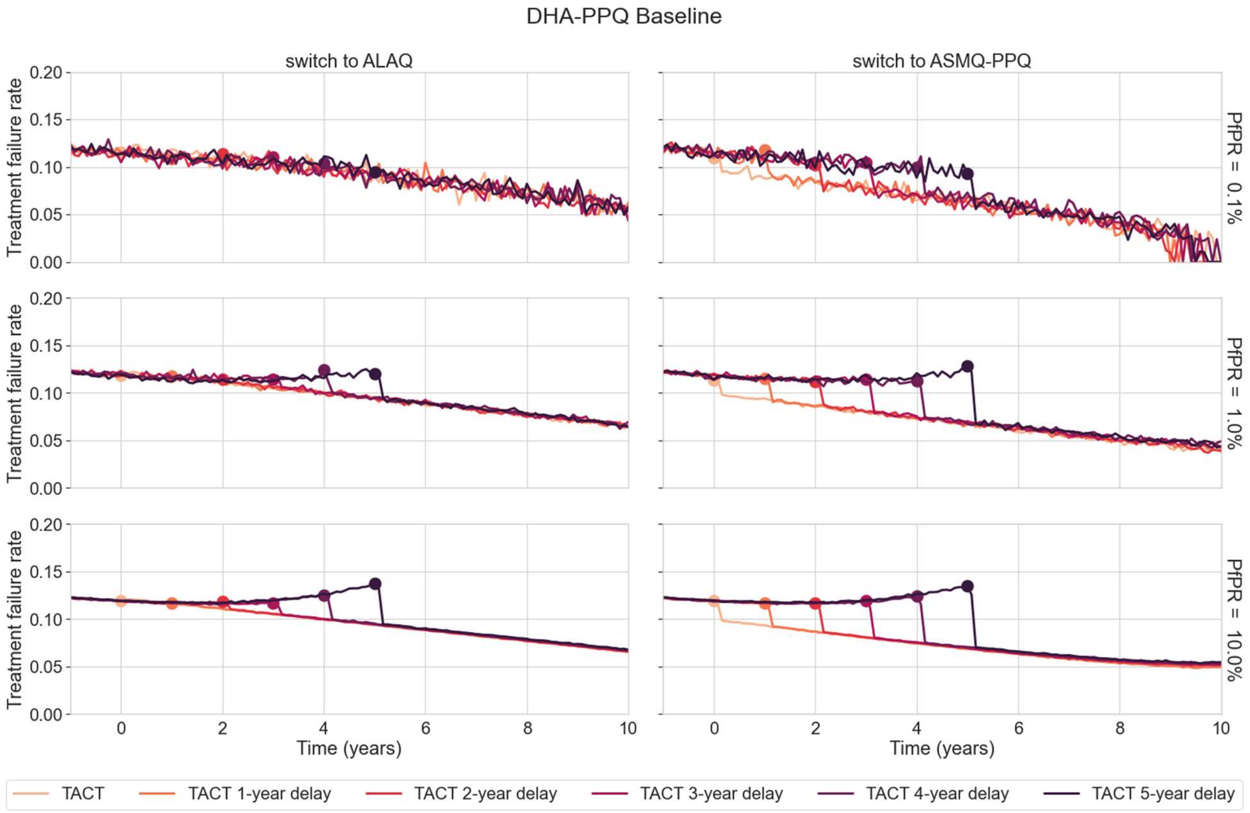

**Supplementary Fig. 47.** Same as Supplementary Fig. 46, with the results from PSU model.

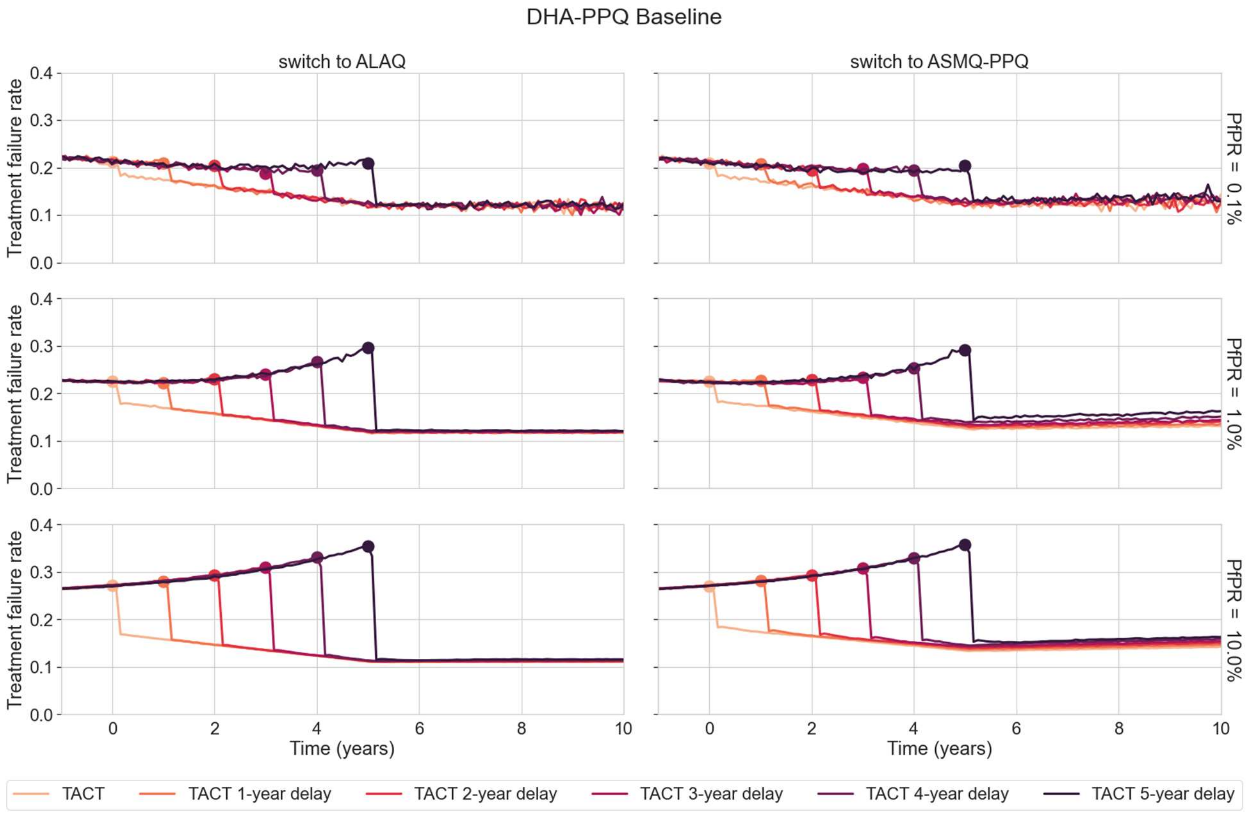

**Supplementary Fig. 48.** Same as Supplementary Fig. 46, with the results from MORU model. In these scenarios, ASAQ was used as first-line ACT before switching to TACTs.

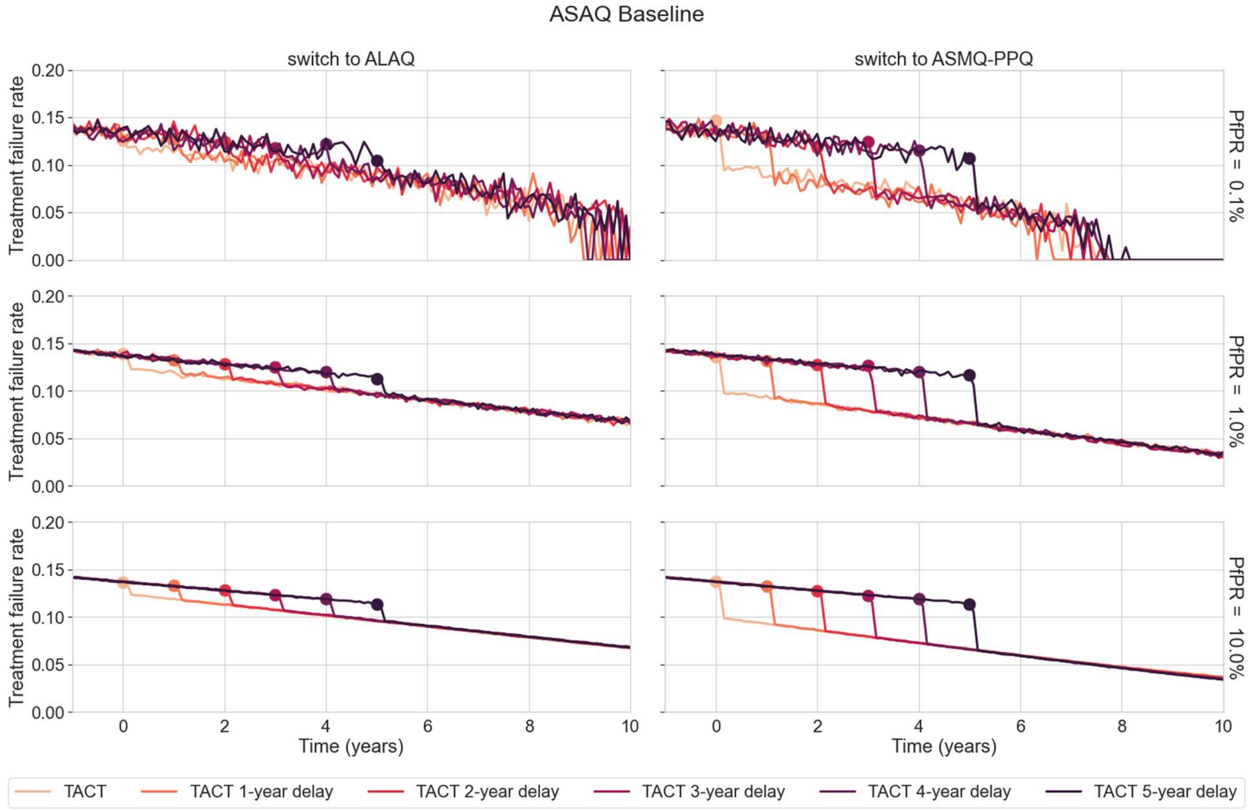

**Supplementary Fig. 49.** Same as Supplementary Fig. 48, with the results from PSU model.

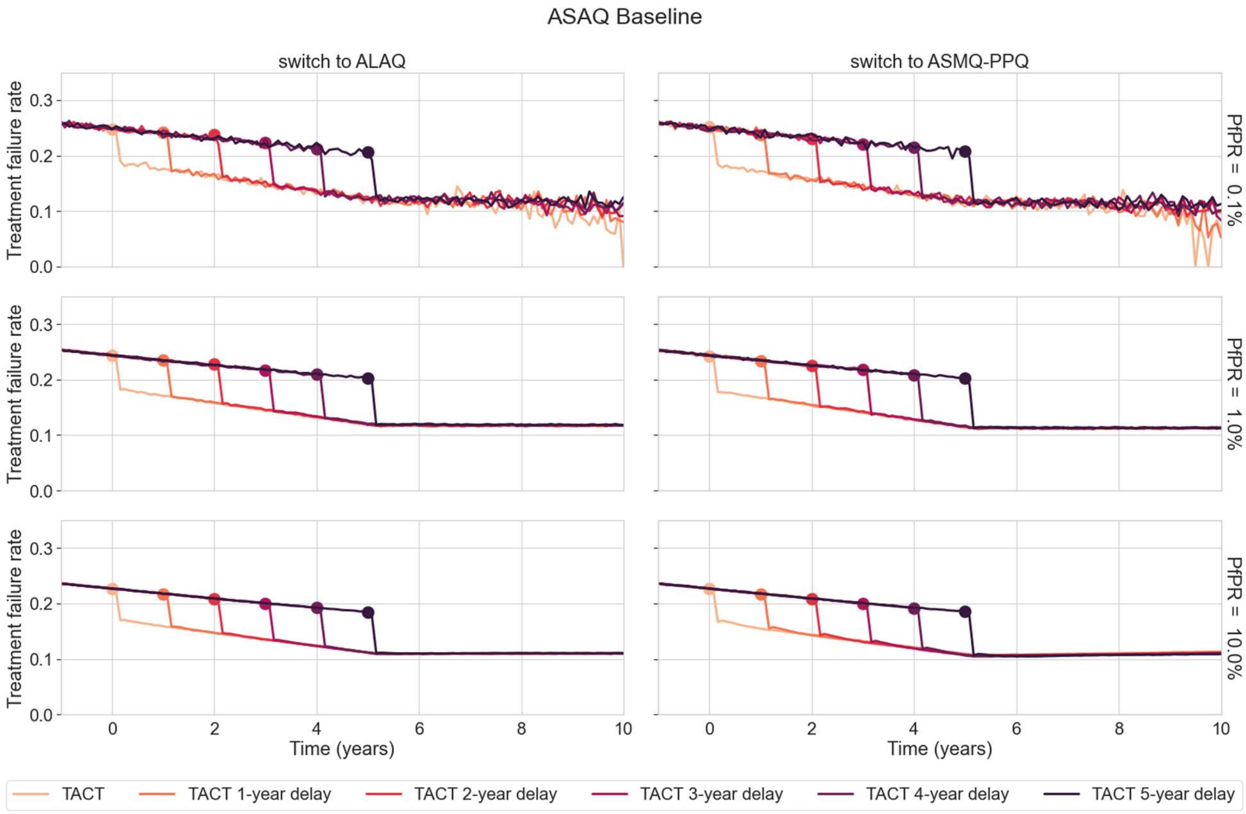

**Supplementary Fig. 50.** Same as Supplementary Fig. 46, with the results from MORU model. In these scenarios, AL was used as first-line ACT before switching to TACTs.

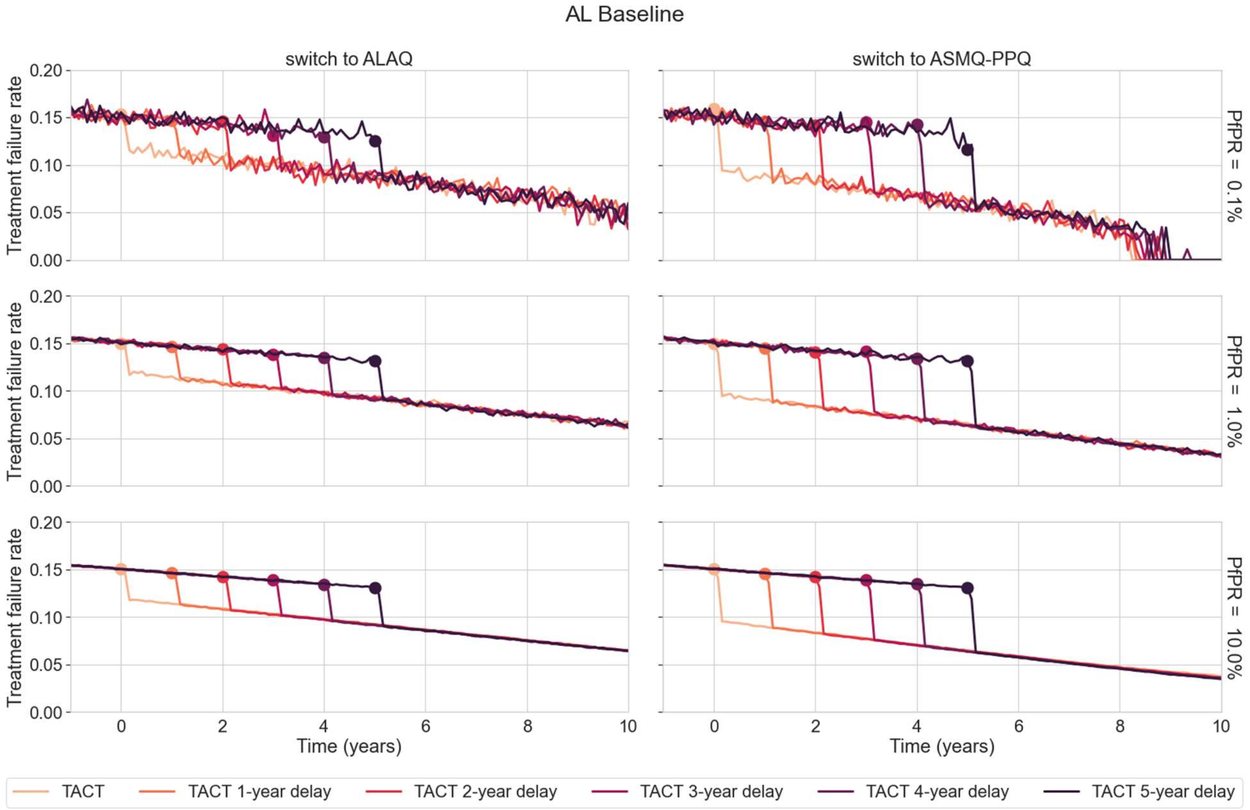

**Supplementary Fig. 51.** Same as Supplementary Fig. 50, with the results from PSU model.

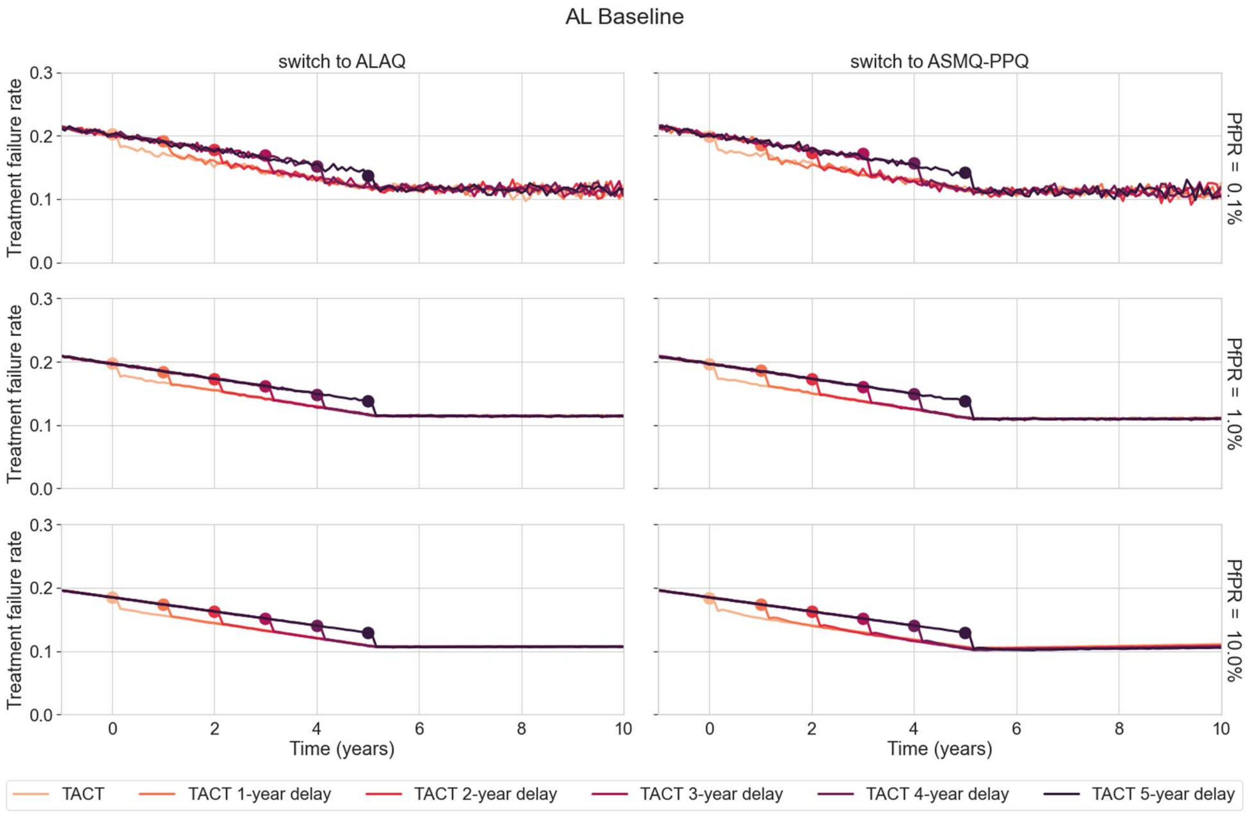

**Supplementary Fig. 52. Comparison of ACT and TACT deployment at 1% PfPR and 50% treatment coverage.** AL is used as the baseline ACT before TACTs are deployed at year zero; grey lines (medians from 100 simulations) show the evolution of the pfkelch13 580Y allele or treatment failure rates under continued DHA-PPQ use. Red lines show how these processes are slowed down by deployment of ASMQ-PPQ. Blue lines show how these processes are slowed down by deployment of ALAQ. All shaded areas show inter-quartile ranges. Panels above each graph show an individual's relative risk of 580Y infection (under TACT deployment versus ACT deployment) after 2, 5, and 10 years of deployment; bars show 95% confidence intervals and the dot indicates the median, assuming n=1000 for each deployment.

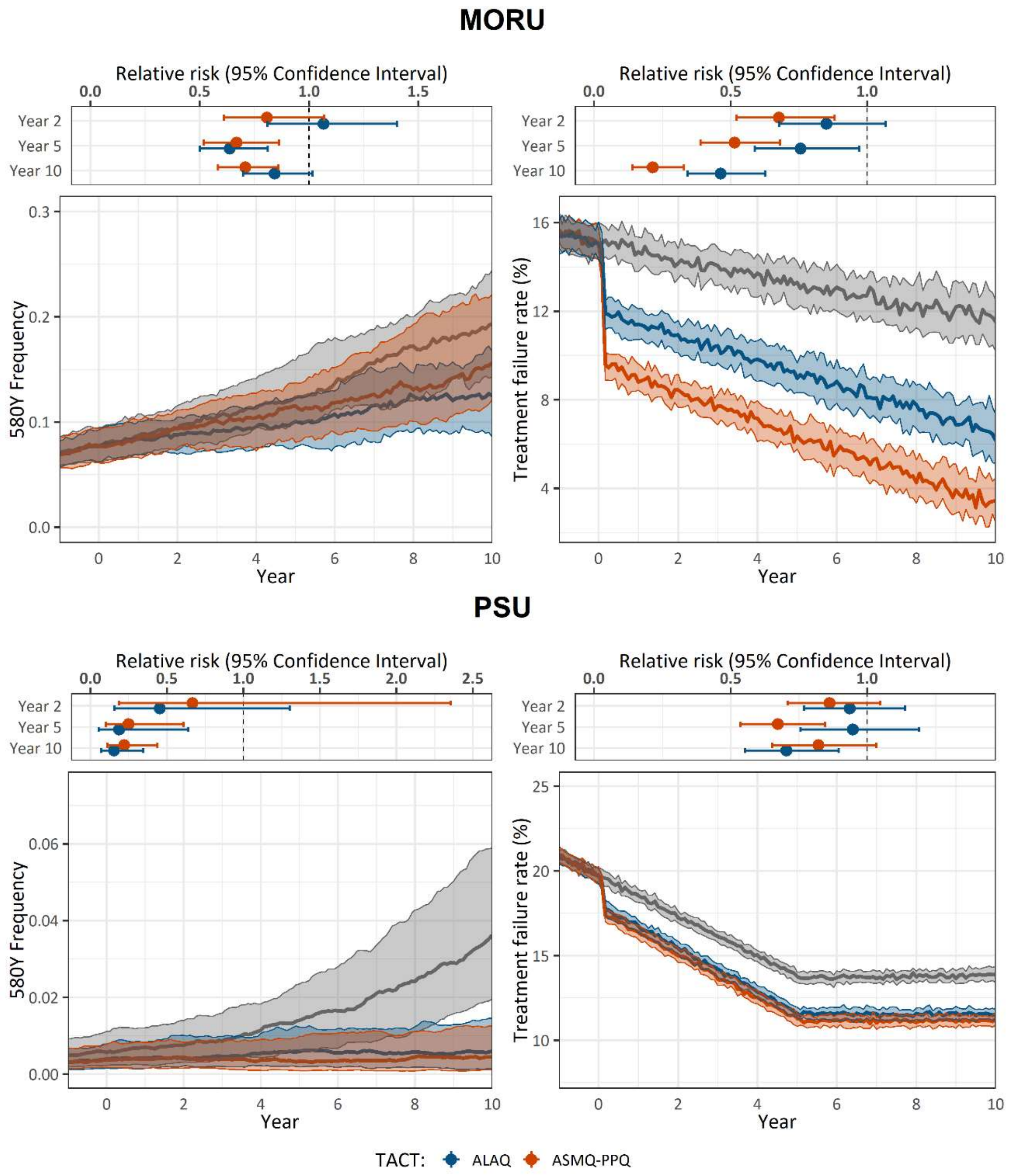

**Supplementary Fig. 53. Comparison of ACT and TACT deployment at 1% PfPR and 50% treatment coverage.** ASAQ is used as the baseline ACT before TACTs are deployed at year zero; grey lines (medians from 100 simulations) show the evolution of the pfkelch13 580Y allele or treatment failure rates under continued DHA-PPQ use. Red lines show how these processes are slowed down by deployment of ASMQ-PPQ.  Blue lines show how these processes are slowed down by deployment of ALAQ. All shaded areas show inter-quartile ranges. Panels above each graph show an individual's relative risk of 580Y infection (under TACT deployment versus ACT deployment) after 2, 5, and 10 years of deployment; bars show 95% confidence intervals and dots indicates the median, assuming n=1000 for each deployment.

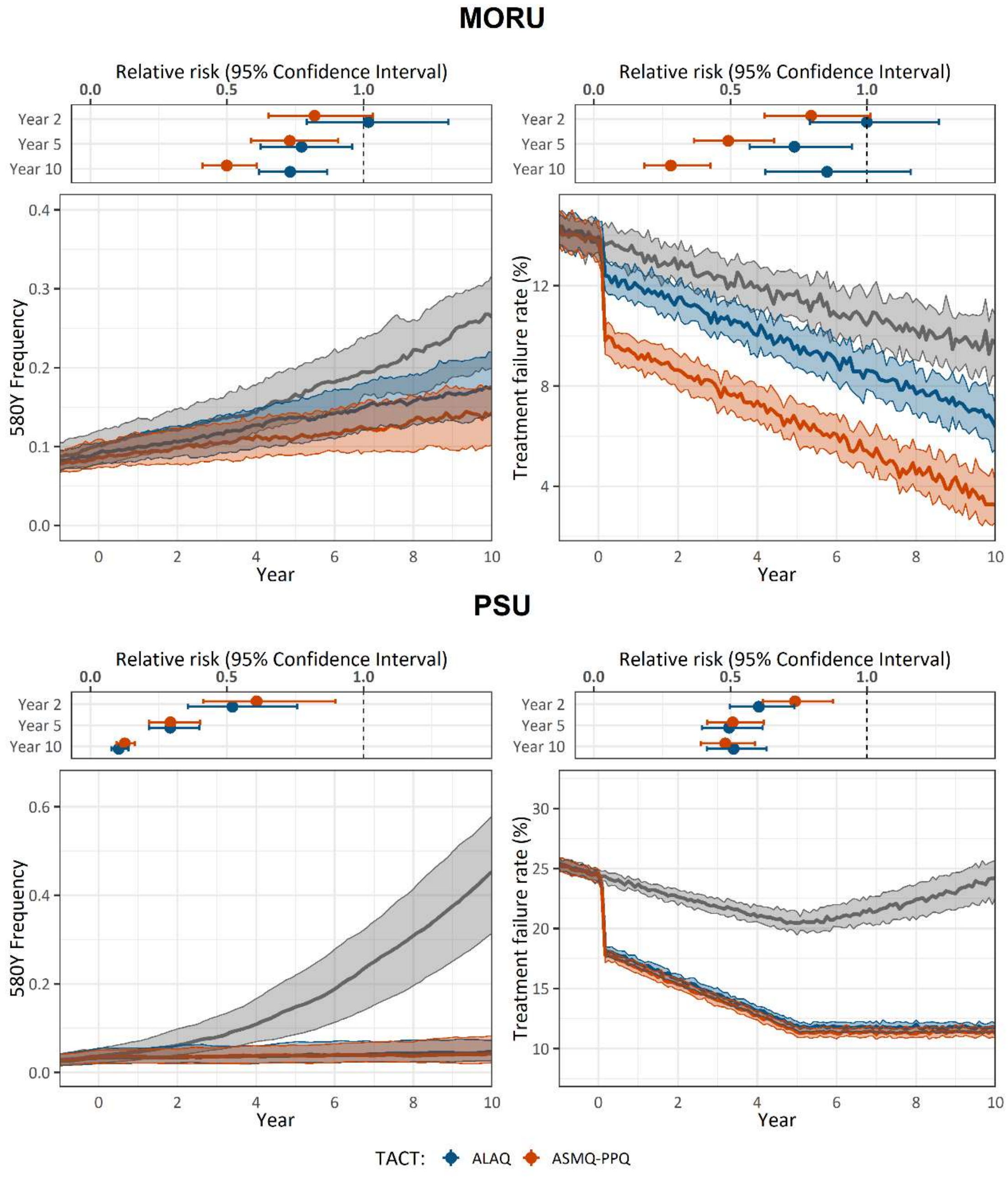

**Supplementary Fig. 54. Effect of gradual TACT adoption on 580Y frequency and treatment failure.** In a population of 1,000,000 individuals, panels show the comparisons of 580Y allele frequency (top row) and treatment failure rate (bottom row) at year 10 under different transmission intensities: 0.1% PfPR (left column), 1% PfPR (middle column), 10% PfPR (right column). In these scenarios, DHAPPQ is used as the baseline therapy before TACTs starts to be adopted at year zero. The adoption of TACTs occurs in the public sector only with the initial proportion is 10% TACT and 90% regular ACT. Then, the adoption takes place over 3, 5, 7, or 9 years. Colors shows the TACTs used. Each boxplot summarizes 100 simulations, where the boxes indicate the interquartile range (IQR), the whiskers extend to 1.5 times the IQR; remaining points (outliers) are plotted individually as diamonds.

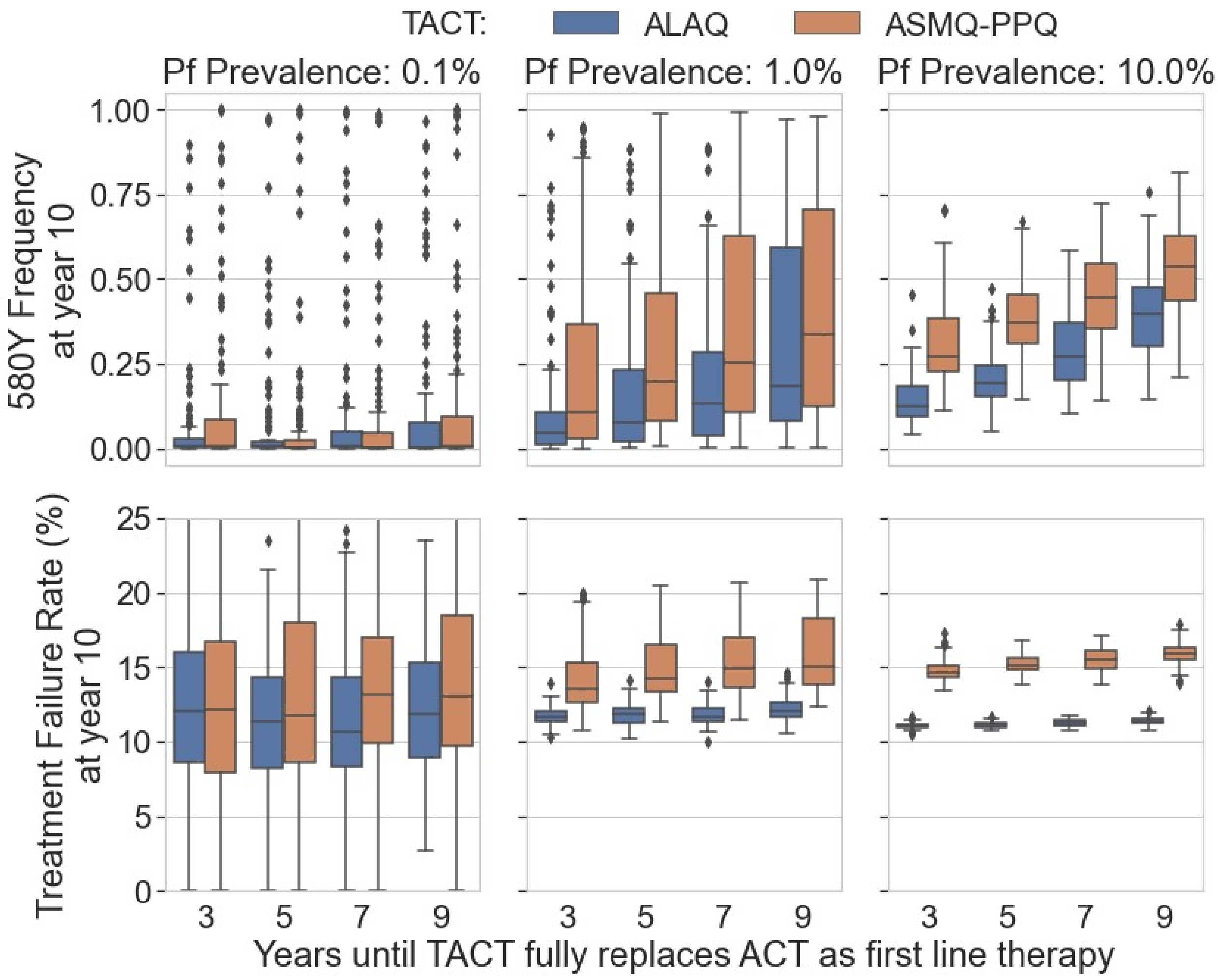

**Supplementary Fig. 55. Modelling workflow.** Illustrates elements of how models were parametrized, and their initial conditions set, how mutation rates were aligned to produce a similar number of mutants across models, and how drug usage for uncomplicated malaria treatments was assumed to change over time. We assume a linear increase in first line therapy (FLT) access through the public sector until it plateaus at year 5. All other treatment options are accessed from private providers. These illustrative runs were performed with 50% treatment coverage, aiming for 1% Pf. prevalence (as detected by microscopy).

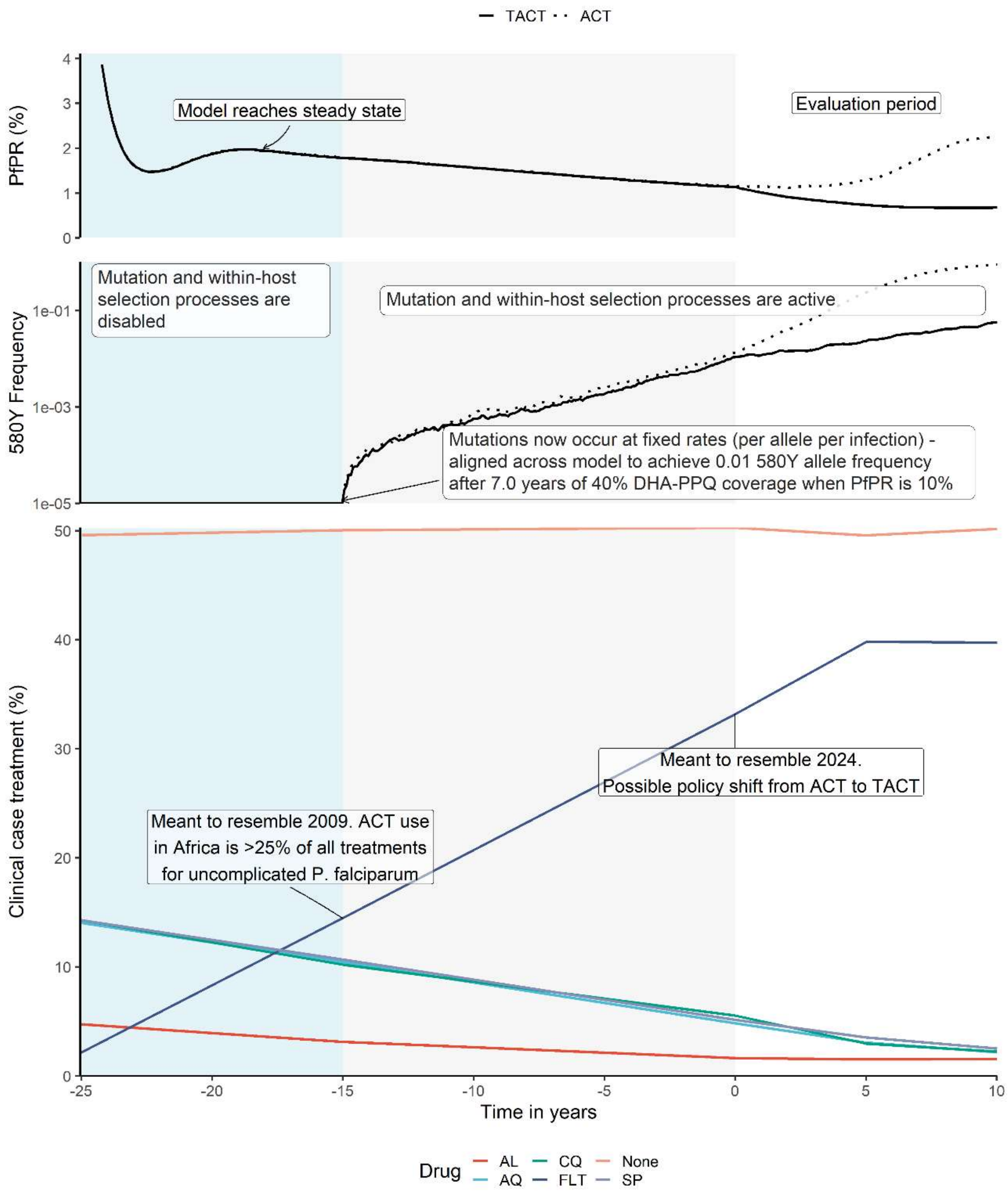

Supplement: Supplementary file 1 — Supplementary Information [file 41467_2023_39914_MOESM1_ESM.pdf]
