# Supplementary material for: Preventing antimalarial drug resistance with triple artemisinin-based combination therapies
Source: Nat Commun. 2023 Jul 29;14:4568. doi: 10.1038/s41467-023-39914-3 (PMC10387089; doi:10.1038/s41467-023-39914-3)
Supplement: Supplementary file 3 — Reporting Summary [file 41467_2023_39914_MOESM3_ESM.pdf]

nature portfolio

Corresponding author(s): Ricardo Aguas (ricardo.aguas@ndm.ox.ac.uk)

Last updated by author(s): Jun 16, 2023

Reporting Summary

Nature Portfolio wishes to improve the reproducibility of the work that we publish. This form provides structure for consistency and transparency in reporting. For further information on Nature Portfolio policies, see our Editorial Policies and the Editorial Policy Checklist.

Statistics

For all statistical analyses, confirm that the following items are present in the figure legend, table legend, main text, or Methods section.

n/a	Confirmed	
☐	☒	The exact sample size (*n*) for each experimental group/condition, given as a discrete number and unit of measurement
☐	☒	A statement on whether measurements were taken from distinct samples or whether the same sample was measured repeatedly
☐	☒	The statistical test(s) used AND whether they are one- or two-sided<br>*Only common tests should be described solely by name; describe more complex techniques in the Methods section.*
☒	☐	A description of all covariates tested
☐	☒	A description of any assumptions or corrections, such as tests of normality and adjustment for multiple comparisons
☐	☒	A full description of the statistical parameters including central tendency (e.g. means) or other basic estimates (e.g. regression coefficient) AND variation (e.g. standard deviation) or associated estimates of uncertainty (e.g. confidence intervals)
☒	☐	For null hypothesis testing, the test statistic (e.g. *F*, *t*, *r*) with confidence intervals, effect sizes, degrees of freedom and *P* value noted<br>*Give P values as exact values whenever suitable.*
☒	☐	For Bayesian analysis, information on the choice of priors and Markov chain Monte Carlo settings
☒	☐	For hierarchical and complex designs, identification of the appropriate level for tests and full reporting of outcomes
☒	☐	Estimates of effect sizes (e.g. Cohen's *d*, Pearson's *r*), indicating how they were calculated

Our web collection on statistics for biologists contains articles on many of the points above.

Software and code

Policy information about availability of computer code

Data collection	A repository with the compiled simulation outputs can be found here: https://github.com/Longterm-deployment-of-TACTs/Longterm-deployment-of-TACTs .
Data analysis	Both models' code has been made available at: https://github.com/Longterm-deployment-of-TACTs/Longterm-deployment-of-TACTs .

For manuscripts utilizing custom algorithms or software that are central to the research but not yet described in published literature, software must be made available to editors and reviewers. We strongly encourage code deposition in a community repository (e.g. GitHub). See the Nature Portfolio guidelines for submitting code & software for further information.

Data

Policy information about availability of data

All manuscripts must include a data availability statement. This statement should provide the following information, where applicable:

- Accession codes, unique identifiers, or web links for publicly available datasets
- A description of any restrictions on data availability
- For clinical datasets or third party data, please ensure that the statement adheres to our policy

A repository with the compiled simulation outputs can be found here: https://github.com/Longterm-deployment-of-TACTs/Longterm-deployment-of-TACTs .

Research involving human participants, their data, or biological material

Policy information about studies with human participants or human data. See also policy information about sex, gender (identity/presentation), and sexual orientation and race, ethnicity and racism.

Reporting on sex and gender	This work does not involve any real-world data as it is purely theoretical and all data are generated in silico
Reporting on race, ethnicity, or other socially relevant groupings	This work does not involve any real-world data as it is purely theoretical and all data are generated in silico
Population characteristics	This work does not involve any real-world data as it is purely theoretical and all data are generated in silico
Recruitment	This work does not involve any real-world data as it is purely theoretical and all data are generated in silico
Ethics oversight	This work does not involve any real-world data as it is purely theoretical and all data are generated in silico

Note that full information on the approval of the study protocol must also be provided in the manuscript.

Field-specific reporting

Please select the one below that is the best fit for your research. If you are not sure, read the appropriate sections before making your selection.

☐ Life sciences ☐ Behavioural & social sciences ☒ Ecological, evolutionary & environmental sciences

For a reference copy of the document with all sections, see nature.com/documents/nr-reporting-summary-flat.pdf

Ecological, evolutionary & environmental sciences study design

All studies must disclose on these points even when the disclosure is negative.

Study description	Two published independently-built individual-based mathematical models of Plasmodium falciparum transmission and resistance evolution (“MORU” and “PSU”) were used to compare the population-wide benefits of deploying TACTs versus continued ACT use. These are microsimulation models run as daily time-step discrete-event simulations of individuals (humans) who can be infected with Plasmodium falciparum malaria and subsequently pass on their infection to other individuals in the simulation via mosquitoes (which are also explicitly modelled in the MORU model).
Research sample	The parameterizations used here follow those in a previous consensus exercise(Watson, O. J. et al. Lancet Microbe 3, e701-e710), with three key differences: (1) population size was set to one million individuals, (2) private-market drug use was included, and (3) TACTs were included as antimalarial treatment. The inclusion of TACTs required incorporation of their pharmacokinetic properties as well as pharmacodynamic parameters calibrated to treatment efficacies consistent with those found in recent clinical trials van der Pluijm, R. W. et al. Lancet 395, 1345-1360)
Sampling strategy	Twenty-seven historic epidemiological scenarios were evaluated, each assuming unique combinations of all-age malaria prevalence (PfPR) (0.1%, 1%, 10%), treatment coverage (25%, 50%, 75%), and baseline ACT choice (DHA-PPQ, ASAQ, AL). For each historic scenario, we explored three different prospective scenarios: (1) continued ACT use as first-line therapy (FLT); (2) switch to artesunate-mefloquine-piperaquine - ASMQ-PPQ; (3) switch to ALAQ. We analysed additional scenarios where TACTs were introduced late, introduced gradually, or with introduction of ASMQ-PPQ at a time of varying genotype frequency of resistance markers for all three components of this TACT.
Data collection	PSU simulations were run in a high-performance computing cluster (HPC) and stored by Tran Dang Nguyen; MORU simulations were run in a HPC and stored by Bo Gao. Ricardo Aguas compiled all simulations results and stored them on a github repository (https://github.com/Longterm-deployment-of-TACTs/Longterm-deployment-of-TACTs).
Timing and spatial scale	The models assumed a single homogeneously mixing populations and tracks all relevant outcome metrics each day.<br>Each simulation consisted of three stages:<br>1- 10 burn-in years calibrated to each historic scenario during which mutation and within-host selection were not allowed (to allow the model to equilibrate at the appropriate PfPR with no additional drug-resistance markers).The last year of calibration is meant to resemble 2009.<br>2- 15 years during which mutation and selection processes are active. The last year of this period marks the end of the historic scenario and is denoted as ‘year zero’ which is meant to resemble 2024.<br>3- 10-year long prospective scenarios simulating introduction of TACTs or continued use of ACTs.
Data exclusions	No data were excluded
Reproducibility	Each parameter set described in the Sampling Strategy above were run 100 times. All those runs were included in the analyses and contribute towards the reported outcome metrics.

Randomization

Models are fully stochastic.

Blinding

Following the data collection protocol, individuals running the simulations did not unblind any result. Data compilation and parameter set unblinding was done by another person.

Did the study involve field work? ☐ Yes ☒ No

Reporting for specific materials, systems and methods

We require information from authors about some types of materials, experimental systems and methods used in many studies. Here, indicate whether each material, system or method listed is relevant to your study. If you are not sure if a list item applies to your research, read the appropriate section before selecting a response.

Materials & experimental systems

n/a	Involved in the study
☒	☐ Antibodies
☒	☐ Eukaryotic cell lines
☒	☐ Palaeontology and archaeology
☒	☐ Animals and other organisms
☒	☐ Clinical data
☒	☐ Dual use research of concern
☒	☐ Plants

Methods

n/a	Involved in the study
☒	☐ ChIP-seq
☒	☐ Flow cytometry
☒	☐ MRI-based neuroimaging
